# Combinatorial Engineering Enables Photoautotrophic Growth in High Cell Density Phosphite-Buffered Media to Support Engineered *Chlamydomonas reinhardtii* Bio-Production Concepts

**DOI:** 10.3389/fmicb.2022.885840

**Published:** 2022-05-13

**Authors:** Malak N. Abdallah, Gordon B. Wellman, Sebastian Overmans, Kyle J. Lauersen

**Affiliations:** Bioengineering Program, Biological and Environmental Sciences and Engineering Division, King Abdullah University of Science and Technology (KAUST), Thuwal, Saudi Arabia

**Keywords:** microalgae, phosphite, algal biotechnology, waste reuse, metabolic engineering, isoprenoids, terpenoids

## Abstract

*Chlamydomonas reinhardtii* has emerged as a powerful green cell factory for metabolic engineering of sustainable products created from the photosynthetic lifestyle of this microalga. Advances in nuclear genome modification and transgene expression are allowing robust engineering strategies to be demonstrated in this host. However, commonly used lab strains are not equipped with features to enable their broader implementation in non-sterile conditions and high-cell density concepts. Here, we used combinatorial chloroplast and nuclear genome engineering to augment the metabolism of the *C. reinhardtii* strain UVM4 with publicly available genetic tools to enable the use of inorganic phosphite and nitrate as sole sources of phosphorous and nitrogen, respectively. We present recipes to create phosphite-buffered media solutions that enable high cell density algal cultivation. We then combined previously reported engineering strategies to produce the heterologous sesquiterpenoid patchoulol to high titers from our engineered green cell factories and show these products are possible to produce in non-sterile conditions. Our work presents a straightforward means to generate *C. reinhardtii* strains for broader application in bio-processes for the sustainable generation of products from green microalgae.

## Introduction

The model green microalga *Chlamydomonas reinhardtii* has emerged in recent years as a newcomer in the metabolic engineering space due to enabling advances in transgene design (Baier et al., 2018b, 2020) and the use of nuclear mutants with enhanced transgene expression rates (Neupert et al., 2009). The alga contains three genomes: nuclear, chloroplast, and mitochondrial; all of which have been shown to be transformable (Kindle et al., 1989; Goldschmidt-clermont, 1991; Remacle et al., 2006). The nuclear genome exhibits integration of foreign transgenes largely by non-homologous end joining (NHEJ), whereas the plastid genome is amenable to homologous recombination and targeted genetic modifications (Rochaix, 1995). *C. reinhardtii* has been extensively used as a host for chloroplast genome engineering for the expression of recombinant proteins for several years (Wannathong et al., 2016; Dyo and Purton, 2018). However, this alga has historically demonstrated recalcitrance to nuclear transgene expression, owing to genetic architectures with high guanine-cytosine (GC) nucleotide content and intron density, random integration of transgenes into the nuclear genome, as well as a recently characterized epigenetic silencing mechanism (Neupert et al., 2020). Through a series of mutational events, strains UVM4 and UVM11 were generated, which exhibited improvements in transgene expression over others (Neupert et al., 2009; Barahimipour et al., 2016). UVM4 has become a workhorse strain for demonstrations of efficient transgene expression, with examples of heterologous production of sesquiterpenes (Lauersen et al., 2016; Wichmann et al., 2018), diterpenes (Lauersen et al., 2018; Einhaus et al., 2021), and polyamines (Freudenberg et al., 2021), modified fatty acid and alkene contents (Yunus et al., 2018), secreted recombinant proteins (Lauersen et al., 2013a,b, 2015a; Baier et al., 2018a), and altered pigment composition (Perozeni et al., 2020). The reduced epigenetic silencing of this strain, coupled with improvements of synthetic intron-addition transgene design strategies (Baier et al., 2018b, 2020) and optimized regulatory element combinations (Scranton et al., 2016; Einhaus et al., 2021), has resulted in increased momentum for algal synthetic biology and green biotechnology applications with these hosts (Lauersen, 2019). *C. reinhardtii* represents a model green microalga that has very well-developed molecular tool kits, including optimized (Lauersen et al., 2015b; Wichmann et al., 2018) and modular cloning (MoClo) plasmids (Crozet et al., 2018). Although a great deal of advancement has been made in gene expression and genetic engineering design, major limitations to scalable cultivation of *C. reinhardtii* remain, which limits the broader development of engineered algal bio-processes.

Cultivation of *C. reinhardtii* is conducted at neutral pH, which means that the protein-rich algal cells are subject to rapid contamination/predation in non-sterile conditions. Sterility is difficult to maintain in large-scale cultivation concepts or in complicated bio-processes. Other industrially cultivated algae have features, such as extreme pH or salinity tolerance, which allow cultivation in selective conditions, or are dominant fast-growing, aggressive species. Although fast growing, the UVM4 strain will not grow if nitrate is the only nitrogen source, similar to many lab-adapted strains of this organism. The use of nitrate is common in larger-scale algal cultivation media, as this nitrogen source does not cause significant pH shifts during its consumption. Complementation of the nitrate metabolism mutation is also important for increasing cell densities in cultivations, as has been recently demonstrated for the production of polyamines from this host (Freudenberg et al., 2021). The risk of contamination in addition to lack of nitrate metabolic capability makes UVM4 deficient in features, which would enable its broader use as an engineered algal green-cell factory outside of proof-of-principle laboratory experiments. One strategy for reducing contamination of algal cultures is the introduction of capacity for metabolism of inorganic phosphite as a phosphorous source. This has been shown in numerous organisms, including *C. reinhardtii*, to reduce contamination and act as a selection agent (López-Arredondo and Herrera-Estrella, 2012; Loera-Quezada et al., 2016; Changko et al., 2020; Cutolo et al., 2020; Dahlin and Guarnieri, 2022). Engineered expression of *Pseudomonas stutzeri* WM88 phosphite NAD^+^ oxidoreductase *ptxD* from either the chloroplast or nuclear genomes of algae has been shown to confer the ability to metabolize phosphite (López-Arredondo and Herrera-Estrella, 2012; Changko et al., 2020; Cutolo et al., 2020; Dahlin and Guarnieri, 2022).

To date, demonstrated advances in nuclear transgene expression for metabolic engineering described above have not incorporated combinatorial engineering with chloroplast expression constructs in the same strain. Here, we combined published advances in chloroplast engineering (Changko et al., 2020) with multiple nuclear engineering steps in UVM4 to demonstrate growth of this strain in phosphite- and nitrate-containing media while producing a proof-of-concept heterologous sesquiterpenoid. We present recipes to enable high-cell density cultivation in phosphite-buffered media and show that these modifications result in comparable heterologous metabolite production in the presence of microbial contamination. Our work shows that advances in nuclear and chloroplast engineering can be combined to yield strains capable of expanded metabolic capabilities that enable modified nutrient use to support heterologous production concepts. These strategies may encourage future bioprocess designs with engineered algal hosts.

## Materials and Methods

### Algal Cultivation and Growth Measurements

*Chlamydomonas reinhardtii* strain UVM4 was used as the parental strain for transformations. This strain was derived from several rounds of mutation in the parental CC-4350 by Dr. Juliane Neupert in the lab of Prof. Dr. Ralph Bock (Neupert et al., 2009) and contains a mutation in Sir2-type histone deacetylase (SRTA), which enables improved transgene expression rates from the algal nuclear genome (Neupert et al., 2020). UVM4 is not able to use nitrate as a nitrogen source due to *nit1*/*nit2* locus mutations (Freudenberg et al., 2021). Microalgal cultures were routinely maintained in a Tris acetate phosphate (TAP) medium (Gorman and Levine, 1965) with updated trace element solution (Kropat et al., 2011) and maintained under 150 μmol m^−2^ s^−1^ mixed cold and warm LED lights with 120–190 rpm agitation in shake flasks or microtiter plates. Light intensities and spectra were measured with a handheld spectrometer (Spectromaster C-7000, Sekonic). Ammonium in TAP salts solution was replaced with equimolar NaNO_3_ to make TAP-NO_3_. Replacements of phosphate with phosphite to make TAPhi and TAPhi-NO_3_ media are described in Supplementary Material 1.

A high-density 6xP medium was prepared as described in Freudenberg et al. (2021), and buffered phosphite solutions to match molar concentrations of phosphorous to make a 6xPhi medium are as described in Supplementary Material 1. All phosphite solutions were filter sterilized and added to media after autoclaving. Cultivation in CellDeg HD100 cultivators (CellDeg GmbH, Germany) was performed with the indicated light and CO_2_ regimes by the growth-control unit using either 6xP or 6xPhi media. Precultures were conducted as follows: 20 ml of the late-exponential phase (~1.5 × 10^7^ cells mL^−1^) *C. reinhardtii* pre-cultured in TAPhi-NO_3_ was centrifuged and resuspended in either 6xP or 6xPhi media. The cells were diluted 1:2 with a fresh medium, and 5 mL was added to 95 ml in the CellDeg reactor at T0. Illumination was delivered by a Valoya broad spectrum LED board supplied by CellDeg GmbH (Germany, the spectrum is presented in Supplementary Material 2).

Growth of algae and contaminants was analyzed by flow cytometry using an Invitrogen Attune NxT flow cytometer (Thermo Fisher Scientific, UK) equipped with a 488 nm blue laser for forward-scatter and side-scatter measurements, and a 695/40 nm filter to detect chlorophyll and non-fluorescent particles, respectively. All culture samples were diluted 1/100 with 0.9% NaCl solution and measured in technical triplicates using previously described settings (Overmans and Lauersen, 2022).

### Plasmids, Algal Transformation, and Screening for Phosphite and Nitrate Metabolism

Plasmids used in this study are listed in Supplementary Table 1. All cloning and plasmid linearization was performed with Thermofisher FastDigest restriction enzymes, New England Biolabs Quick Ligase, and Q5 polymerase following the manufacturer's protocols. Plasmids were maintained in chemically competent *Escherichia coli* DH5a transformed by heat shock. Glass bead transformation of *C. reinhardtii* was performed as previously described for both chloroplast and nuclear-targeted genetic constructs (Kindle, 1990; Kindle et al., 1991). Chloroplast transformation of the pPO3 plasmid [(Changko et al., 2020) graciously provided by Prof. Saul Purton] was performed following the protocol described in Economou et al. (2014) with the following modifications: 10 μg circular DNA and 0.1 mm diameter glass beads rather than 0.424–0.600 mm as commonly used for nuclear transformation (Economou et al., 2014). Recovery was performed in 45 mL TAPhi liquid for 5 days with 150 μE PAR prior to plating. Selection was achieved by plating on TAPhi agar plates incubated at 200 μE for 2–3 weeks. Transformation and selection resulted in only 3–10 colonies per event, and multiple cycles of transformations were used to collect ~20 colonies capable of growth in liquid TAPhi. Colonies were then grown in TAPhi liquid in microtiter plates until green for 1 week, but were not checked for homoplasmy before next transformations.

One transformant with clear growth in liquid TAPhi, hereafter named UVM4-Phi, was transformed for complementation of nitrate metabolic capacity by co-transformation of linearized pMN24 (Fernández et al., 1989) and pMN68 (Schnell and Lefebvre, 1993; Chlamydomonas Resource Center, https://www.chlamycollection.org) by glass beads as previously described (Freudenberg et al., 2021) with overnight recovery and subsequent selection on TAPhi-NO_3_ agar plates. Transformant colonies recovered on TAPhi-NO_3_ plates were recovered after 2 weeks. Resultant colonies were then compared in liquid media in 24-well microtiter plates with standard lighting conditions at 180 rpm. Homoplasmy of the pPO3 integration into the chloroplast genome was determined only in the colonies, which showed growth in this medium, as Phi and NO_3_ metabolic capacity was used as a main selection criterion. Homoplasmy was determined in final strains by PCR using primers Fw: AATTGTATGGGCTCACAACAAACTTAAAGT and Rv: TAAAATTGTGAGACCATGAGTAATGTTCCTCC. The resulting transformants were also screened by an iodine vapor assay as previously described (Wichmann et al., 2018) to determine if random integration had caused starch synthesis modifications. Modified UVM4 transformants, which grew with phosphite and nitrate, are referred to as UVM4-phosphite-nitrate (UPN) strains.

Efficiency of nuclear transgene expression of intermediate strains was investigated by glass bead transformation of the pOpt2_mVenus_Paro plasmid (Wichmann et al., 2018) followed by selection on each respective modified medium with 10 mg L^−1^ paromomycin and fluorescent reporter expression analysis. Fluorescent mVenus expression intensities were analyzed by picking primary transformant colonies using a PIXL robot (Singer Instruments, UK) to 384 colonies/plate layout on manufacturer-supplied rectangular Petri dishes. After 1 week, colonies were replicated using the Singer Instruments ROTOR to generate imaging-ready colonies. White-light pictures of algae colony plates were taken in the built-in PIXL camera. Chlorophyll and mVenus fluorescence signals were captured in an Analytik Jena Chemstudio Plus gel doc with an eLite halogen light source and excitation filters. Chlorophyll fluorescence was captured by 475/20 nm excitation with orange DNA gel emission filter with 1 s exposure, while an mVenus signal was captured with 510/10 nm excitation and 530/10 nm emission filter with 30 s exposure.

### Generation of Patchoulol-Producing UPN Transformants

Plasmids for algal nuclear genome-based expression of the *Pogostemon cablin* patchoulol synthase (*Pc*PS, UniProtQ49SP3) were adapted from (Lauersen et al., 2016). The gene expression cassette for *Pc*PS expression was modified from the pOpt2 vector concept of (Wichmann et al., 2018) to contain transgene designs presented in Baier et al. (2020), Einhaus et al. (2021), and Freudenberg et al. (2021). Briefly, *Pc*PS expression here was driven by the hybrid heatshock 70A beta tubulin promoter described by Einhaus et al. (2021), and the mVenus cassette was modified to contain two copies of the *C. reinhardtii* ribulose-1,5-bisphosphate carboxylase/oxygenase small subunit (RBCS2) intron 1. The RBCS2 intron 2 was moved into the C-terminal strep-II tag of the gene-of-interest expression cassette in the pOpt2_mVenus_Paro plasmid to match that recently described (Baier et al., 2020). This plasmid confers paromomycin resistance in *C. reinhardtii* (Wichmann et al., 2018). *Pc*PS was subcloned into *Bam*HI-*Bgl*II, and 2X, 3X, and 4X *Pc*PS expression cassettes were built by *Sca*I-*Bgl*II inserts from the previous plasmid subcloned into *Sca*I-*Bam*HI of the progenitor plasmid described in Supplementary Figure 1. All constructs contain the C-terminal mVenus (YFP) fusion, which enabled plate-level fluorescence detection in UPN colonies picked by the PIXL robot. *C. reinhardtii* squalene synthase (UniProt A8IE29) knockdown was achieved by secondary transformation using the previously described pOpt2_*c*CA-*g*Luc_i3-SQS_Spect plasmid (Wichmann et al., 2018). UPN *Pc*PS-YFP + SQS k.d. double transformants were selected on TAPhi-NO_3_ agar media containing 10 mg L^−1^ paromomycin and 200 mg L^−1^ spectinomycin as previously described (Wichmann et al., 2018). YFP and luciferase signals of UPN colonies were captured in the ChemstudioPLUS with previously described buffers and reagents for *Gaussia princeps* luciferase bioluminescence analysis (Lauersen et al., 2013a). Full-length target recombinant protein was determined by SDS PAGE and in-gel fluorescence of whole cell pellets in the Chemstudio PLUS with YFP filters described for colony screening. All plasmid sequence files used in this work are given in Supplementary Material 3.

#### Gas Chromatography Analysis of Patchoulol Productivity

UPN transformants expressing *Pc*PS variants were screened for heterologous patchoulol productivity by cultivation in 4.5-ml TAPhi-NO_3_ media with 500 μl dodecane overlay in triplicate for 6 days as previously described (Lauersen et al., 2018). Six individual transformants were investigated for each plasmid construct or combination after fluorescence, or fluorescence and luciferase, screening at the agar-plate level. Dodecane samples were collected from cultures; 90 μl of each collected dodecane sample was transferred into triplicate GC vials. A patchoulol standard (18450, Cayman Chemical Company, USA) calibration curve in the range 10–200 μM patchoulol in dodecane was used for linear-range quantification. 250 μM of α-humulene (CRM40921, Sigma-Aldrich, USA) was added as an internal standard to each dodecane sample and patchoulol standard. Quantification methods and calculations are shown in Supplementary Material 4. The dodecane samples were analyzed using an Agilent 7890A gas chromatograph (GC), equipped with a DB-5MS column (Agilent J&W, USA) attached to a 5975C mass spectrometer (MS) with a triple-axis detector (Agilent Technologies, USA). A previously described GC oven temperature protocol was used (Overmans and Lauersen, 2022). All GC-MS measurements were performed in triplicate (*n* = 3), and chromatograms were manually reviewed for quality control. Gas chromatograms were evaluated with MassHunter Workstation software version B.08.00 (Agilent Technologies, USA).

### Test of Intentional Contamination in Cultures

To test the ability of engineered *C. reinhardtii* UPN lines to withstand contamination in non-sterile conditions using media modifications presented in this work, cultivation was performed with intentional yeast contamination. TAP-NO_3_, TAPhi-NO_3_, 6xP, and 6xPhi media were used to cultivate an engineered SQS k.d. + 2X*Pc*Ps expressing UPN transformant. *Saccharomyces cerevisiae* (yeast) cells were cultured in a yeast extract-peptone-dextrose (YPD) medium (Cold Spring Harbor Protocols) overnight at 28°C. The following day, pelleted cells were resuspended in 300 ml 6xP or 6xPhi media. The 2X*Pc*PS-SQS k.d. strain was cultivated in TAPhi-NO_3_ until a mid-late exponential phase (1.7 × 10^7^ cells mL^−1^); 50 mL was spun down and resuspended with either 6xP or 6xPhi media. The cells were washed two times with the target test medium, and then diluted into 300 mL of the same media. About 4 mL of these dilute cultures was added to microtiter plates for each condition as described. Triplicate wells in 6-well microtiter plates containing 4 mL dilute UPN patchoulol culture in 6xP or 6xPhi media were inoculated with either 500 μl of yeast solutions (above) or a clean medium as controls. About 500 μl of an n-dodecane overlay was also added to each well. Approximately 3 ml of a concentrated potassium bicarbonate buffer was added between the wells to provide a dilute CO_2_ atmosphere as previously described (Dienst et al., 2020). Cultures in TAP-media were grown for 6 days and 6xP/Phi for 9 days on laboratory shakers at 120 rpm with a 12-h:12-h light:dark cycle (150 μE). Cell densities and patchoulol productivities were analyzed as described above.

## Results

### Phosphite and Nitrate Metabolism Can Be Combined in the Nuclear Mutant UVM4

The *C. reinhardtii* mutants UVM4 and UVM11 (Neupert et al., 2009) exhibit reduced transgene silencing due to a mutation in the in Sir2-type histone deacetylase (SRTA; Neupert et al., 2020). UVM4 has served as a powerful parent strain for many recent examples of metabolic engineering in this green microalga (Lauersen et al., 2016, 2018; Wichmann et al., 2018; Einhaus et al., 2021; Freudenberg et al., 2021). Despite its value for past experiments, the alga is grown at neutral pH and contains mutations in its nitrate metabolism, which prevent use of this nitrogen source. These two features manifest in high risk of microbial contamination and the inability to use industrially relevant culture media (Changko et al., 2020; Freudenberg et al., 2021). To prepare UVM4 strains for broader applications, we set to complement it with the capacity to use phosphite as a P source and nitrate as an N source.

**Figure 1:**
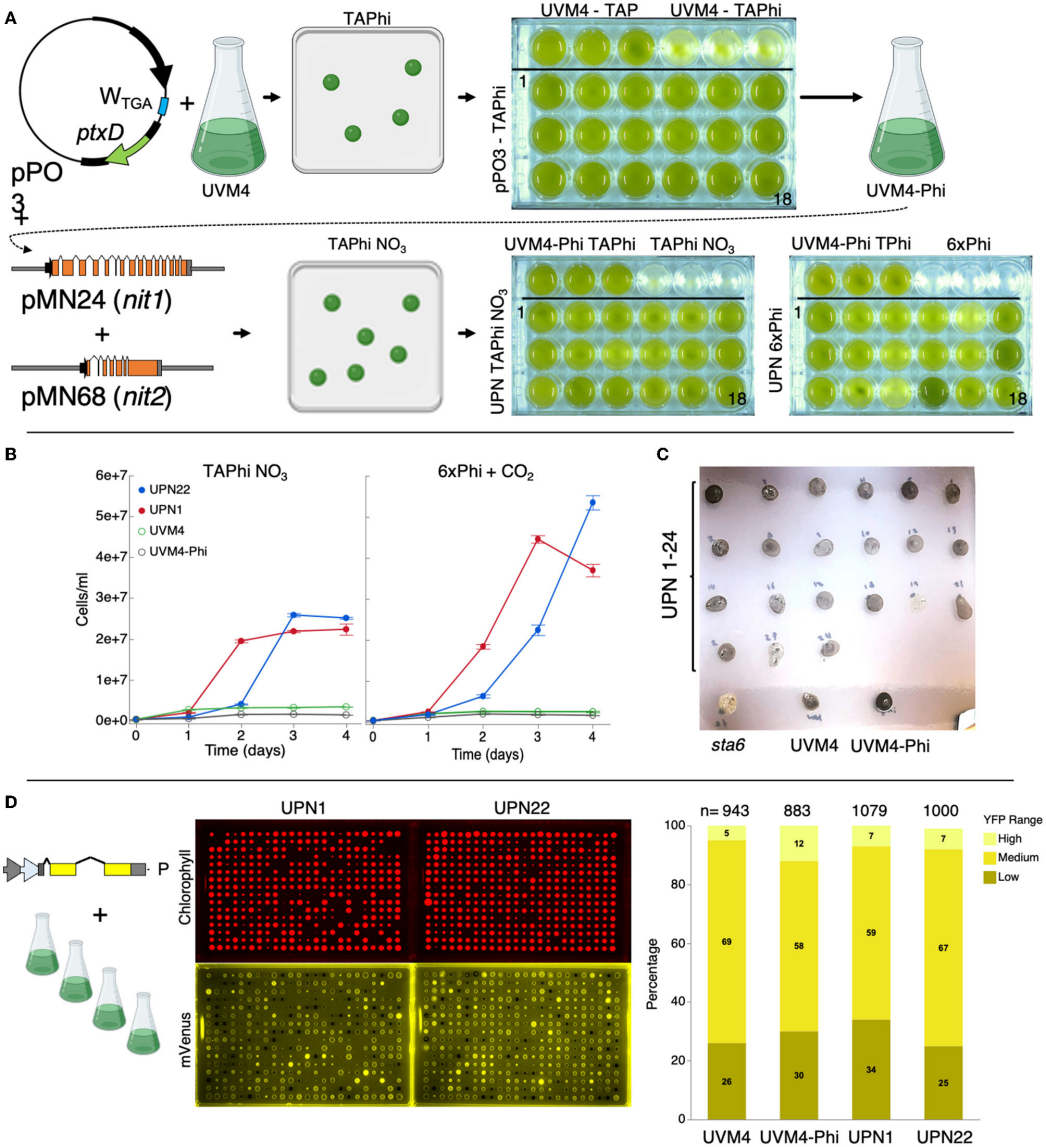
Complementation of *C. reinhardtii* strain UVM4 for growth on phosphite and nitrate. **(A)** Plasmid pPO3 (Changko et al., 2020) was transformed into UVM4, and colonies were recovered on a TAPhi agar medium. Colonies were then cultivated in a TAPhi liquid medium, and one strain (UVM4-Phi) was selected for further complementation with pMN24 (Fernández et al., 1989) and pMN68 (Schnell and Lefebvre, 1993) (*nit1*/*nit2*) plasmids. Selection was performed on TAPhi-NO_3_ plates, and resultant colonies capable of growth on phosphite and nitrate (UPN) were cultivated in liquid mixotrophic (TAPhi-NO_3_) and autotrophic (6xPhi) media. Parental strains were grown as reference in each previous stage media as shown. **(B)** Growth curves of liquid cultures of selected colonies, which performed well in TAPhi-NO_3_ or 6xPhi media. Cell concentrations represent the mean (± standard error mean) of three biological replicates per condition, each measured in three technical replicates. Parental strains UVM4 and UVM4-Phi were not able to proliferate in the nitrate phosphite-containing media. **(C)** UPN strains were investigated by iodine vapor staining at the agar plate level to determine if the transformation of three plasmids above had caused background mutations in starch synthesis. Dark color of colonies indicates presence of starch; yellow or light color indicates perturbed starch metabolism as shown for the starchless *sta6* (Zabawinski et al., 2001) mutant. Lighter starch staining is observed in UPN strains 19 and 23. **(D)** Strains UVM4, UVM4-Phi, UPN1, and UPN22 were transformed with the pOpt2_mVenus_Paro plasmid (cartoon), conferring paromomycin resistance, and expressing the mVenus (a YFP reporter). High-throughput robotic colony picking and fluorescence imaging were used to benchmark YFP expression across the transfomant population. Chlorophyll fluorescence (red) was used to identify true colonies, and YFP fluorescence (yellow) was graded for intensity of signal and plotted-comparing numbers of high, medium, and low or no expression (right). Individual colonies analyzed for each transformation event summed from several plates are indicated for each strain. *C. reinhardtii* genetic elements*:* A – HSP70A promoter, R – RBCS2 promoter, i1 – RBCS2 intron 1, i2, RBCS2 intron 2, ß – beta tubulin promoter and its 5' untranslated region (UTR), 3'UTR – RBCS2 3' UTR. Erlenmeyer flask cartoon from BioRender.

We transformed UVM4 with plasmid pPO3 (Changko et al., 2020) to express the *P. stutzeri* WM88 phosphite NAD^+^ oxidoreductase *ptxD* (López-Arredondo and Herrera-Estrella, 2012) that converts inorganic phosphite into organic phosphate from the algal chloroplast genome (Figure 1A). We found it was possible to transform UVM4 with this chloroplast genome-integrating plasmid by glass bead transformation and select colonies on a TAPhi medium with no additional selection pressure. A resulting UVM4-Phi transformant was then subsequently transformed with pMN24 and pMN68 plasmids, which contain genomic copies of the *nit1* and *nit2* loci, respectively, to complement nitrate metabolism capacity (Fernández et al., 1989; Schnell and Lefebvre, 1993; Figure 1A).

Colonies were recovered by selection on TAPhi-NO_3_ plates with no antibiotic (Figure 1A). Complementation with *nit1*/*2* can sometimes lead to colonies that survive on the agar plate, but do not perform well in a liquid medium. Therefore, we also benchmarked performance of 24 UVM4-phosphite-nitrate (UPN) colonies derived from these transformations in TAPhi-NO_3_ and photoautotrophic cultivation with CO_2_ as a carbon source (Figure 1A, lower right). UPN strains grown in a liquid medium with nitrate exhibited variable performance, especially in photoautotrophic conditions (Figures 1A,B). In the TAP medium, with ammonium and phosphate, UVM4-Phi and UPN strains were found to reach lower cell densities than UVM4. These strains exhibited lower portions of small cell debris and attained reasonable cell densities (Supplementary Figure 2). Most colonies maintained normal starch accumulation, which was qualitatively assessed by iodine vapor; however, Colonies 19 and 23 showed reduced iodine staining (Figure 1C). Homoplasmy of pPO3 integration was determined in UVM4-Phi and nitrate-complemented strains (Supplementary Figure 3).

To confirm that the three plasmid integrations did not modify the performance of the parent UVM4 capacities for nuclear transgene expression, several UPN strains with acceptable growth in liquid phosphite-nitrate media were transformed with a YFP reporter plasmid (Wichmann et al., 2018). High-throughput robotics-assisted colony picking allowed analysis of between 943 and 1,079 colonies for each strain, and plate-level fluorescence imaging was used to quantify reporter expression across the populations. YFP reporter expression efficiencies in the final UPN strains were comparable to parent UVM4 (Figure 1D).

### Phosphite Can Replace Phosphate in Buffered Media for High Cell-Density Cultivation of Algal Cells

**Figure 2:**
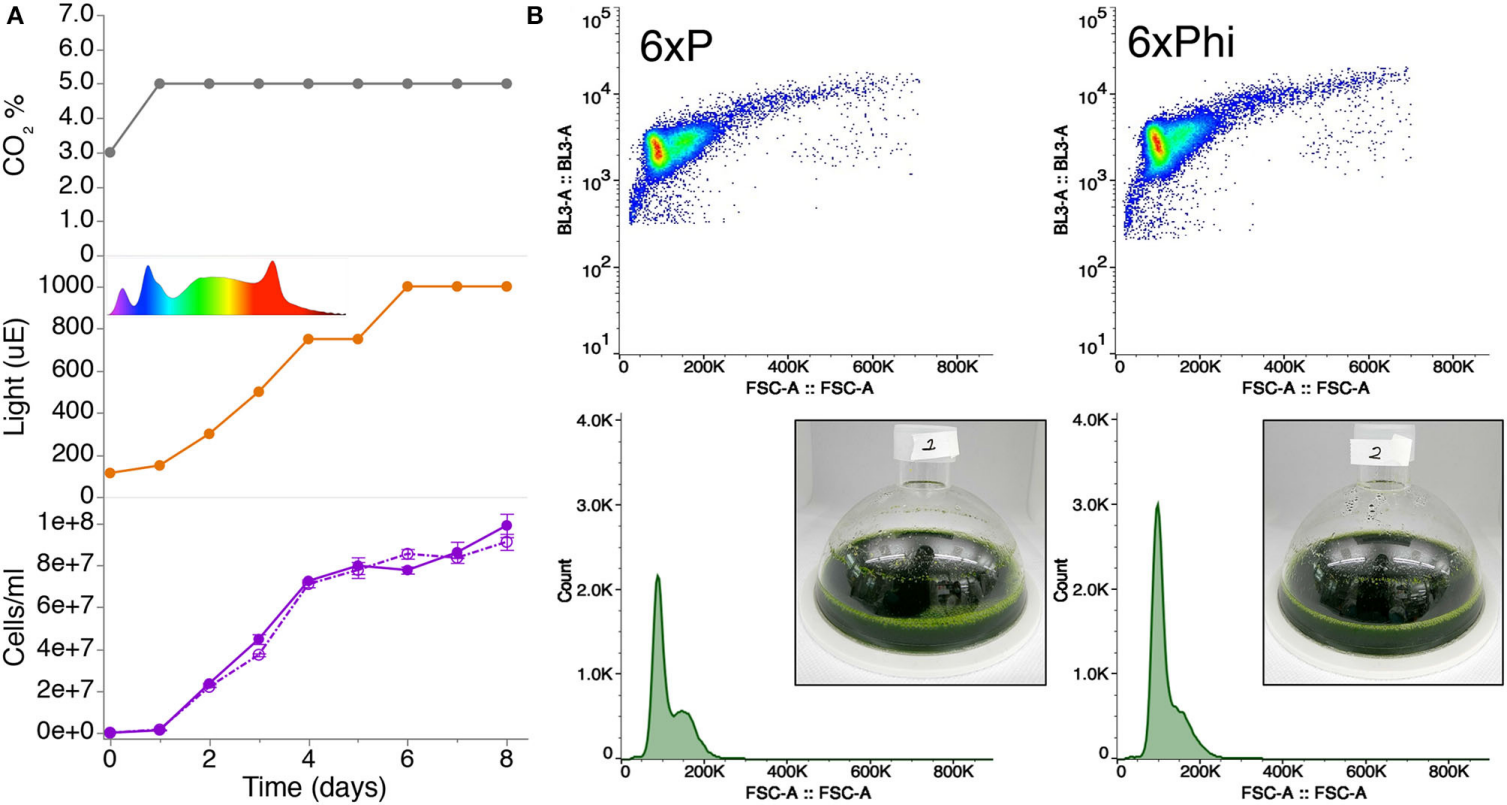
Buffered phosphite solutions can be used in algal high-cell density medium concepts to replace phosphate. **(A)** Growth of strain UPN22 was tracked in cultivations in 100 ml of 6XP (solid line) or 6XPhi (dashed line) media in CellDeg HD100 cultivators, following the CO_2_ and light regime indicated. The spectrum of the Valoya daylight lamp is shown. Cell densities were recorded daily. Values represent mean (± standard error mean) of three technical replicates per reactor and sampling point. **(B)** Forward and backscatter plots from flow cytometry of samples from day 6 of each culture with photographs of the dense green culture in either medium.

Using mono- and di-basic forms of phosphite, we generated a buffered phosphite solution to emulate the phosphate buffer solution of a recently published 6xP medium (Freudenberg et al., 2021; Supplementary Material 1). In order to compare whether our high-density phosphite medium (6xPhi) could be used in a comparable fashion to 6xP, we benchmarked growth of a UPN strain in a high-density cultivation concept using high-light and membrane-delivered CO_2_ in CellDeg HD100 cultivators (Figure 2A). We did not observe differences in performance for the UPN strain cultivated in 6xP or 6xPhi, which reached comparable cell densities throughout cultivation (Figure 2B).

### Heterologous Products Can Be Efficiently Made in UPN Strains

**Figure 3:**
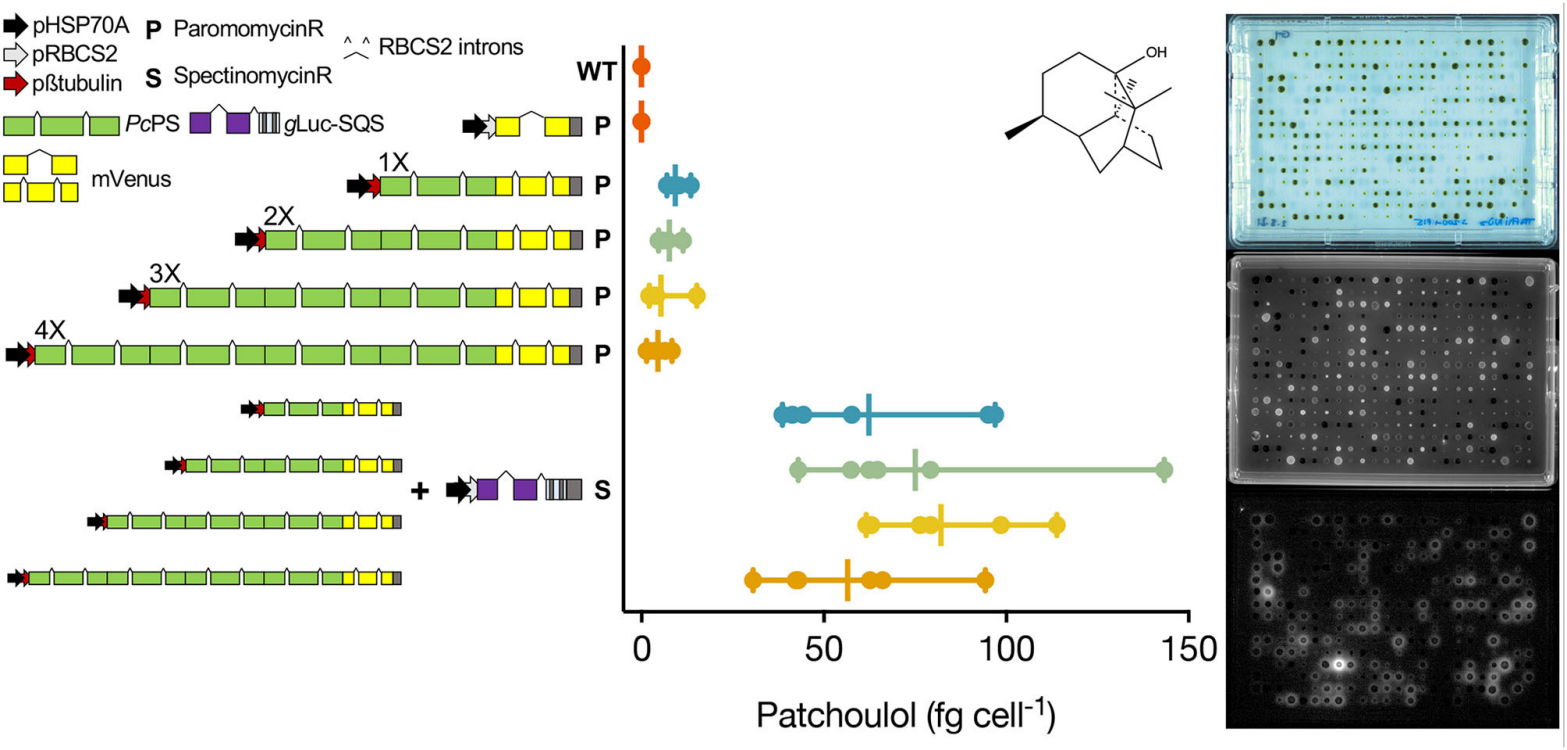
Genetic constructs used to generate heterologous patchoulol production from a UPN strain. Single, double, triple, and quadruple copies of the *C. reinhardtii* codon optimized, intron containing *P. cabiln* patchoulol synthase were fused to generate different expression plasmids with C-terminal mVenus (YFP) reporter fusions as previously described (Lauersen et al., 2016). Each plasmid was transformed into UPN, and mVenus (YFP)-expressing colonies were isolated for each construct and benchmarked for patchoulol production (*n* = 6). Patchoulol production was determined from six biological replicates (*n* = 6), each measured in technical triplicates. Vertical bars show the mean and horizontal bars indicate the range of values. The chemical structure of patchoulol is shown. The best-performing individual from each plasmid was then subsequently transformed with a plasmid expressing a luciferase-amiRNA construct, which downregulates the *C. reinhardtii* squalene synthase. Combined high-throughput fluorescence and luciferase screening of colonies led to isolation of strains with both constructs expressed (*n* = 6), which were then subsequently benchmarked for patchoulol productivity.

We then chose to combine two proven engineering strategies for sesquiterpenoid production from a UPN strain grown only in the TAPhi-NO_3_ medium. The *C. reinhardtii* codon-optimized *P. cablin* patchoulol synthase (*Pc*PS) was expressed in 1, 2, 3, and 4X copy fusion protein constructs with C-terminal YFP from the nuclear genome of this alga (Figure 3). Robotics-assisted colony picking and YFP screening allowed selection of six transformants per plasmid with confirmed *Pc*PS expression, which were benchmarked for patchoulol productivity as previously described (Lauersen et al., 2018). The best-performing transformants were subsequently transformed with a secreted luciferase-artificial-micro-RNA expression construct, targeting the *C. reinhardtii* squalene synthase (SQS; Wichmann et al., 2018). After colony recovery and robotics picking, plate-level imaging was used to isolate colonies with a YFP fluorescence signal (*Pc*PS) and luciferase activity (SQS k.d.; Figure 3). Patchoulol productivity analysis indicated striking improvements in patchoulol productivity for SQS k.d. strains compared to parentals with the best-performing strains, generating ~145 fg patchoulol cell^−1^ (Figure 3). Full-length fusion protein expression could be confirmed only for 1-3X*Pc*PS-YFP constructs by in gel fluorescence (Supplementary Figure 4).

### Nitrate and Phosphite Can Both Assist Contaminant Control in Algal Cultures

**Figure 4:**
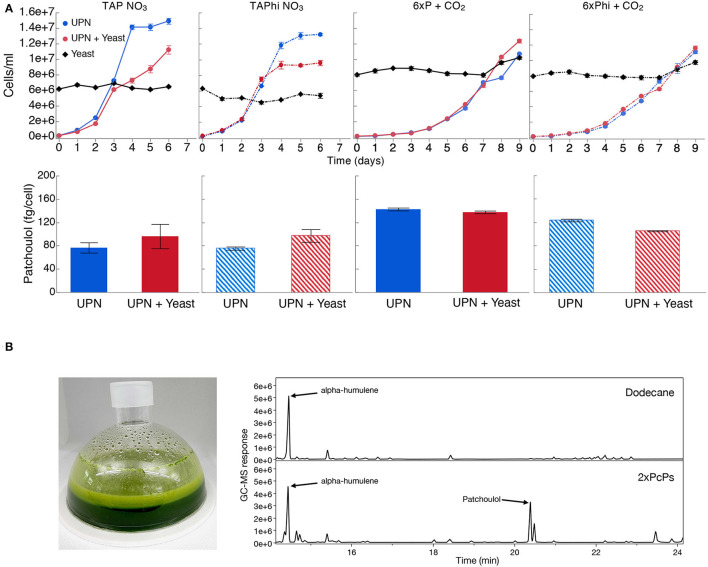
Production of heterologous sesquiterpenoid in the presence of contamination. **(A)** *C. reinhardtii* UPN 22 expressing 2X*Pc*PS-YFP + SQS-amiRNA was cultivated in different trophic modes with and without phosphite and intentionally contaminated with *S. cerevisiae* cells. TAP-NO_3_ and TAPhi-NO_3_ were used to compare mixotrophic conditions where acetic acid was a sole carbon source, while 6xP and 6xPhi were used to test photoautotrophic conditions. All growth curves with Phi are represented with dashed lines and hashed bars. The dodecane overlay was used to capture heterologous patchoulol produced. Yeast cells were intentionally inoculated at high densities to challenge the algal cells to outcompete them in these conditions. CO_2_ was delivered to autotrophic cultures by placing a high-concentration bicarbonate buffer between microtiter plate wells as an inefficient delivery mechanism to further challenge the algal cells. Patchoulol was quantified on the last cultivation day indicated for each growth curve. Error bars in growth curves represent standard error mean from three biological replicates of three technical replicate samples taken per time point. The error bars in patchoulol quantification are the standard error mean of three technical measurements from pooled dodecane samples across biological replicates. **(B)** Cultivation of this strain in a 6xPhi medium in an HD100 cultivator (pictured) with the dodecane overlay resulted in efficient patchoulol production from CO_2_. Two GC-MS chromatograms are shown from the 6th day of cultivation, one of the dodecane blanks with an alpha-humulene internal standard and one from algal culture, indicating the peak of produced patchoulol.

As contamination of cultures can be an issue with neutral pH cultivation, we wanted to determine if phosphite and nitrate could permit algal growth in the presence of contamination. We intentionally contaminated the best-performing UPN *Pc*PS SQS k.d. strain in mixotrophic (acetic acid) and photoautotrophic cultures in media with nitrate as a nitrogen source and either phosphate or phosphite as a phosphorous source. Yeast cells were added to cultures directly in higher cellular abundances than algal cells (Figure 4). In all media conditions, yeast cells did not proliferate, regardless of the presence of organic carbon but also did not reduce in number. When acetic acid (TAP-derived) media were used, algal growth was reduced compared to cultivations without yeast, also with phosphite (Figure 4). No difference in performance was noted in photoautotrophic cultures. In all conditions, the presence of high concentrations of yeasts in cultivations did not inhibit heterologous patchoulol production (Figure 4).

We then benchmarked patchoulol productivity in a 200 mL culture in a membrane gas delivery bioreactor, containing 10% dodecane overlay to capture a heterologous sesquiterpenoid product. Culture volume was adjusted to 200 mL to avoid contact of dodecane with the hydrophobic gas delivery membrane during shaking, and the culture was operated in non-sterile conditions. The culture accumulated up to 6.5 x 10^7^ ± 1.9 x 10^6^ cells mL^−1^ and generated 6.2 mg L^−1^ patchoulol in 6 days using this system.

## Discussion

### Designing Engineerable Strains to Be Ready for Bio-Processes

We chose to introduce phosphite metabolism by transformation of plasmid pPO3 as this also contains extra future chloroplast genome engineering potential through the addition of the W_TGA_ tRNA for tryptophan as previously described (Changko et al., 2020). When filter sterilization was used, transformation and selection on phosphite solutions were greatly improved, and appearance of background algal growth at the plate level was reduced (data not shown). After confirmed growth of pPO3 transformants in liquid phosphite, nitrate metabolism was complemented by transformation of both pMN24 (*NIT1*) and pMN68 (*NIT2*) plasmids in a UVM4-Phi strain. This double transformation is relatively inefficient; nevertheless, we could generate several dozen colonies per transformation, which recovered on nitrate plates. Colonies, which recovered on nitrate plates did, however, not all perform well in liquid culture growth in nitrate-containing liquid media. We chose to move forward with only those colonies that appeared to grow to a dark-green stationary phase (Figure 1A). Colonies were then checked for homoplasmy integration of the pPO3 phosphite metabolism-conferring plasmid (Supplementary Figure 3), and two transformants were benchmarked for their growth in phosphite- and nitrate-containing media compared to their parental strains (Figure 1B).

To determine if our strategy for UVM4 augmentation would allow future engineering to benefit from these metabolic enhancements, two questions remained: 1) was nuclear transformation expression efficiency disturbed during these events in UVM4 derivatives? 2) Can inorganic phosphite be used in a similar way to organic phosphate for buffered media solutions? We benchmarked two fully complemented UPN transformants, their UVM4-Phi parents, and the UVM4 starting strain for YFP efficiency expression from the nuclear genome. Using high-throughput colony picking, we were able to analyze ~1,000 colonies per transformation event and compare YFP expression efficiencies across the populations (Figure 1C). Although some variance, little difference could be seen in the total ratio of high and mid-range YFP expressing colonies, suggesting these three plasmid integrations had not modified nuclear transgene expression capacity from the UVM4 background.

We then set out to make a buffered phosphite solution, which could replace buffered phosphate solutions in culture media (Supplementary Material 1). In direct comparison growth tests, the UPN strain did not show performance differences in phosphite compared to phosphate in photoautotrophic high-density cultivations (Figure 2). Our results indicate the use of inorganic buffered phosphite solutions as media components is not different than phosphate for the augmented strains. As phosphate is a globally dwindling resource important to agriculture, bio-conversion of phosphite into phosphate may also enable the use of this waste mineral to yield bio-fertilizers through engineered algal cultivation.

### Patchoulol Production in Metabolically Augmented Strains

UPN strains were maintained exclusively on the TAPhi-NO_3_ medium for all routine lab work. A further aim was to determine if it was possible to conduct additional metabolic engineering in these strains for heterologous isoprenoid production using Phi-NO_3_ media. Plasmids were constructed based on previous designs to express the patchoulol synthase (*Pc*PS) and localize it in the cytoplasm of the alga where this enzyme is known to convert freely available farnesyl pyrophosphate (FPP) into patchouli alcohol (patchoulol; Lauersen et al., 2016). We chose to combine recently published modifications in promoter (Einhaus et al., 2021) and intron use (Baier et al., 2020; Freudenberg et al., 2021; Supplementary Figure 1) in order to assist recombinant protein accumulation in an effort to enhance product yields. Previous work on *Pc*PS indicated cellular patchoulol productivities could be enhanced when the protein was fused to itself in a repetitive fashion to yield more active sites per translated protein (Lauersen et al., 2016). Here, we copied this gene design strategy and combined it with an artificial micro-RNA (amiRNA) knockdown of the squalene synthase (SQS; Figure 3). It was previously found that SQS k.d. improved (*E*)-α-biabolene titers from the cytoplasm of *C. reinhardtii* as this is the direct competitor for FPP precursor (Wichmann et al., 2018). Additive *Pc*PS units were found here to increase cellular patchoulol yields as previously observed (Lauersen et al., 2016). However, increasing repetitions past 3X*Pc*PS copies were found to be unstable and did not generate reliable patterns in patchoulol production, despite some production being observed (Figure 3, Supplementary Figure 3). As expected, based on past work with bisabolene (Wichmann et al., 2018), addition of the SQS k.d. to the best performing *Pc*PS variant of each plasmid led to transformants with drastic improvements in patchoulol productivity. Previous engineering of patchoulol production for *C. reinhardtii* led to a maximal volumetric productivity of ~350 μg patchoulol L^−1^ in mixotrophic 400 mL bioreactor conditions from a 3X*Pc*PS-YFP transformant (Lauersen et al., 2016). Here, a 2X*Pc*PS transformant subsequently transformed with the SQS k.d.-generated lines, producing 700–1,400 μg patchoulol L^−1^ culture (Figure 3, Supplementary Figures 5, 6). Improvements were observed across 1–4X *Pc*PS-YFP lines by SQS k.d. and from 1–3X*Pc*PS-YFP; mean production increased with increasing *Pc*PS units. Maximal cellular productivity was observed in a single 2X*Pc*PS SQS k.d. line, with up to 143 fg patchoulol cell^−1^ (Figure 3).

A risk to scaled cultivation of engineered *C. reinhardtii* in bio-production concepts is contamination and reduced productivities, which is especially true for cell-wall-deficient strains that may be more readily outcompeted by contaminants. We intentionally contaminated mixotrophic and photoautotrophic media, containing either NO_3_ or Phi and NO_3_ with yeast, and cultivated a UPN-patchoulol-producing strain in these sub-optimal conditions (Figure 4A). We inoculated the 2X*Pc*PS-SQS k.d. UPN strain into media containing 6 x 10^6^ cells mL^−1^ yeast, the same cell density as reached in the mid-log phase for the algal cells. We chose to provide CO_2_ using potassium bicarbonate buffers (Dienst et al., 2020) rather than direct gas delivery to further challenge the phototrophic cultures. In all conditions, yeast cells did not proliferate, with either acetic acid as a carbon source, or in the photoautotrophic media (Figure 4A). Mixotrophic cultures exhibited lower algal cell densities in later stages of cultivation than in the absence of yeast, likely due to the yeast sequestering some of the acetic acid. However, in the establishment phase (days 0–3), growth with yeast was not markedly different than that of cultures without yeast. In photoautotrophic cultures, yeast was inoculated at higher starting cell densities (8 x 10^6^ cells mL^−1^) as there is no organic carbon source. This density was chosen to determine if the yeast cells would competitively inhibit the inoculated algal cells. Here, the UPN strain was able to overtake the yeast cells, demonstrating linear growth in both conditions relative to carbon diffusion rates in the medium. Under all conditions, the presence of yeast contaminants did not hinder the accumulation of heterologous patchoulol in dodecane overlays, which exhibited similar productivities per algal cell (Figure 4A). Our results indicate that both nitrate and phosphite are a powerful combination to limit contaminating microbial competitors in engineered algal cultivation concepts.

To determine if we could produce patchoulol in non-sterile conditions, we cultivated this strain in a CellDEG HD100 bioreactor with dodecane overlay using the 6xPhi medium (Figure 4B). Dodecane impairs the hydrophobic gas delivery membrane of the reactors, so we used 200 mL culture volume to prevent the solvent interacting with the membrane. Previous photoautotrophic yields of this product were only 350 μg L^−1^ in 8 days (Lauersen et al., 2016). Here, without any process optimization, ~6.2 mg patchoulol L^−1^ was produced from CO_2_ in 7 days (Figure 4). A previous study with *Synechocystis* sp. PCC 6803 used a similar membrane gas delivery system for 10-ml cultures to generate up to 17.3-mg patchoulol L^−1^ in 8 days using a two-stage semi batch mode where half of the culture medium was replaced after 96 h. We did not further optimize our production experiments in the HD100, as the risk of dodecane-membrane wetting means cultivation must be performed with volumes not intended for the system. A further issue of the dodecane overlay in turbid algal cultures is the formation of emulsions with hydrophobic cellular components and the dodecane solvent (Lauersen, 2019). The surface interaction of culture and dodecane causes significant emulsion formation in this volume ratio (Figure 4B), which is less pronounced in smaller volume cultivation units. Indeed, better isoprenoid extraction methods are needed for performance benchmarking and production concepts, which do not rely on hard-to-handle solvents. Nevertheless, our results indicated the combination of nitrate and phosphite metabolic capacities enables high-density cultivations of engineered strains to be performed with reduced risks of contamination without affecting process yields. To our knowledge, this is the first demonstration of combinatorial plastid and nuclear genome engineering in a green alga, which helped facilitate growth in non-sterile conditions, coupled with nuclear transgene expression for heterologous metabolite production.

## Conclusion

Here, we have demonstrated the metabolic augmentation of the common UVM4 nuclear mutant with the genetic capacity for phosphite and nitrate metabolism through chloroplast and nuclear transgene integration, respectively. These modifications were possible without affecting the nuclear transgene expression abilities of UVM4. We could subsequently engineer these strains to produce heterologous patchoulol and could cultivate the strain and produce the product in non-sterile conditions. Our work, however, does not address the need for new selection markers, which could help reduce the use of antibiotic resistance genes in engineered strains. Here, we used previously published genetic elements to demonstrate heterologous production of patchoulol for a proof of a concept. Future engineering should address development of novel selection markers with less potential negative impacts. We present a recipe for buffered phosphite solutions to replace those of phosphate in common *C. reinhardtii* media and show improved titers of patchoulol through the combination of strategies known to improve flux to sesquiterpenoid products. Our work can be used as a guide for others to adapt phosphite-nitrate metabolism into their strains and may enhance the transition of lab-scale engineering to less-sterile production concepts.

## Data Availability Statement

The original contributions presented in the study are included in the article/Supplementary Materials, further inquiries can be directed to the corresponding author.

## Author Contributions

MA, GW, and SO performed experiments and contributed to experimental design, methods, and manuscript writing. KL was responsible for experimental design, project scope, funding acquisition, and manuscript writing. All authors contributed to the article and approved the submitted version.

## Funding

Funding for this work was supported by the King Abdullah University of Science and Technology baseline research fund awarded to KL.

## Conflict of Interest

The authors declare that the research was conducted in the absence of any commercial or financial relationships that could be construed as a potential conflict of interest.

## Publisher's Note

All claims expressed in this article are solely those of the authors and do not necessarily represent those of their affiliated organizations, or those of the publisher, the editors and the reviewers. Any product that may be evaluated in this article, or claim that may be made by its manufacturer, is not guaranteed or endorsed by the publisher.

## Supplementary Material

Supplementary Figure 1 Modifications to the pOpt2 plasmids used in this work. Above: An overview of plasmid architecture of the pOpt 2. vectors from Wichmann et al. (2018) 10.1016/j.ymben.2017.12.010. The gene of interest (GOI) reporter cassette is highlighted. Middle: in this work, we modified the GOI cassette to contain the optimized HSP70A, beta-tubulin (and 5'UTR) from Einhaus et al. (2021) 10.1021/acssynbio.0c00632. The mVenus reporter (NCBI: AAZ65844) was also modified to contain two copies of the RBCS2 Intron 1, and the RBCS2 Intron 2 was moved into the C-terminal StrepII tag so that future C-terminal fusions could benefit from this orientation in a similar fashion to that presented in Freudenberg et al. (2021) 10.1016/j.biortech.2020.124542. All elements are from *C. reinhardtii:* A – HSP70A promoter, R – RBCS2 promoter, i1 – RBCS2 Intron 1, i2, RBCS2 Intron 2, ß – beta tubulin promoter and its 5' untranslated region (UTR), 3'UTR – RBCS2 3' UTR. AmpR – ampicillin resistance cassette of the pBluescriptSK (+) backbone. Below: Plasmids for the expression of the *C. reinhardtii* codon optimized and synthetic intron-containing *Pogostemoncablin* patchoulol synthase (UniProt: Q49SP3, *Pc*PS). Modified pOpt2.0 expression cassettes were used to subclone the *Pc*PS, which had been previously codon optimized (including intron spreading) for expression from the nuclear genome of *C. reinhardtii* (NCBI: KX097887, Lauersen et al. (2016) 10.1016/j.ymben.2016.07.013). All plasmids have the pOpt2.0 – paromomycin resistance cassette (P) 3' of the GOI expression cassette pictured as in Wichmann et al. (2018)]. Subcloning of *Pc*PS plasmid 1X was performed in the lab of Prof. Dr. Olaf Kruse by Dr. Julian Wichmann and Dr. Thomas Baier as part of an Institute for Innovation Transfer (IIT) project funded by Lauersen (KAUST). Further subcloning of 2X, 3X, 4X was performed by Dr. Gordon Wellman in Lauersen's lab at KAUST. Y – mVenus expression cassette, 1X, 2X, 3X, 4X plasmids contain the respective numbers of *Pc*Ps copies. Cloning was achieved by using the previous plasmid, with *Sca*I-*Bam*HI as the receiving vector and *Sca*I-*Bgl*II as an insert to amplify the *Pc*PS coding sequence.

Supplementary Figure 2 Growth curve and flow cytometry plots of UVM4, UVM4-Phi, UPN1, and UPN22 grown in 24-well microtiter plates in a TAP medium with ~200 μE light intensity. Left: a plot of cell density over time. Right: the forward scatter against chlorophyll fluorescence plots of flow cytometry on Day 3 of cultivation. Error bars represent the standard error mean across three biological replicates and three technical measurements per sample. The black arrow highlights small cells/debris noticed in UVM4 culture.

Supplementary Figure 3 Confirmation of pPO3 integration into the chloroplast genome of UVM4 in derivative strains UVM4-Phi and UPN22. Primers Fw: AATTGTATGGGCTCACAACAAACTTAAAGT and Rv: TAAAATTGTGAGACCATGAGTAATGTTCCTCC were used to perform PCR on DNA extracts from each strain. The target region without amplification should yield the 1,050-bp band, while integration should yield 3,075-bp products.

Supplementary Figure 4 In gel fluorescence of SDS PAGE samples from one representative mutant of each of the genetic constructs indicated. Fluorescence image was captured with 510/10-nm excitation and 530/10-nm emission filter in the AnalytikJena Chemstudio Plus with eLite. White-contrast, black-and-white image was taken without emission filter using 510/10-nm excitation to visualize the marker.

Supplementary Figure 5 Patchoulol productivities observed in dodecane overlays for six transformants selected for bright YFP fluorescence from each of the above plasmids and compared to parental UPN strain and empty vector (Y)-generated control strains. Numbers at the bottom of the graph correspond to the plasmid name and the mutant number (1.1 = 1X *Pc*PS, transformant #1). Each mutant was analyzed in technical triplicates. Productivity-grouped averages are shown on the left and labeled with each plasmid name. Horizontal bars show the mean, while vertical bars show the range of values.

Supplementary Figure 6 Patchoulol productivities observed in dodecane overlays for six transformants isolated from secondary transformation of the best 1-4X *Pc*PS strains with *c*CA_*g*Luc_i3_SQSk.d. plasmid (Wichmann et al., 2018). Upper right: representitive GC-MS chromatograms showing the drastic increase of patchoulol production in SQS k.d. secondary transformants relative to alpha-humulene internal standard. Lower graphs: Numbers at the bottom of the graph correspond to the plasmid name and the mutant number (1 x 1 = 1 X *Pc*PS SQS k.d. mutant 1). The averages of all transformants per group are shown on the left and labeled with each plasmid name. Corresponding parent performances are also shown from the data in the previous figure.

Supplementary Table 1 Genetic constructs used in this study. Plasmids for transformation of *C. reinhardtii* are shown as well as some of their respective properties. References to plasmid sequences are given, and those generated in this work are provided in the supplement.
