# Supplementary figures and images for: Combinatorial Engineering Enables Photoautotrophic Growth in High Cell Density Phosphite-Buffered Media to Support Engineered Chlamydomonas reinhardtii Bio-Production Concepts
Source: Front Microbiol. 2022 May 13;13:885840. doi: 10.3389/fmicb.2022.885840 (PMC9141048; doi:10.3389/fmicb.2022.885840)

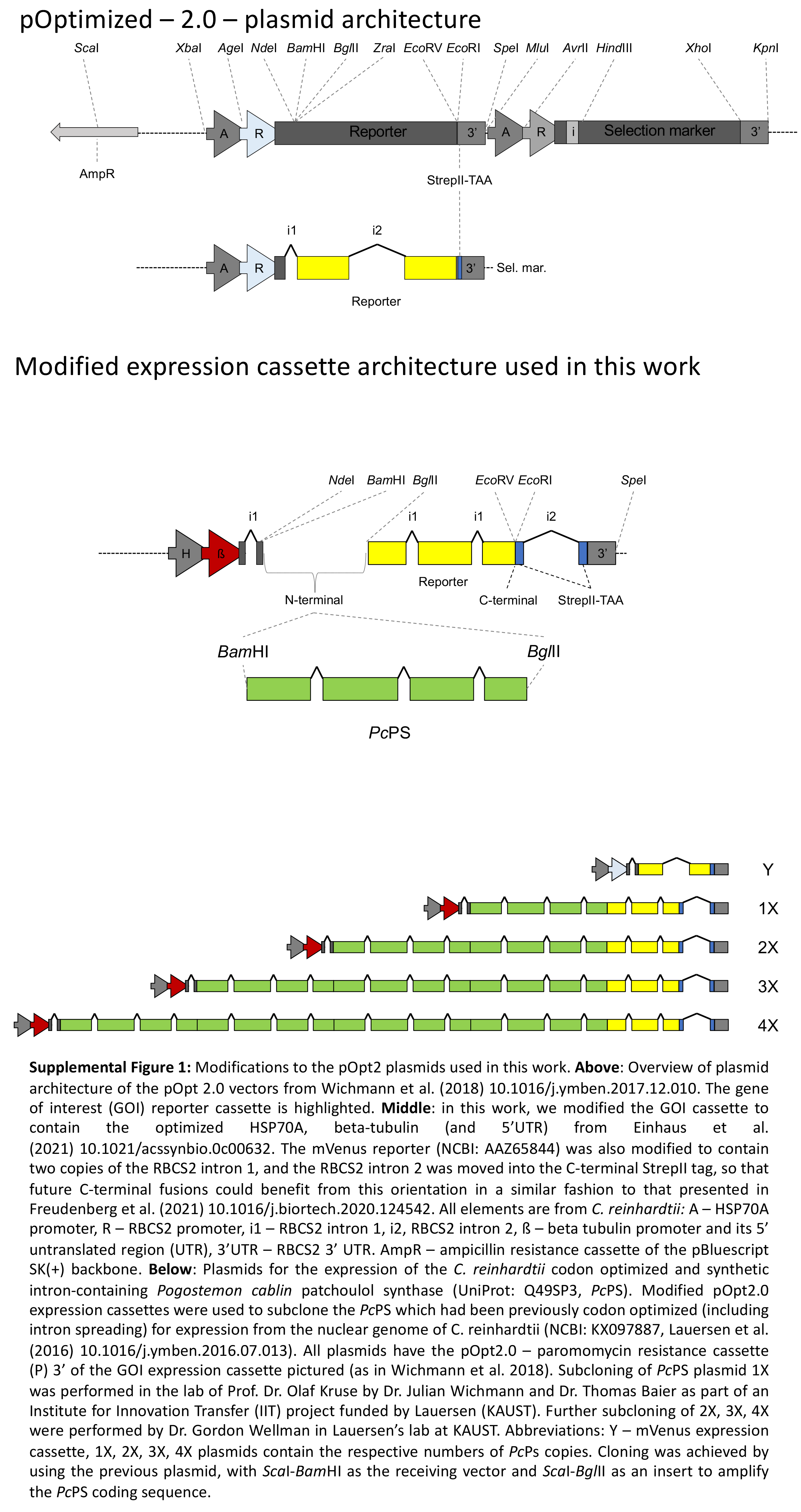

Supplement: Supplementary Figure 1 — Modifications to the pOpt2 plasmids used in this work. Above: An overview of plasmid architecture of the pOpt 2. vectors from Wichmann et al. (2018) 10.1016/j.ymben.2017.12.010. The gene of interest (GOI) reporter cassette is highlighted. Middle: in this work, we modified the GOI cassette to contain the optimized HSP70A, beta-tubulin (and 5'UTR) from Einhaus et al. (2021) 10.1021/acssynbio.0c00632. The mVenus reporter (NCBI: AAZ65844) was also modified to contain two copies of the RBCS2 Intron 1, and the RBCS2 Intron 2 was moved into the C-terminal StrepII tag so that future C-terminal fusions could benefit from this orientation in a similar fashion to that presented in Freudenberg et al. (2021) 10.1016/j.biortech.2020.124542. All elements are from C. reinhardtii: A – HSP70A promoter, R – RBCS2 promoter, i1 – RBCS2 Intron 1, i2, RBCS2 Intron 2, ß – beta tubulin promoter and its 5' untranslated region (UTR), 3'UTR – RBCS2 3' UTR. AmpR – ampicillin resistance cassette of the pBluescriptSK (+) backbone. Below: Plasmids for the expression of the C. reinhardtii codon optimized and synthetic intron-containing Pogostemoncablin patchoulol synthase (UniProt: Q49SP3, PcPS). Modified pOpt2.0 expression cassettes were used to subclone the PcPS, which had been previously codon optimized (including intron spreading) for expression from the nuclear genome of C. reinhardtii (NCBI: KX097887, Lauersen et al. (2016) 10.1016/j.ymben.2016.07.013). All plasmids have the pOpt2.0 – paromomycin resistance cassette (P) 3' of the GOI expression cassette pictured as in Wichmann et al. (2018)]. Subcloning of PcPS plasmid 1X was performed in the lab of Prof. Dr. Olaf Kruse by Dr. Julian Wichmann and Dr. Thomas Baier as part of an Institute for Innovation Transfer (IIT) project funded by Lauersen (KAUST). Further subcloning of 2X, 3X, 4X was performed by Dr. Gordon Wellman in Lauersen's lab at KAUST. Y – mVenus expression cassette, 1X, 2X, 3X, 4X plasmids contain the respective numbers of PcPs co [file Image_1.TIF]

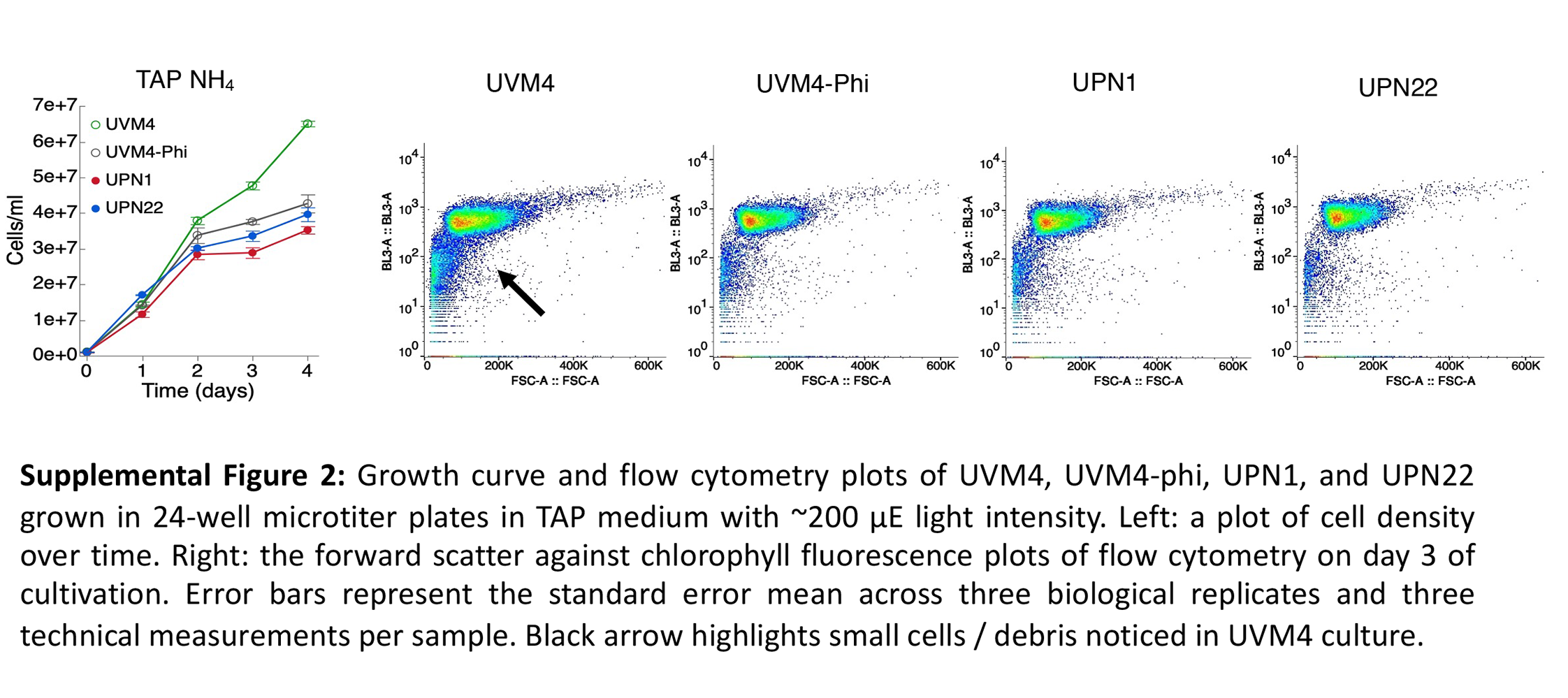

Supplement: Supplementary Figure 2 — Growth curve and flow cytometry plots of UVM4, UVM4-Phi, UPN1, and UPN22 grown in 24-well microtiter plates in a TAP medium with ~200 μE light intensity. Left: a plot of cell density over time. Right: the forward scatter against chlorophyll fluorescence plots of flow cytometry on Day 3 of cultivation. Error bars represent the standard error mean across three biological replicates and three technical measurements per sample. The black arrow highlights small cells/debris noticed in UVM4 culture. [file Image_2.TIF]

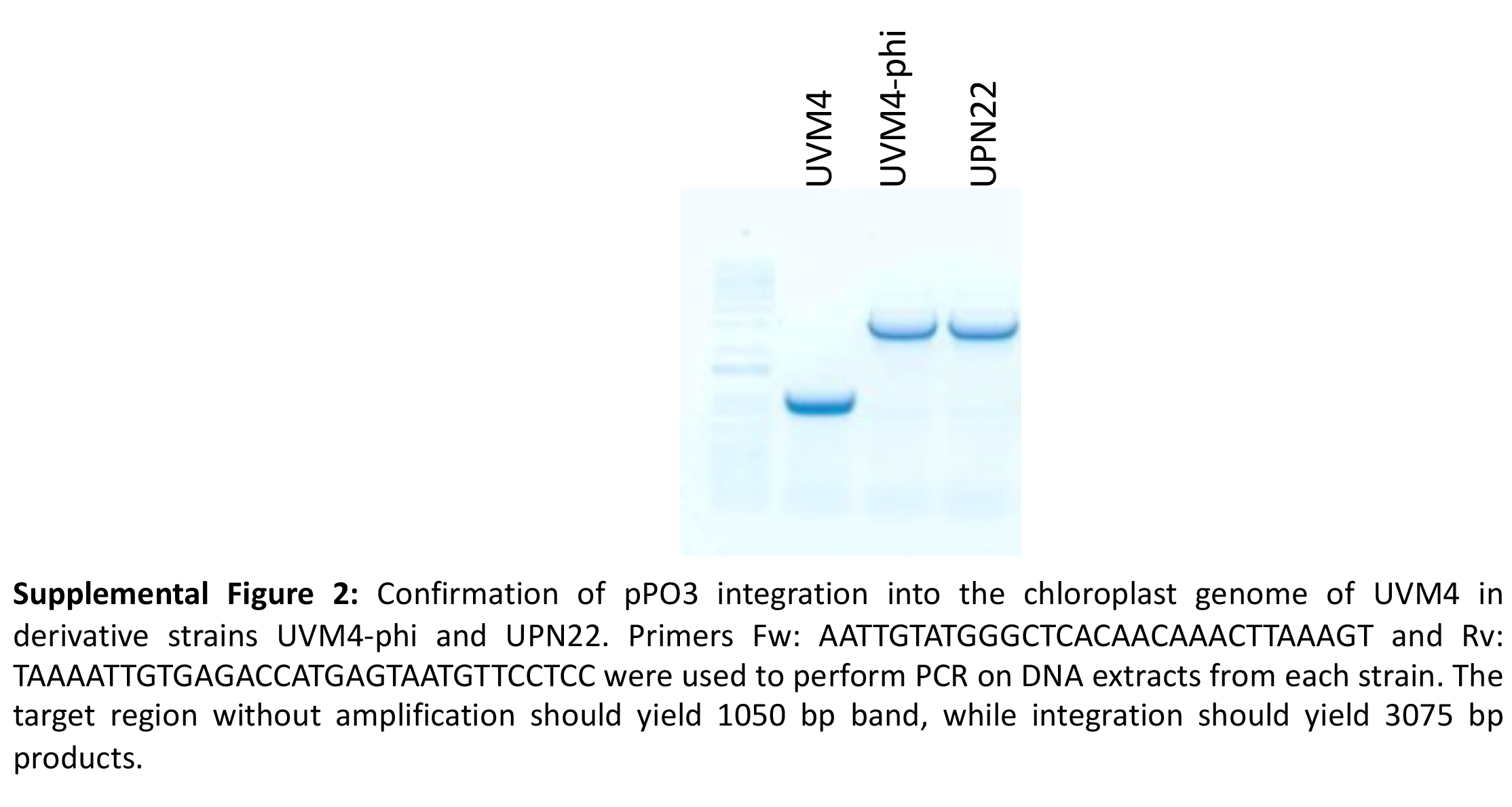

Supplement: Supplementary Figure 3 — Confirmation of pPO3 integration into the chloroplast genome of UVM4 in derivative strains UVM4-Phi and UPN22. Primers Fw: AATTGTATGGGCTCACAACAAACTTAAAGT and Rv: TAAAATTGTGAGACCATGAGTAATGTTCCTCC were used to perform PCR on DNA extracts from each strain. The target region without amplification should yield the 1,050-bp band, while integration should yield 3,075-bp products. [file Image_3.TIF]

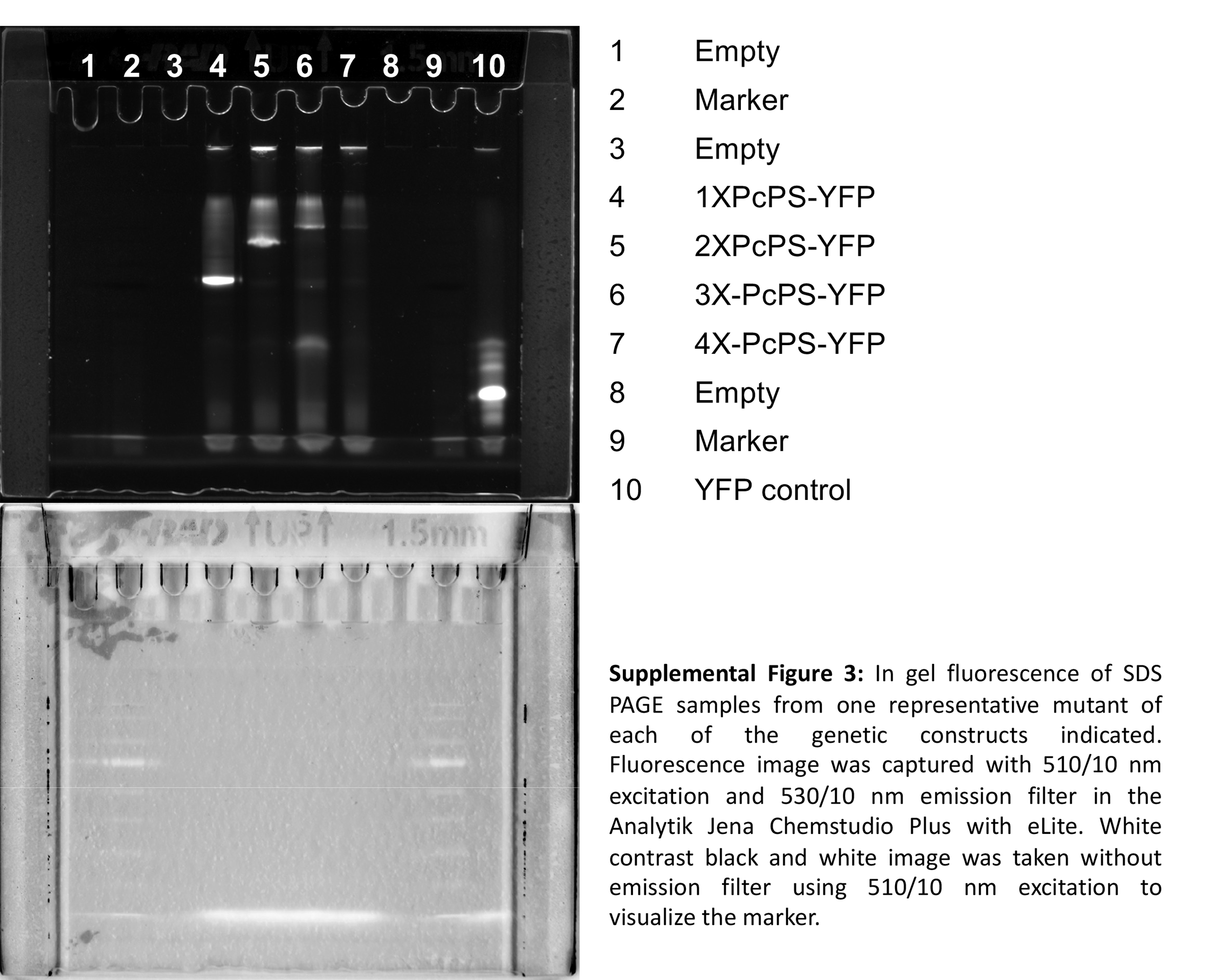

Supplement: Supplementary Figure 4 — In gel fluorescence of SDS PAGE samples from one representative mutant of each of the genetic constructs indicated. Fluorescence image was captured with 510/10-nm excitation and 530/10-nm emission filter in the AnalytikJena Chemstudio Plus with eLite. White-contrast, black-and-white image was taken without emission filter using 510/10-nm excitation to visualize the marker. [file Image_4.TIF]

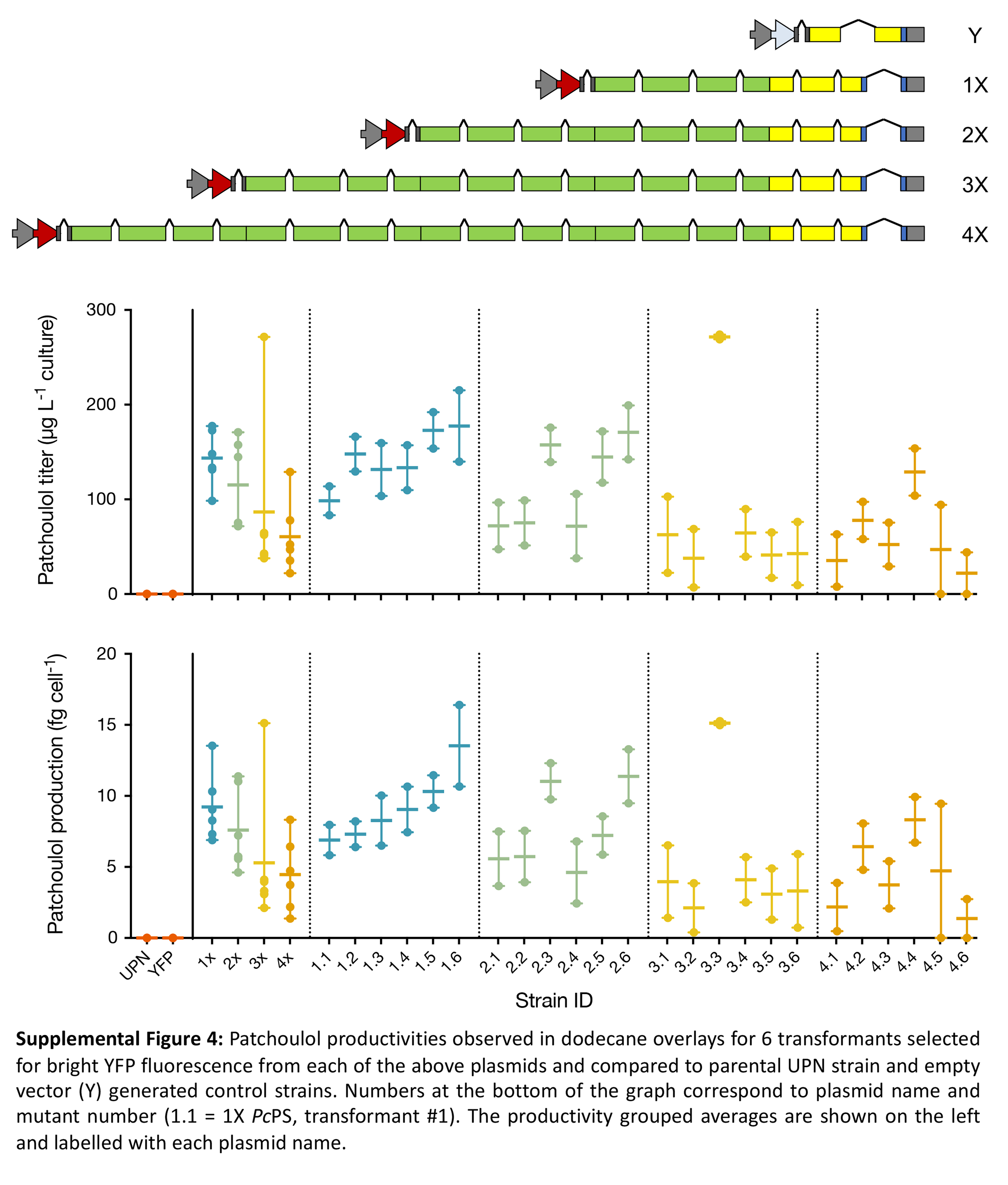

Supplement: Supplementary Figure 5 — Patchoulol productivities observed in dodecane overlays for six transformants selected for bright YFP fluorescence from each of the above plasmids and compared to parental UPN strain and empty vector (Y)-generated control strains. Numbers at the bottom of the graph correspond to the plasmid name and the mutant number (1.1 = 1X PcPS, transformant #1). Each mutant was analyzed in technical triplicates. Productivity-grouped averages are shown on the left and labeled with each plasmid name. Horizontal bars show the mean, while vertical bars show the range of values. [file Image_5.TIF]

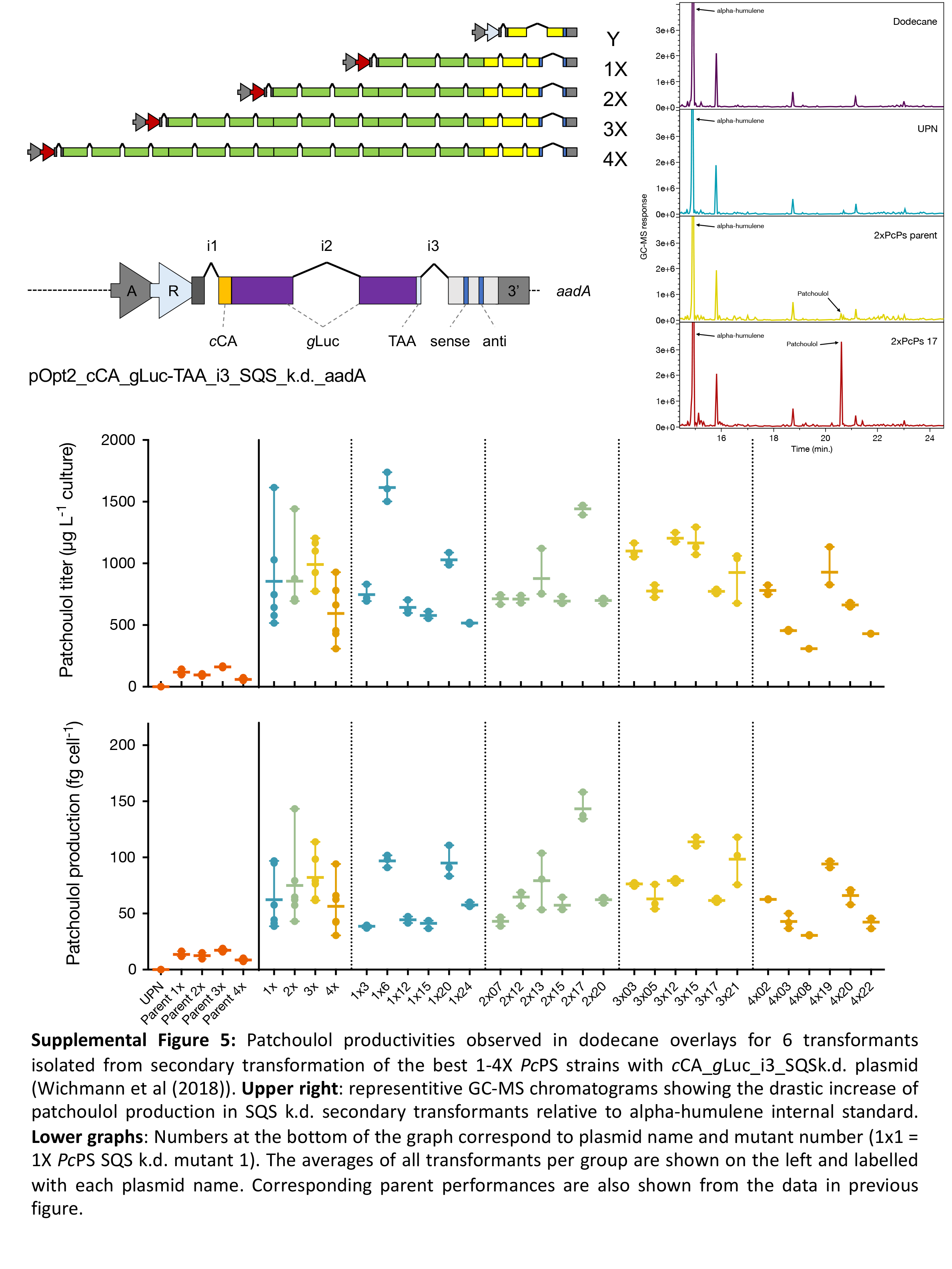

Supplement: Supplementary Figure 6 — Patchoulol productivities observed in dodecane overlays for six transformants isolated from secondary transformation of the best 1-4X PcPS strains with cCA_gLuc_i3_SQSk.d. plasmid (Wichmann et al., 2018). Upper right: representitive GC-MS chromatograms showing the drastic increase of patchoulol production in SQS k.d. secondary transformants relative to alpha-humulene internal standard. Lower graphs: Numbers at the bottom of the graph correspond to the plasmid name and the mutant number (1 x 1 = 1 X PcPS SQS k.d. mutant 1). The averages of all transformants per group are shown on the left and labeled with each plasmid name. Corresponding parent performances are also shown from the data in the previous figure. [file Image_6.TIF]
