# Supplementary material for: Combinatorial Engineering Enables Photoautotrophic Growth in High Cell Density Phosphite-Buffered Media to Support Engineered Chlamydomonas reinhardtii Bio-Production Concepts
Source: Front Microbiol. 2022 May 13;13:885840. doi: 10.3389/fmicb.2022.885840 (PMC9141048; doi:10.3389/fmicb.2022.885840)
Supplement: Supplementary file 8 [file Table_2.DOCX]

**1XPcPS Plasmid**

LOCUS Exported 9168 bp DNA circular SYN 07-JAN-2021

DEFINITION synthetic circular DNA

ACCESSION .

VERSION .

KEYWORDS .

SOURCE synthetic DNA construct

ORGANISM synthetic DNA construct

REFERENCE 1 (bases 1 to 9168)

AUTHORS jw

TITLE Direct Submission

JOURNAL Exported Feb 13, 2022 from SnapGene 5.3.3

https://www.snapgene.com

FEATURES Location/Qualifiers

source 1..9168

/organism="synthetic DNA construct"

/mol_type="other DNA"

promoter 6..272

/label=HSP70Ap promoter

/label=HSP70Ap

promoter 273..464

/label=P-bTUB2

misc_feature 359..364

/label=inserted ATANTT motif

5'UTR join(471..546,692..746)

/label=5UTR bTUB2

misc_feature 493..498

/label=rebuild ATANTT from ATATT

misc_feature 536

/label=XhoI site deleted T->A

intron 547..691

/label=rbcS2 intron 1

CDS 750..752

/codon_start=1

/label=Start

/translation="M"

CDS join(759..1196,1342..1842,1988..2461,2607..2855)

/codon_start=1

/label=Patchoulol synthase

/translation="ELYAQSVGVGAASRPLANFHPCVWGDKFIVYNPQSCQAGEREEAE

ELKVELKRELKEASDNYMRQLKMVDAIQRLGIDYLFVEDVDEALKNLFEMFDAFCKNNH

DMHATALSFRLLRQHGYRVSCEVFEKFKDGKDGFKVPNEDGAVAVLEFFEATHLRVHGE

DVLDNAFDFTRNYLESVYATLNDPTAKQVHNALNEFSFRRGLPRVEARKYISIYEQYAS

HHKGLLKLAKLDFNLVQALHRRELSEDSRWWKTLQVPTKLSFVRDRLVESYFWASGSYF

EPNYSVARMILAKGLAVLSLMDDVYDAYGTFEELQMFTDAIERWDASCLDKLPDYMKIV

YKALLDVFEEVDEELIKLGAPYRAYYGKEAMKYAARAYMEEAQWREQKHKPTTKEYMKL

ATKTCGYITLIILSCLGVEEGIVTKEAFDWVFSRPPFIEATLIIARLVNDITGHEFEKK

REHVRTAVECYMEEHKVGKQEVVSEFYNQMESAWKDINEGFLRPVEFPIPLLYLILNSV

RTLEVIYKEGDSYTHVGPAMQNIIKQLYLHPVPYGSG"

intron 1197..1341

/label=rbcS2 intron 1

intron 1843..1987

/label=rbcS2 intron 1

intron 2462..2606

/label=rbcS2 intron 1

CDS 2868..2885

/codon_start=1

/label=GSGS-Linker

/translation="GSGSGS"

CDS join(2886..3084,3230..3546,3692..3889)

/codon_start=1

/label=mVenus

/translation="VSKGEELFTGVVPILVELDGDVNGHKFSVSGEGEGDATYGKLTLK

LICTTGKLPVPWPTLVTTLGYGLQCFARYPDHMKQHDFFKSAMPEGYVQERTIFFKDDG

NYKTRAEVKFEGDTLVNRIELKGIDFKEDGNILGHKLEYNYNSHNVYITADKQKNGIKA

NFKIRHNIEDGGVQLADHYQQNTPIGDGPVLLPDNHYLSYQSKLSKDPNEKRDHMVLLE

FVTAAGITLGMDELYK"

intron 3085..3229

/label=rbcS2 intron 1

intron 3547..3691

/label=rbcS2 intron 1

CDS 3890..3907

/codon_start=1

/label=GSGS-Linker

/translation="GSGSGS"

CDS join(3920..3938,4268..4269)

/codon_start=1

/label=GSGS-Linker

/translation="GSGSGSG"

intron 3939..4267

/label=rbcS2 intron 2

CDS 4270..4293

/codon_start=1

/product="peptide that binds Strep-Tactin(R), an engineered

form of streptavidin"

/label=Strep-Tag II

/translation="WSHPQFEK"

misc_feature 4297..4530

/label=3'UTR

misc_feature 4531..4542

/label=MCS

promoter 4543..4809

/label=HSP70Ap

/label=HSP70Ap(1)

promoter join(4816..5044,5190..5191)

/label=RBCS2p promoter

/label=RBCS2p

intron 5045..5189

/label=rbcS2 intron 1

CDS 5232..6035

/codon_start=1

/label=APHVIII

/translation="MDDALRALRGRYPGCEWVVVEDGASGAGVYRLRGGGRELFVKVAA

LGAGVGLLGEAERLVWLAEVGIPVPRVVEGGGDERVAWLVTEAVPGRPASARWPREQRL

DVAVALAGLARSLHALDWERCPFDRSLAVTVPQAARAVAEGSVDLEDLDEERKGWSGER

LLAELERTRPADEDLAVCHGDLCPDNVLLDPRTCEVTGLIDVGRVGRADRHSDLALVLR

ELAHEEDPWFGPECSAAFLREYGRGWDGAVSEEKLAFYRLLDEFF"

misc_feature 6045..6283

/label=3'UTR

/label=3'UTR(1)

rep_origin complement(6743..7331)

/direction=LEFT

/label=ori

/note="high-copy-number ColE1/pMB1/pBR322/pUC origin of

replication"

CDS complement(7502..8362)

/codon_start=1

/gene="bla"

/product="beta-lactamase"

/label=AmpR

/note="confers resistance to ampicillin, carbenicillin, and

related antibiotics"

/translation="MSIQHFRVALIPFFAAFCLPVFAHPETLVKVKDAEDQLGARVGYI

ELDLNSGKILESFRPEERFPMMSTFKVLLCGAVLSRIDAGQEQLGRRIHYSQNDLVEYS

PVTEKHLTDGMTVRELCSAAITMSDNTAANLLLTTIGGPKELTAFLHNMGDHVTRLDRW

EPELNEAIPNDERDTTMPVAMATTLRKLLTGELLTLASRQQLIDWMEADKVAGPLLRSA

LPAGWFIADKSGAGERGSRGIIAALGPDGKPSRIVVIYTTGSQATMDERNRQIAEIGAS

LIKHW"

promoter complement(8363..8467)

/gene="bla"

/label=AmpR promoter

rep_origin complement(8494..8949)

/direction=LEFT

/label=f1 ori

/note="f1 bacteriophage origin of replication; arrow

indicates direction of (+) strand synthesis"

ORIGIN

1 ctagagctga ggcttgacat gattggtgcg tatgtttgta tgaagctaca ggactgattt

61 ggcgggctat gagggcgggg gaagctctgg aagggccgcg atggggcgcg cggcgtccag

121 aaggcgccat acggcccgct ggcggcaccc atccggtata aaagcccgcg accccgaacg

181 gtgacctcca ctttcagcga caaacgagca cttatacata cgcgactatt ctgccgctat

241 acataaccac tcagctagct taagatccca tcctggcact ttcttgcgct atgacacttc

301 cagcaaaagg tagggcgggc tgcgagacgg cttcccggcg ctgcatgcaa caccgatgat

361 acttatgctt cgaccccccg aagctccttc ggggctgcat gggcgctccg atgccgctcc

421 agggcgagcg ctgtttaaat agccaggccc ccgactgcaa agacaccggt attatagcga

481 gctaccaaag ccatacttca aacacctaga tcactaccac ttctacacag gccacacgag

541 cttgtggtga gtcgacgagc aagcccggcg gatcaggcag cgtgcttgca gatttgactt

601 gcaacgcccg cattgtgtcg acgaaggctt ttggctcctc tgtcgctgtc tcaagcagca

661 tctaaccctg cgtcgccgtt tccatttgca gatcgcactc cgctaagggg gcgcctcttc

721 ctcttcgttt cagtcacaac ccgcaacata tgggatccga gctgtacgcc cagagcgtgg

781 gcgtgggcgc cgccagccgc cccctggcca acttccaccc ctgcgtgtgg ggcgacaagt

841 tcatcgtgta caacccccag agctgccagg ccggcgagcg cgaggaggcc gaggagctga

901 aggtggagct gaagcgcgag ctgaaggagg ccagcgacaa ctacatgcgc cagctgaaga

961 tggtggacgc catccagcgc ctgggcatcg actacctgtt cgtggaggac gtggacgagg

1021 ccctgaagaa cctgttcgag atgttcgacg ccttctgcaa gaacaaccac gacatgcacg

1081 ccaccgccct gagcttccgc ctgctgcgcc agcacggcta ccgcgtgtcc tgcgaggtgt

1141 tcgagaagtt caaggacggc aaggacggct tcaaggtgcc caacgaggac ggcgcggtga

1201 gtcgacgagc aagcccggcg gatcaggcag cgtgcttgca gatttgactt gcaacgcccg

1261 cattgtgtcg acgaaggctt ttggctcctc tgtcgctgtc tcaagcagca tctaaccctg

1321 cgtcgccgtt tccatttgca ggtggcggtg ctggagttct tcgaggccac ccacctgcgc

1381 gtgcacggcg aggacgtgct ggacaacgcc ttcgacttca cccgcaacta cctggagagc

1441 gtgtacgcca ccctgaacga ccccaccgcc aagcaggtgc acaacgcgct gaacgagttc

1501 tccttccgcc gcggcctgcc ccgcgtggag gcccgcaagt acatcagcat ctacgagcag

1561 tacgccagcc accacaaggg cctgctgaag ctggccaagc tggacttcaa cctggtgcag

1621 gcgctgcacc gccgcgagct gtccgaggac agccgctggt ggaagaccct gcaggtgccc

1681 accaagctga gcttcgtgcg cgaccgcctg gtggagagct acttctgggc cagcggcagc

1741 tacttcgagc ccaactacag cgtggcccgc atgatcctgg cgaagggcct ggccgtgctg

1801 agcctgatgg acgacgtgta cgacgcctac ggcaccttcg aggtgagtcg acgagcaagc

1861 ccggcggatc aggcagcgtg cttgcagatt tgacttgcaa cgcccgcatt gtgtcgacga

1921 aggcttttgg ctcctctgtc gctgtctcaa gcagcatcta accctgcgtc gccgtttcca

1981 tttgcaggag ctgcagatgt tcaccgacgc gatcgagcgc tgggacgcca gctgcctgga

2041 caagctgccc gactacatga agatcgtgta caaggccctg ctggacgtgt tcgaggaggt

2101 ggacgaggag ctgatcaagc tgggcgcccc ctaccgcgcc tactacggca aggaggccat

2161 gaagtacgcc gcccgcgcct acatggagga ggcccagtgg cgcgagcaga agcacaagcc

2221 caccaccaag gagtacatga agctggcgac caagacctgc ggctacatca ccctgatcat

2281 cctgtcctgc ctgggcgtgg aggagggcat cgtgaccaag gaggcgttcg actgggtgtt

2341 cagccgcccg ccgttcatcg aggcgaccct gatcatcgcc cgcctggtca acgacatcac

2401 cggccacgag ttcgagaaga agcgcgagca cgtgcgcacc gccgtggagt gctacatgga

2461 ggtgagtcga cgagcaagcc cggcggatca ggcagcgtgc ttgcagattt gacttgcaac

2521 gcccgcattg tgtcgacgaa ggcttttggc tcctctgtcg ctgtctcaag cagcatctaa

2581 ccctgcgtcg ccgtttccat ttgcaggagc acaaggtggg caagcaggag gtggtgtccg

2641 agttctacaa ccagatggag agcgcctgga aggacatcaa cgagggcttc ctgcgccccg

2701 tggagttccc catccccctg ctgtacctga tcctgaacag cgtgcgcacc ctggaggtga

2761 tctacaagga gggcgacagc tacacccacg tgggcccggc catgcagaac atcatcaagc

2821 agctgtacct gcaccccgtg ccctacggca gcggcagatc tgacgtcggc agcggcagcg

2881 gcagcgtgag caagggcgag gagctgttca ccggcgtggt gcccatcctg gtggagctgg

2941 acggcgacgt gaacggccac aagttcagcg tgagcggcga gggcgagggc gacgccacct

3001 acggcaagct gaccctgaag ctgatctgca ccaccggcaa gctgcccgtg ccctggccca

3061 ccctggtgac caccctgggc tacggtgagt cgacgagcaa gcccggcgga tcaggcagcg

3121 tgcttgcaga tttgacttgc aacgcccgca ttgtgtcgac gaaggctttt ggctcctctg

3181 tcgctgtctc aagcagcatc taaccctgcg tcgccgtttc catttgcagg cctgcagtgc

3241 ttcgcccgct accccgacca catgaagcag cacgacttct tcaagagcgc catgcccgag

3301 ggctacgtgc aggagcgcac catcttcttc aaggacgacg gtaactacaa gacccgcgcc

3361 gaggtgaagt tcgagggcga caccctggtg aaccgcatcg agctgaaggg catcgacttc

3421 aaggaggacg gcaacatcct gggccacaag ctggagtaca actacaacag ccacaacgtg

3481 tacatcaccg ccgacaagca gaagaacggc atcaaggcca acttcaagat ccgccacaac

3541 atcgaggtga gtcgacgagc aagcccggcg gatcaggcag cgtgcttgca gatttgactt

3601 gcaacgcccg cattgtgtcg acgaaggctt ttggctcctc tgtcgctgtc tcaagcagca

3661 tctaaccctg cgtcgccgtt tccatttgca ggacggcggc gtgcagctgg ccgaccacta

3721 ccagcagaac acccccatcg gcgacggccc cgtgctgctg cccgacaacc actacctgag

3781 ctaccagagc aagctgagca aggaccccaa cgagaagcgc gaccacatgg tgctgctgga

3841 gttcgtgacc gccgccggca tcaccctggg catggacgag ctgtacaagg gcagcggcag

3901 cggcagcgat atcgaattcg gcagcggcag cggctcaggt gagcttgcgg ggttgcgagc

3961 aacactccag caacgaacag tgcccaagtc aggaatctgc agtcagcctg ggctttcggc

4021 ggctttttct tgggcaaaca gcttgcactc atgccagcgc ggcttgtcca gcctcacttg

4081 agctttccag ctgctaccag ccgggctata cgacagcgac agagccatag cgtggaatca

4141 cttatttggg ttgccgaagt agcggtcgga gcgtgagttc ttggtcaagc cgccccttat

4201 ccggttcctg tccgtgtctt tgtccctcgt tcacccttcg cggcaccctt catccccttg

4261 cttgcaggtt ggagccaccc gcagttcgag aagtaaccgc tccgtgtaaa tggaggcgct

4321 cgttgatctg agccttgccc cctgacgaac ggcggtggat ggaagatact gctctcaagt

4381 gctgaagcgg tagcttagct ccccgtttcg tgctgatcag tctttttcaa cacgtaaaaa

4441 gcggaggagt tttgcaattt tgttggttgt aacgatcctc cgttgatttt ggcctctttc

4501 tccatgggcg ggctgggcgt atttgaagcg actagtacgc gtgctgaggc ttgacatgat

4561 tggtgcgtat gtttgtatga agctacagga ctgatttggc gggctatgag ggcgggggaa

4621 gctctggaag ggccgcgatg gggcgcgcgg cgtccagaag gcgccatacg gcccgctggc

4681 ggcacccatc cggtataaaa gcccgcgacc ccgaacggtg acctccactt tcagcgacaa

4741 acgagcactt atacatacgc gactattctg ccgctataca taaccactca gctagcttaa

4801 gatcccatcc ctagggcatg ccgggcgcgc cagaaggagc gcagccaaac caggatgatg

4861 tttgatgggg tatttgagca cttgcaaccc ttatccggaa gccccctggc ccacaaaggc

4921 taggcgccaa tgcaagcagt tcgcatgcag cccctggagc ggtgccctcc tgataaaccg

4981 gccagggggc ctatgttctt tactttttta caagagaagt cactcaacat cttaaaatgg

5041 ccaggtgagt cgacgagcaa gcccggcgga tcaggcagcg tgcttgcaga tttgacttgc

5101 aacgcccgca ttgtgtcgac gaaggctttt ggctcctctg tcgctgtctc aagcagcatc

5161 taaccctgcg tcgccgtttc catttgcagg aagcttactc cgccctcccc ggtgctgaag

5221 aatttcgaag catggacgat gcgttgcgtg cactgcgggg tcggtatccc ggttgtgagt

5281 gggttgttgt ggaggatggg gcctcggggg ctggtgttta tcggcttcgg ggtggtgggc

5341 gggagttgtt tgtcaaggtg gcagctctgg gggccggggt gggcttgttg ggtgaggctg

5401 agcggctggt gtggttggcg gaggtgggga ttcccgtacc tcgtgttgtg gagggtggtg

5461 gggacgagag ggtcgcctgg ttggtcaccg aagcggttcc ggggcgtccg gccagtgcgc

5521 ggtggccgcg ggagcagcgg ctggacgtgg cggtggcgct cgcggggctc gctcgttcgc

5581 tgcacgcgct ggactgggag cggtgtccgt tcgatcgcag tctcgcggtg acggtgccgc

5641 aggcggcccg tgctgtcgct gaagggagcg tcgacttgga ggatctggac gaggagcgga

5701 aggggtggtc gggggagcgg cttctcgccg agctggagcg gactcggcct gcggacgagg

5761 atctggcggt ttgccacggt gacctgtgcc cggacaacgt gctgctcgac cctcgtacct

5821 gcgaggtgac cgggctgatc gacgtggggc gggtcggccg tgcggaccgg cactccgatc

5881 tcgcgctggt gctgcgcgag ctggcccacg aggaggaccc gtggttcggg ccggagtgtt

5941 ccgcggcgtt cctgcgggag tacgggcgcg ggtgggatgg ggcggtatcg gaggaaaagc

6001 tggcgtttta ccggctgttg gacgagttct tctgactcga gtgaccgctc cgtgtaaatg

6061 gaggcgctcg ttgatctgag ccttgccccc tgacgaacgg cggtggatgg aagatactgc

6121 tctcaagtgc tgaagcggta gcttagctcc ccgtttcgtg ctgatcagtc tttttcaaca

6181 cgtaaaaagc ggaggagttt tgcaattttg ttggttgtaa cgatcctccg ttgattttgg

6241 cctctttctc catgggcggg ctgggcgtat ttgaagcgga cccggtaccc agcttttgtt

6301 ccctttagtg agggttaatt gcgcgcttgg cgtaatcatg gtcatagctg tttcctgtgt

6361 gaaattgtta tccgctcaca attccacaca acatacgagc cggaagcata aagtgtaaag

6421 cctggggtgc ctaatgagtg agctaactca cattaattgc gttgcgctca ctgcccgctt

6481 tccagtcggg aaacctgtcg tgccagctgc attaatgaat cggccaacgc gcggggagag

6541 gcggtttgcg tattgggcgc tcttccgctt cctcgctcac tgactcgctg cgctcggtcg

6601 ttcggctgcg gcgagcggta tcagctcact caaaggcggt aatacggtta tccacagaat

6661 caggggataa cgcaggaaag aacatgtgag caaaaggcca gcaaaaggcc aggaaccgta

6721 aaaaggccgc gttgctggcg tttttccata ggctccgccc ccctgacgag catcacaaaa

6781 atcgacgctc aagtcagagg tggcgaaacc cgacaggact ataaagatac caggcgtttc

6841 cccctggaag ctccctcgtg cgctctcctg ttccgaccct gccgcttacc ggatacctgt

6901 ccgcctttct cccttcggga agcgtggcgc tttctcatag ctcacgctgt aggtatctca

6961 gttcggtgta ggtcgttcgc tccaagctgg gctgtgtgca cgaacccccc gttcagcccg

7021 accgctgcgc cttatccggt aactatcgtc ttgagtccaa cccggtaaga cacgacttat

7081 cgccactggc agcagccact ggtaacagga ttagcagagc gaggtatgta ggcggtgcta

7141 cagagttctt gaagtggtgg cctaactacg gctacactag aaggacagta tttggtatct

7201 gcgctctgct gaagccagtt accttcggaa aaagagttgg tagctcttga tccggcaaac

7261 aaaccaccgc tggtagcggt ggtttttttg tttgcaagca gcagattacg cgcagaaaaa

7321 aaggatctca agaagatcct ttgatctttt ctacggggtc tgacgctcag tggaacgaaa

7381 actcacgtta agggattttg gtcatgagat tatcaaaaag gatcttcacc tagatccttt

7441 taaattaaaa atgaagtttt aaatcaatct aaagtatata tgagtaaact tggtctgaca

7501 gttaccaatg cttaatcagt gaggcaccta tctcagcgat ctgtctattt cgttcatcca

7561 tagttgcctg actccccgtc gtgtagataa ctacgatacg ggagggctta ccatctggcc

7621 ccagtgctgc aatgataccg cgagacccac gctcaccggc tccagattta tcagcaataa

7681 accagccagc cggaagggcc gagcgcagaa gtggtcctgc aactttatcc gcctccatcc

7741 agtctattaa ttgttgccgg gaagctagag taagtagttc gccagttaat agtttgcgca

7801 acgttgttgc cattgctaca ggcatcgtgg tgtcacgctc gtcgtttggt atggcttcat

7861 tcagctccgg ttcccaacga tcaaggcgag ttacatgatc ccccatgttg tgcaaaaaag

7921 cggttagctc cttcggtcct ccgatcgttg tcagaagtaa gttggccgca gtgttatcac

7981 tcatggttat ggcagcactg cataattctc ttactgtcat gccatccgta agatgctttt

8041 ctgtgactgg tgagtactca accaagtcat tctgagaata gtgtatgcgg cgaccgagtt

8101 gctcttgccc ggcgtcaata cgggataata ccgcgccaca tagcagaact ttaaaagtgc

8161 tcatcattgg aaaacgttct tcggggcgaa aactctcaag gatcttaccg ctgttgagat

8221 ccagttcgat gtaacccact cgtgcaccca actgatcttc agcatctttt actttcacca

8281 gcgtttctgg gtgagcaaaa acaggaaggc aaaatgccgc aaaaaaggga ataagggcga

8341 cacggaaatg ttgaatactc atactcttcc tttttcaata ttattgaagc atttatcagg

8401 gttattgtct catgagcgga tacatatttg aatgtattta gaaaaataaa caaatagggg

8461 ttccgcgcac atttccccga aaagtgccac actaaattgt aagcgttaat attttgttaa

8521 aattcgcgtt aaatttttgt taaatcagct cattttttaa ccaataggcc gaaatcggca

8581 aaatccctta taaatcaaaa gaatagaccg agatagggtt gagtgttgtt ccagtttgga

8641 acaagagtcc actattaaag aacgtggact ccaacgtcaa agggcgaaaa accgtctatc

8701 agggcgatgg cccactacgt gaaccatcac cctaatcaag ttttttgggg tcgaggtgcc

8761 gtaaagcact aaatcggaac cctaaaggga gcccccgatt tagagcttga cggggaaagc

8821 cggcgaacgt ggcgagaaag gaagggaaga aagcgaaagg agcgggcgct agggcgctgg

8881 caagtgtagc ggtcacgctg cgcgtaacca ccacacccgc cgcgcttaat gcgccgctac

8941 agggcgcgtc ccattcgcca ttcaggctgc gcaactgttg ggaagggcga tcggtgcggg

9001 cctcttcgct attacgccag ctggcgaaag ggggatgtgc tgcaaggcga ttaagttggg

9061 taacgccagg gttttcccag tcacgacgtt gtaaaacgac ggccagtgag cgcgcgtaat

9121 acgactcact atagggcgaa ttggagctcc accgcggtgg cggccgct

//

**2XPcPS Plasmid**

LOCUS Exported 11271 bp DNA circular SYN 04-SEP-2021

DEFINITION synthetic circular DNA

ACCESSION .

VERSION .

KEYWORDS .

SOURCE synthetic DNA construct

ORGANISM recombinant plasmid

REFERENCE 1 (bases 1 to 11271)

AUTHORS Gordon Wellman

TITLE Direct Submission

JOURNAL Exported Feb 13, 2022 from SnapGene 5.3.3

https://www.snapgene.com

FEATURES Location/Qualifiers

source 1..11271

/organism="recombinant plasmid"

/mol_type="other DNA"

promoter 8..274

/label=HSP70Ap promoter

/label=HSP70Ap

promoter 275..466

/label=P-bTUB2

misc_feature 361..366

/label=inserted ATANTT motif

5'UTR join(473..548,694..748)

/label=5UTR bTUB2

misc_feature 495..500

/label=rebuild ATANTT from ATATT

misc_feature 538

/label=XhoI site deleted T->A

intron 549..693

/label=rbcS2 intron 1

CDS 752..754

/codon_start=1

/label=Start

/translation="M"

CDS join(761..1198,1344..1844,1990..2463,2609..2857)

/codon_start=1

/label=Patchoulol synthase

/translation="ELYAQSVGVGAASRPLANFHPCVWGDKFIVYNPQSCQAGEREEAE

ELKVELKRELKEASDNYMRQLKMVDAIQRLGIDYLFVEDVDEALKNLFEMFDAFCKNNH

DMHATALSFRLLRQHGYRVSCEVFEKFKDGKDGFKVPNEDGAVAVLEFFEATHLRVHGE

DVLDNAFDFTRNYLESVYATLNDPTAKQVHNALNEFSFRRGLPRVEARKYISIYEQYAS

HHKGLLKLAKLDFNLVQALHRRELSEDSRWWKTLQVPTKLSFVRDRLVESYFWASGSYF

EPNYSVARMILAKGLAVLSLMDDVYDAYGTFEELQMFTDAIERWDASCLDKLPDYMKIV

YKALLDVFEEVDEELIKLGAPYRAYYGKEAMKYAARAYMEEAQWREQKHKPTTKEYMKL

ATKTCGYITLIILSCLGVEEGIVTKEAFDWVFSRPPFIEATLIIARLVNDITGHEFEKK

REHVRTAVECYMEEHKVGKQEVVSEFYNQMESAWKDINEGFLRPVEFPIPLLYLILNSV

RTLEVIYKEGDSYTHVGPAMQNIIKQLYLHPVPYGSG"

intron 1199..1343

/label=rbcS2 intron 1

intron 1845..1989

/label=rbcS2 intron 1

intron 2464..2608

/label=rbcS2 intron 1

CDS join(2864..3301,3447..3947,4093..4566,4712..4960)

/codon_start=1

/label=Patchoulol synthase

/translation="ELYAQSVGVGAASRPLANFHPCVWGDKFIVYNPQSCQAGEREEAE

ELKVELKRELKEASDNYMRQLKMVDAIQRLGIDYLFVEDVDEALKNLFEMFDAFCKNNH

DMHATALSFRLLRQHGYRVSCEVFEKFKDGKDGFKVPNEDGAVAVLEFFEATHLRVHGE

DVLDNAFDFTRNYLESVYATLNDPTAKQVHNALNEFSFRRGLPRVEARKYISIYEQYAS

HHKGLLKLAKLDFNLVQALHRRELSEDSRWWKTLQVPTKLSFVRDRLVESYFWASGSYF

EPNYSVARMILAKGLAVLSLMDDVYDAYGTFEELQMFTDAIERWDASCLDKLPDYMKIV

YKALLDVFEEVDEELIKLGAPYRAYYGKEAMKYAARAYMEEAQWREQKHKPTTKEYMKL

ATKTCGYITLIILSCLGVEEGIVTKEAFDWVFSRPPFIEATLIIARLVNDITGHEFEKK

REHVRTAVECYMEEHKVGKQEVVSEFYNQMESAWKDINEGFLRPVEFPIPLLYLILNSV

RTLEVIYKEGDSYTHVGPAMQNIIKQLYLHPVPYGSG"

intron 3302..3446

/label=rbcS2 intron 1

intron 3948..4092

/label=rbcS2 intron 1

intron 4567..4711

/label=rbcS2 intron 1

CDS 4973..4990

/codon_start=1

/label=GSGS-Linker

/translation="GSGSGS"

CDS join(4991..5189,5335..5651,5797..5994)

/codon_start=1

/label=mVenus

/translation="VSKGEELFTGVVPILVELDGDVNGHKFSVSGEGEGDATYGKLTLK

LICTTGKLPVPWPTLVTTLGYGLQCFARYPDHMKQHDFFKSAMPEGYVQERTIFFKDDG

NYKTRAEVKFEGDTLVNRIELKGIDFKEDGNILGHKLEYNYNSHNVYITADKQKNGIKA

NFKIRHNIEDGGVQLADHYQQNTPIGDGPVLLPDNHYLSYQSKLSKDPNEKRDHMVLLE

FVTAAGITLGMDELYK"

intron 5190..5334

/label=rbcS2 intron 1

intron 5652..5796

/label=rbcS2 intron 1

CDS 5995..6012

/codon_start=1

/label=GSGS-Linker

/translation="GSGSGS"

CDS join(6025..6043,6373..6374)

/codon_start=1

/label=GSGS-Linker

/translation="GSGSGSG"

intron 6044..6372

/label=rbcS2 intron 2

CDS 6375..6398

/codon_start=1

/product="peptide that binds Strep-Tactin(R), an engineered

form of streptavidin"

/label=Strep-Tag II

/translation="WSHPQFEK"

misc_feature 6402..6635

/label=3'UTR

misc_feature 6636..6647

/label=MCS

promoter 6648..6914

/label=HSP70Ap

/label=HSP70Ap(1)

promoter join(6921..7149,7295..7296)

/label=RBCS2p promoter

/label=RBCS2p

intron 7150..7294

/label=rbcS2 intron 1

CDS 7337..8140

/codon_start=1

/label=APHVIII

/translation="MDDALRALRGRYPGCEWVVVEDGASGAGVYRLRGGGRELFVKVAA

LGAGVGLLGEAERLVWLAEVGIPVPRVVEGGGDERVAWLVTEAVPGRPASARWPREQRL

DVAVALAGLARSLHALDWERCPFDRSLAVTVPQAARAVAEGSVDLEDLDEERKGWSGER

LLAELERTRPADEDLAVCHGDLCPDNVLLDPRTCEVTGLIDVGRVGRADRHSDLALVLR

ELAHEEDPWFGPECSAAFLREYGRGWDGAVSEEKLAFYRLLDEFF"

misc_feature 8150..8388

/label=3'UTR

/label=3'UTR(1)

rep_origin complement(8848..9436)

/direction=LEFT

/label=ori

/note="high-copy-number ColE1/pMB1/pBR322/pUC origin of

replication"

CDS complement(9607..10467)

/codon_start=1

/gene="bla"

/product="beta-lactamase"

/label=AmpR

/note="confers resistance to ampicillin, carbenicillin, and

related antibiotics"

/translation="MSIQHFRVALIPFFAAFCLPVFAHPETLVKVKDAEDQLGARVGYI

ELDLNSGKILESFRPEERFPMMSTFKVLLCGAVLSRIDAGQEQLGRRIHYSQNDLVEYS

PVTEKHLTDGMTVRELCSAAITMSDNTAANLLLTTIGGPKELTAFLHNMGDHVTRLDRW

EPELNEAIPNDERDTTMPVAMATTLRKLLTGELLTLASRQQLIDWMEADKVAGPLLRSA

LPAGWFIADKSGAGERGSRGIIAALGPDGKPSRIVVIYTTGSQATMDERNRQIAEIGAS

LIKHW"

promoter complement(10468..10572)

/gene="bla"

/label=AmpR promoter

rep_origin complement(10599..11054)

/direction=LEFT

/label=f1 ori

/note="f1 bacteriophage origin of replication; arrow

indicates direction of (+) strand synthesis"

ORIGIN

1 ctctagagct gaggcttgac atgattggtg cgtatgtttg tatgaagcta caggactgat

61 ttggcgggct atgagggcgg gggaagctct ggaagggccg cgatggggcg cgcggcgtcc

121 agaaggcgcc atacggcccg ctggcggcac ccatccggta taaaagcccg cgaccccgaa

181 cggtgacctc cactttcagc gacaaacgag cacttataca tacgcgacta ttctgccgct

241 atacataacc actcagctag cttaagatcc catcctggca ctttcttgcg ctatgacact

301 tccagcaaaa ggtagggcgg gctgcgagac ggcttcccgg cgctgcatgc aacaccgatg

361 atacttatgc ttcgaccccc cgaagctcct tcggggctgc atgggcgctc cgatgccgct

421 ccagggcgag cgctgtttaa atagccaggc ccccgactgc aaagacaccg gtattatagc

481 gagctaccaa agccatactt caaacaccta gatcactacc acttctacac aggccacacg

541 agcttgtggt gagtcgacga gcaagcccgg cggatcaggc agcgtgcttg cagatttgac

601 ttgcaacgcc cgcattgtgt cgacgaaggc ttttggctcc tctgtcgctg tctcaagcag

661 catctaaccc tgcgtcgccg tttccatttg cagatcgcac tccgctaagg gggcgcctct

721 tcctcttcgt ttcagtcaca acccgcaaca tatgggatcc gagctgtacg cccagagcgt

781 gggcgtgggc gccgccagcc gccccctggc caacttccac ccctgcgtgt ggggcgacaa

841 gttcatcgtg tacaaccccc agagctgcca ggccggcgag cgcgaggagg ccgaggagct

901 gaaggtggag ctgaagcgcg agctgaagga ggccagcgac aactacatgc gccagctgaa

961 gatggtggac gccatccagc gcctgggcat cgactacctg ttcgtggagg acgtggacga

1021 ggccctgaag aacctgttcg agatgttcga cgccttctgc aagaacaacc acgacatgca

1081 cgccaccgcc ctgagcttcc gcctgctgcg ccagcacggc taccgcgtgt cctgcgaggt

1141 gttcgagaag ttcaaggacg gcaaggacgg cttcaaggtg cccaacgagg acggcgcggt

1201 gagtcgacga gcaagcccgg cggatcaggc agcgtgcttg cagatttgac ttgcaacgcc

1261 cgcattgtgt cgacgaaggc ttttggctcc tctgtcgctg tctcaagcag catctaaccc

1321 tgcgtcgccg tttccatttg caggtggcgg tgctggagtt cttcgaggcc acccacctgc

1381 gcgtgcacgg cgaggacgtg ctggacaacg ccttcgactt cacccgcaac tacctggaga

1441 gcgtgtacgc caccctgaac gaccccaccg ccaagcaggt gcacaacgcg ctgaacgagt

1501 tctccttccg ccgcggcctg ccccgcgtgg aggcccgcaa gtacatcagc atctacgagc

1561 agtacgccag ccaccacaag ggcctgctga agctggccaa gctggacttc aacctggtgc

1621 aggcgctgca ccgccgcgag ctgtccgagg acagccgctg gtggaagacc ctgcaggtgc

1681 ccaccaagct gagcttcgtg cgcgaccgcc tggtggagag ctacttctgg gccagcggca

1741 gctacttcga gcccaactac agcgtggccc gcatgatcct ggcgaagggc ctggccgtgc

1801 tgagcctgat ggacgacgtg tacgacgcct acggcacctt cgaggtgagt cgacgagcaa

1861 gcccggcgga tcaggcagcg tgcttgcaga tttgacttgc aacgcccgca ttgtgtcgac

1921 gaaggctttt ggctcctctg tcgctgtctc aagcagcatc taaccctgcg tcgccgtttc

1981 catttgcagg agctgcagat gttcaccgac gcgatcgagc gctgggacgc cagctgcctg

2041 gacaagctgc ccgactacat gaagatcgtg tacaaggccc tgctggacgt gttcgaggag

2101 gtggacgagg agctgatcaa gctgggcgcc ccctaccgcg cctactacgg caaggaggcc

2161 atgaagtacg ccgcccgcgc ctacatggag gaggcccagt ggcgcgagca gaagcacaag

2221 cccaccacca aggagtacat gaagctggcg accaagacct gcggctacat caccctgatc

2281 atcctgtcct gcctgggcgt ggaggagggc atcgtgacca aggaggcgtt cgactgggtg

2341 ttcagccgcc cgccgttcat cgaggcgacc ctgatcatcg cccgcctggt caacgacatc

2401 accggccacg agttcgagaa gaagcgcgag cacgtgcgca ccgccgtgga gtgctacatg

2461 gaggtgagtc gacgagcaag cccggcggat caggcagcgt gcttgcagat ttgacttgca

2521 acgcccgcat tgtgtcgacg aaggcttttg gctcctctgt cgctgtctca agcagcatct

2581 aaccctgcgt cgccgtttcc atttgcagga gcacaaggtg ggcaagcagg aggtggtgtc

2641 cgagttctac aaccagatgg agagcgcctg gaaggacatc aacgagggct tcctgcgccc

2701 cgtggagttc cccatccccc tgctgtacct gatcctgaac agcgtgcgca ccctggaggt

2761 gatctacaag gagggcgaca gctacaccca cgtgggcccg gccatgcaga acatcatcaa

2821 gcagctgtac ctgcaccccg tgccctacgg cagcggcaga tccgagctgt acgcccagag

2881 cgtgggcgtg ggcgccgcca gccgccccct ggccaacttc cacccctgcg tgtggggcga

2941 caagttcatc gtgtacaacc cccagagctg ccaggccggc gagcgcgagg aggccgagga

3001 gctgaaggtg gagctgaagc gcgagctgaa ggaggccagc gacaactaca tgcgccagct

3061 gaagatggtg gacgccatcc agcgcctggg catcgactac ctgttcgtgg aggacgtgga

3121 cgaggccctg aagaacctgt tcgagatgtt cgacgccttc tgcaagaaca accacgacat

3181 gcacgccacc gccctgagct tccgcctgct gcgccagcac ggctaccgcg tgtcctgcga

3241 ggtgttcgag aagttcaagg acggcaagga cggcttcaag gtgcccaacg aggacggcgc

3301 ggtgagtcga cgagcaagcc cggcggatca ggcagcgtgc ttgcagattt gacttgcaac

3361 gcccgcattg tgtcgacgaa ggcttttggc tcctctgtcg ctgtctcaag cagcatctaa

3421 ccctgcgtcg ccgtttccat ttgcaggtgg cggtgctgga gttcttcgag gccacccacc

3481 tgcgcgtgca cggcgaggac gtgctggaca acgccttcga cttcacccgc aactacctgg

3541 agagcgtgta cgccaccctg aacgacccca ccgccaagca ggtgcacaac gcgctgaacg

3601 agttctcctt ccgccgcggc ctgccccgcg tggaggcccg caagtacatc agcatctacg

3661 agcagtacgc cagccaccac aagggcctgc tgaagctggc caagctggac ttcaacctgg

3721 tgcaggcgct gcaccgccgc gagctgtccg aggacagccg ctggtggaag accctgcagg

3781 tgcccaccaa gctgagcttc gtgcgcgacc gcctggtgga gagctacttc tgggccagcg

3841 gcagctactt cgagcccaac tacagcgtgg cccgcatgat cctggcgaag ggcctggccg

3901 tgctgagcct gatggacgac gtgtacgacg cctacggcac cttcgaggtg agtcgacgag

3961 caagcccggc ggatcaggca gcgtgcttgc agatttgact tgcaacgccc gcattgtgtc

4021 gacgaaggct tttggctcct ctgtcgctgt ctcaagcagc atctaaccct gcgtcgccgt

4081 ttccatttgc aggagctgca gatgttcacc gacgcgatcg agcgctggga cgccagctgc

4141 ctggacaagc tgcccgacta catgaagatc gtgtacaagg ccctgctgga cgtgttcgag

4201 gaggtggacg aggagctgat caagctgggc gccccctacc gcgcctacta cggcaaggag

4261 gccatgaagt acgccgcccg cgcctacatg gaggaggccc agtggcgcga gcagaagcac

4321 aagcccacca ccaaggagta catgaagctg gcgaccaaga cctgcggcta catcaccctg

4381 atcatcctgt cctgcctggg cgtggaggag ggcatcgtga ccaaggaggc gttcgactgg

4441 gtgttcagcc gcccgccgtt catcgaggcg accctgatca tcgcccgcct ggtcaacgac

4501 atcaccggcc acgagttcga gaagaagcgc gagcacgtgc gcaccgccgt ggagtgctac

4561 atggaggtga gtcgacgagc aagcccggcg gatcaggcag cgtgcttgca gatttgactt

4621 gcaacgcccg cattgtgtcg acgaaggctt ttggctcctc tgtcgctgtc tcaagcagca

4681 tctaaccctg cgtcgccgtt tccatttgca ggagcacaag gtgggcaagc aggaggtggt

4741 gtccgagttc tacaaccaga tggagagcgc ctggaaggac atcaacgagg gcttcctgcg

4801 ccccgtggag ttccccatcc ccctgctgta cctgatcctg aacagcgtgc gcaccctgga

4861 ggtgatctac aaggagggcg acagctacac ccacgtgggc ccggccatgc agaacatcat

4921 caagcagctg tacctgcacc ccgtgcccta cggcagcggc agatctgacg tcggcagcgg

4981 cagcggcagc gtgagcaagg gcgaggagct gttcaccggc gtggtgccca tcctggtgga

5041 gctggacggc gacgtgaacg gccacaagtt cagcgtgagc ggcgagggcg agggcgacgc

5101 cacctacggc aagctgaccc tgaagctgat ctgcaccacc ggcaagctgc ccgtgccctg

5161 gcccaccctg gtgaccaccc tgggctacgg tgagtcgacg agcaagcccg gcggatcagg

5221 cagcgtgctt gcagatttga cttgcaacgc ccgcattgtg tcgacgaagg cttttggctc

5281 ctctgtcgct gtctcaagca gcatctaacc ctgcgtcgcc gtttccattt gcaggcctgc

5341 agtgcttcgc ccgctacccc gaccacatga agcagcacga cttcttcaag agcgccatgc

5401 ccgagggcta cgtgcaggag cgcaccatct tcttcaagga cgacggtaac tacaagaccc

5461 gcgccgaggt gaagttcgag ggcgacaccc tggtgaaccg catcgagctg aagggcatcg

5521 acttcaagga ggacggcaac atcctgggcc acaagctgga gtacaactac aacagccaca

5581 acgtgtacat caccgccgac aagcagaaga acggcatcaa ggccaacttc aagatccgcc

5641 acaacatcga ggtgagtcga cgagcaagcc cggcggatca ggcagcgtgc ttgcagattt

5701 gacttgcaac gcccgcattg tgtcgacgaa ggcttttggc tcctctgtcg ctgtctcaag

5761 cagcatctaa ccctgcgtcg ccgtttccat ttgcaggacg gcggcgtgca gctggccgac

5821 cactaccagc agaacacccc catcggcgac ggccccgtgc tgctgcccga caaccactac

5881 ctgagctacc agagcaagct gagcaaggac cccaacgaga agcgcgacca catggtgctg

5941 ctggagttcg tgaccgccgc cggcatcacc ctgggcatgg acgagctgta caagggcagc

6001 ggcagcggca gcgatatcga attcggcagc ggcagcggct caggtgagct tgcggggttg

6061 cgagcaacac tccagcaacg aacagtgccc aagtcaggaa tctgcagtca gcctgggctt

6121 tcggcggctt tttcttgggc aaacagcttg cactcatgcc agcgcggctt gtccagcctc

6181 acttgagctt tccagctgct accagccggg ctatacgaca gcgacagagc catagcgtgg

6241 aatcacttat ttgggttgcc gaagtagcgg tcggagcgtg agttcttggt caagccgccc

6301 cttatccggt tcctgtccgt gtctttgtcc ctcgttcacc cttcgcggca cccttcatcc

6361 ccttgcttgc aggttggagc cacccgcagt tcgagaagta accgctccgt gtaaatggag

6421 gcgctcgttg atctgagcct tgccccctga cgaacggcgg tggatggaag atactgctct

6481 caagtgctga agcggtagct tagctccccg tttcgtgctg atcagtcttt ttcaacacgt

6541 aaaaagcgga ggagttttgc aattttgttg gttgtaacga tcctccgttg attttggcct

6601 ctttctccat gggcgggctg ggcgtatttg aagcgactag tacgcgtgct gaggcttgac

6661 atgattggtg cgtatgtttg tatgaagcta caggactgat ttggcgggct atgagggcgg

6721 gggaagctct ggaagggccg cgatggggcg cgcggcgtcc agaaggcgcc atacggcccg

6781 ctggcggcac ccatccggta taaaagcccg cgaccccgaa cggtgacctc cactttcagc

6841 gacaaacgag cacttataca tacgcgacta ttctgccgct atacataacc actcagctag

6901 cttaagatcc catccctagg gcatgccggg cgcgccagaa ggagcgcagc caaaccagga

6961 tgatgtttga tggggtattt gagcacttgc aacccttatc cggaagcccc ctggcccaca

7021 aaggctaggc gccaatgcaa gcagttcgca tgcagcccct ggagcggtgc cctcctgata

7081 aaccggccag ggggcctatg ttctttactt ttttacaaga gaagtcactc aacatcttaa

7141 aatggccagg tgagtcgacg agcaagcccg gcggatcagg cagcgtgctt gcagatttga

7201 cttgcaacgc ccgcattgtg tcgacgaagg cttttggctc ctctgtcgct gtctcaagca

7261 gcatctaacc ctgcgtcgcc gtttccattt gcaggaagct tactccgccc tccccggtgc

7321 tgaagaattt cgaagcatgg acgatgcgtt gcgtgcactg cggggtcggt atcccggttg

7381 tgagtgggtt gttgtggagg atggggcctc gggggctggt gtttatcggc ttcggggtgg

7441 tgggcgggag ttgtttgtca aggtggcagc tctgggggcc ggggtgggct tgttgggtga

7501 ggctgagcgg ctggtgtggt tggcggaggt ggggattccc gtacctcgtg ttgtggaggg

7561 tggtggggac gagagggtcg cctggttggt caccgaagcg gttccggggc gtccggccag

7621 tgcgcggtgg ccgcgggagc agcggctgga cgtggcggtg gcgctcgcgg ggctcgctcg

7681 ttcgctgcac gcgctggact gggagcggtg tccgttcgat cgcagtctcg cggtgacggt

7741 gccgcaggcg gcccgtgctg tcgctgaagg gagcgtcgac ttggaggatc tggacgagga

7801 gcggaagggg tggtcggggg agcggcttct cgccgagctg gagcggactc ggcctgcgga

7861 cgaggatctg gcggtttgcc acggtgacct gtgcccggac aacgtgctgc tcgaccctcg

7921 tacctgcgag gtgaccgggc tgatcgacgt ggggcgggtc ggccgtgcgg accggcactc

7981 cgatctcgcg ctggtgctgc gcgagctggc ccacgaggag gacccgtggt tcgggccgga

8041 gtgttccgcg gcgttcctgc gggagtacgg gcgcgggtgg gatggggcgg tatcggagga

8101 aaagctggcg ttttaccggc tgttggacga gttcttctga ctcgagtgac cgctccgtgt

8161 aaatggaggc gctcgttgat ctgagccttg ccccctgacg aacggcggtg gatggaagat

8221 actgctctca agtgctgaag cggtagctta gctccccgtt tcgtgctgat cagtcttttt

8281 caacacgtaa aaagcggagg agttttgcaa ttttgttggt tgtaacgatc ctccgttgat

8341 tttggcctct ttctccatgg gcgggctggg cgtatttgaa gcggacccgg tacccagctt

8401 ttgttccctt tagtgagggt taattgcgcg cttggcgtaa tcatggtcat agctgtttcc

8461 tgtgtgaaat tgttatccgc tcacaattcc acacaacata cgagccggaa gcataaagtg

8521 taaagcctgg ggtgcctaat gagtgagcta actcacatta attgcgttgc gctcactgcc

8581 cgctttccag tcgggaaacc tgtcgtgcca gctgcattaa tgaatcggcc aacgcgcggg

8641 gagaggcggt ttgcgtattg ggcgctcttc cgcttcctcg ctcactgact cgctgcgctc

8701 ggtcgttcgg ctgcggcgag cggtatcagc tcactcaaag gcggtaatac ggttatccac

8761 agaatcaggg gataacgcag gaaagaacat gtgagcaaaa ggccagcaaa aggccaggaa

8821 ccgtaaaaag gccgcgttgc tggcgttttt ccataggctc cgcccccctg acgagcatca

8881 caaaaatcga cgctcaagtc agaggtggcg aaacccgaca ggactataaa gataccaggc

8941 gtttccccct ggaagctccc tcgtgcgctc tcctgttccg accctgccgc ttaccggata

9001 cctgtccgcc tttctccctt cgggaagcgt ggcgctttct catagctcac gctgtaggta

9061 tctcagttcg gtgtaggtcg ttcgctccaa gctgggctgt gtgcacgaac cccccgttca

9121 gcccgaccgc tgcgccttat ccggtaacta tcgtcttgag tccaacccgg taagacacga

9181 cttatcgcca ctggcagcag ccactggtaa caggattagc agagcgaggt atgtaggcgg

9241 tgctacagag ttcttgaagt ggtggcctaa ctacggctac actagaagga cagtatttgg

9301 tatctgcgct ctgctgaagc cagttacctt cggaaaaaga gttggtagct cttgatccgg

9361 caaacaaacc accgctggta gcggtggttt ttttgtttgc aagcagcaga ttacgcgcag

9421 aaaaaaagga tctcaagaag atcctttgat cttttctacg gggtctgacg ctcagtggaa

9481 cgaaaactca cgttaaggga ttttggtcat gagattatca aaaaggatct tcacctagat

9541 ccttttaaat taaaaatgaa gttttaaatc aatctaaagt atatatgagt aaacttggtc

9601 tgacagttac caatgcttaa tcagtgaggc acctatctca gcgatctgtc tatttcgttc

9661 atccatagtt gcctgactcc ccgtcgtgta gataactacg atacgggagg gcttaccatc

9721 tggccccagt gctgcaatga taccgcgaga cccacgctca ccggctccag atttatcagc

9781 aataaaccag ccagccggaa gggccgagcg cagaagtggt cctgcaactt tatccgcctc

9841 catccagtct attaattgtt gccgggaagc tagagtaagt agttcgccag ttaatagttt

9901 gcgcaacgtt gttgccattg ctacaggcat cgtggtgtca cgctcgtcgt ttggtatggc

9961 ttcattcagc tccggttccc aacgatcaag gcgagttaca tgatccccca tgttgtgcaa

10021 aaaagcggtt agctccttcg gtcctccgat cgttgtcaga agtaagttgg ccgcagtgtt

10081 atcactcatg gttatggcag cactgcataa ttctcttact gtcatgccat ccgtaagatg

10141 cttttctgtg actggtgagt actcaaccaa gtcattctga gaatagtgta tgcggcgacc

10201 gagttgctct tgcccggcgt caatacggga taataccgcg ccacatagca gaactttaaa

10261 agtgctcatc attggaaaac gttcttcggg gcgaaaactc tcaaggatct taccgctgtt

10321 gagatccagt tcgatgtaac ccactcgtgc acccaactga tcttcagcat cttttacttt

10381 caccagcgtt tctgggtgag caaaaacagg aaggcaaaat gccgcaaaaa agggaataag

10441 ggcgacacgg aaatgttgaa tactcatact cttccttttt caatattatt gaagcattta

10501 tcagggttat tgtctcatga gcggatacat atttgaatgt atttagaaaa ataaacaaat

10561 aggggttccg cgcacatttc cccgaaaagt gccacactaa attgtaagcg ttaatatttt

10621 gttaaaattc gcgttaaatt tttgttaaat cagctcattt tttaaccaat aggccgaaat

10681 cggcaaaatc ccttataaat caaaagaata gaccgagata gggttgagtg ttgttccagt

10741 ttggaacaag agtccactat taaagaacgt ggactccaac gtcaaagggc gaaaaaccgt

10801 ctatcagggc gatggcccac tacgtgaacc atcaccctaa tcaagttttt tggggtcgag

10861 gtgccgtaaa gcactaaatc ggaaccctaa agggagcccc cgatttagag cttgacgggg

10921 aaagccggcg aacgtggcga gaaaggaagg gaagaaagcg aaaggagcgg gcgctagggc

10981 gctggcaagt gtagcggtca cgctgcgcgt aaccaccaca cccgccgcgc ttaatgcgcc

11041 gctacagggc gcgtcccatt cgccattcag gctgcgcaac tgttgggaag ggcgatcggt

11101 gcgggcctct tcgctattac gccagctggc gaaaggggga tgtgctgcaa ggcgattaag

11161 ttgggtaacg ccagggtttt cccagtcacg acgttgtaaa acgacggcca gtgagcgcgc

11221 gtaatacgac tcactatagg gcgaattgga gctccaccgc ggtggcggcc g

//

**3XPcPS Plasmid**

LOCUS Exported 13374 bp DNA circular SYN 04-SEP-2021

DEFINITION synthetic circular DNA

ACCESSION .

VERSION .

KEYWORDS .

SOURCE synthetic DNA construct

ORGANISM recombinant plasmid

REFERENCE 1 (bases 1 to 13374)

AUTHORS Gordon Wellman

TITLE Direct Submission

JOURNAL Exported Feb 13, 2022 from SnapGene 5.3.3

https://www.snapgene.com

FEATURES Location/Qualifiers

source 1..13374

/organism="recombinant plasmid"

/mol_type="other DNA"

promoter 8..274

/label=HSP70Ap promoter

/label=HSP70Ap

promoter 275..466

/label=P-bTUB2

misc_feature 361..366

/label=inserted ATANTT motif

5'UTR join(473..548,694..748)

/label=5UTR bTUB2

misc_feature 495..500

/label=rebuild ATANTT from ATATT

misc_feature 538

/label=XhoI site deleted T->A

intron 549..693

/label=rbcS2 intron 1

CDS 752..754

/codon_start=1

/label=Start

/translation="M"

CDS join(761..1198,1344..1844,1990..2463,2609..2857)

/codon_start=1

/label=Patchoulol synthase

/translation="ELYAQSVGVGAASRPLANFHPCVWGDKFIVYNPQSCQAGEREEAE

ELKVELKRELKEASDNYMRQLKMVDAIQRLGIDYLFVEDVDEALKNLFEMFDAFCKNNH

DMHATALSFRLLRQHGYRVSCEVFEKFKDGKDGFKVPNEDGAVAVLEFFEATHLRVHGE

DVLDNAFDFTRNYLESVYATLNDPTAKQVHNALNEFSFRRGLPRVEARKYISIYEQYAS

HHKGLLKLAKLDFNLVQALHRRELSEDSRWWKTLQVPTKLSFVRDRLVESYFWASGSYF

EPNYSVARMILAKGLAVLSLMDDVYDAYGTFEELQMFTDAIERWDASCLDKLPDYMKIV

YKALLDVFEEVDEELIKLGAPYRAYYGKEAMKYAARAYMEEAQWREQKHKPTTKEYMKL

ATKTCGYITLIILSCLGVEEGIVTKEAFDWVFSRPPFIEATLIIARLVNDITGHEFEKK

REHVRTAVECYMEEHKVGKQEVVSEFYNQMESAWKDINEGFLRPVEFPIPLLYLILNSV

RTLEVIYKEGDSYTHVGPAMQNIIKQLYLHPVPYGSG"

intron 1199..1343

/label=rbcS2 intron 1

intron 1845..1989

/label=rbcS2 intron 1

intron 2464..2608

/label=rbcS2 intron 1

CDS join(2864..3301,3447..3947,4093..4566,4712..4960)

/codon_start=1

/label=Patchoulol synthase

/translation="ELYAQSVGVGAASRPLANFHPCVWGDKFIVYNPQSCQAGEREEAE

ELKVELKRELKEASDNYMRQLKMVDAIQRLGIDYLFVEDVDEALKNLFEMFDAFCKNNH

DMHATALSFRLLRQHGYRVSCEVFEKFKDGKDGFKVPNEDGAVAVLEFFEATHLRVHGE

DVLDNAFDFTRNYLESVYATLNDPTAKQVHNALNEFSFRRGLPRVEARKYISIYEQYAS

HHKGLLKLAKLDFNLVQALHRRELSEDSRWWKTLQVPTKLSFVRDRLVESYFWASGSYF

EPNYSVARMILAKGLAVLSLMDDVYDAYGTFEELQMFTDAIERWDASCLDKLPDYMKIV

YKALLDVFEEVDEELIKLGAPYRAYYGKEAMKYAARAYMEEAQWREQKHKPTTKEYMKL

ATKTCGYITLIILSCLGVEEGIVTKEAFDWVFSRPPFIEATLIIARLVNDITGHEFEKK

REHVRTAVECYMEEHKVGKQEVVSEFYNQMESAWKDINEGFLRPVEFPIPLLYLILNSV

RTLEVIYKEGDSYTHVGPAMQNIIKQLYLHPVPYGSG"

intron 3302..3446

/label=rbcS2 intron 1

intron 3948..4092

/label=rbcS2 intron 1

intron 4567..4711

/label=rbcS2 intron 1

CDS join(4967..5404,5550..6050,6196..6669,6815..7063)

/codon_start=1

/label=Patchoulol synthase

/translation="ELYAQSVGVGAASRPLANFHPCVWGDKFIVYNPQSCQAGEREEAE

ELKVELKRELKEASDNYMRQLKMVDAIQRLGIDYLFVEDVDEALKNLFEMFDAFCKNNH

DMHATALSFRLLRQHGYRVSCEVFEKFKDGKDGFKVPNEDGAVAVLEFFEATHLRVHGE

DVLDNAFDFTRNYLESVYATLNDPTAKQVHNALNEFSFRRGLPRVEARKYISIYEQYAS

HHKGLLKLAKLDFNLVQALHRRELSEDSRWWKTLQVPTKLSFVRDRLVESYFWASGSYF

EPNYSVARMILAKGLAVLSLMDDVYDAYGTFEELQMFTDAIERWDASCLDKLPDYMKIV

YKALLDVFEEVDEELIKLGAPYRAYYGKEAMKYAARAYMEEAQWREQKHKPTTKEYMKL

ATKTCGYITLIILSCLGVEEGIVTKEAFDWVFSRPPFIEATLIIARLVNDITGHEFEKK

REHVRTAVECYMEEHKVGKQEVVSEFYNQMESAWKDINEGFLRPVEFPIPLLYLILNSV

RTLEVIYKEGDSYTHVGPAMQNIIKQLYLHPVPYGSG"

intron 5405..5549

/label=rbcS2 intron 1

intron 6051..6195

/label=rbcS2 intron 1

intron 6670..6814

/label=rbcS2 intron 1

CDS 7076..7093

/codon_start=1

/label=GSGS-Linker

/translation="GSGSGS"

CDS join(7094..7292,7438..7754,7900..8097)

/codon_start=1

/label=mVenus

/translation="VSKGEELFTGVVPILVELDGDVNGHKFSVSGEGEGDATYGKLTLK

LICTTGKLPVPWPTLVTTLGYGLQCFARYPDHMKQHDFFKSAMPEGYVQERTIFFKDDG

NYKTRAEVKFEGDTLVNRIELKGIDFKEDGNILGHKLEYNYNSHNVYITADKQKNGIKA

NFKIRHNIEDGGVQLADHYQQNTPIGDGPVLLPDNHYLSYQSKLSKDPNEKRDHMVLLE

FVTAAGITLGMDELYK"

intron 7293..7437

/label=rbcS2 intron 1

intron 7755..7899

/label=rbcS2 intron 1

CDS 8098..8115

/codon_start=1

/label=GSGS-Linker

/translation="GSGSGS"

CDS join(8128..8146,8476..8477)

/codon_start=1

/label=GSGS-Linker

/translation="GSGSGSG"

intron 8147..8475

/label=rbcS2 intron 2

CDS 8478..8501

/codon_start=1

/product="peptide that binds Strep-Tactin(R), an engineered

form of streptavidin"

/label=Strep-Tag II

/translation="WSHPQFEK"

misc_feature 8505..8738

/label=3'UTR

misc_feature 8739..8750

/label=MCS

promoter 8751..9017

/label=HSP70Ap

/label=HSP70Ap(1)

promoter join(9024..9252,9398..9399)

/label=RBCS2p promoter

/label=RBCS2p

intron 9253..9397

/label=rbcS2 intron 1

CDS 9440..10243

/codon_start=1

/label=APHVIII

/translation="MDDALRALRGRYPGCEWVVVEDGASGAGVYRLRGGGRELFVKVAA

LGAGVGLLGEAERLVWLAEVGIPVPRVVEGGGDERVAWLVTEAVPGRPASARWPREQRL

DVAVALAGLARSLHALDWERCPFDRSLAVTVPQAARAVAEGSVDLEDLDEERKGWSGER

LLAELERTRPADEDLAVCHGDLCPDNVLLDPRTCEVTGLIDVGRVGRADRHSDLALVLR

ELAHEEDPWFGPECSAAFLREYGRGWDGAVSEEKLAFYRLLDEFF"

misc_feature 10253..10491

/label=3'UTR

/label=3'UTR(1)

rep_origin complement(10951..11539)

/direction=LEFT

/label=ori

/note="high-copy-number ColE1/pMB1/pBR322/pUC origin of

replication"

CDS complement(11710..12570)

/codon_start=1

/gene="bla"

/product="beta-lactamase"

/label=AmpR

/note="confers resistance to ampicillin, carbenicillin, and

related antibiotics"

/translation="MSIQHFRVALIPFFAAFCLPVFAHPETLVKVKDAEDQLGARVGYI

ELDLNSGKILESFRPEERFPMMSTFKVLLCGAVLSRIDAGQEQLGRRIHYSQNDLVEYS

PVTEKHLTDGMTVRELCSAAITMSDNTAANLLLTTIGGPKELTAFLHNMGDHVTRLDRW

EPELNEAIPNDERDTTMPVAMATTLRKLLTGELLTLASRQQLIDWMEADKVAGPLLRSA

LPAGWFIADKSGAGERGSRGIIAALGPDGKPSRIVVIYTTGSQATMDERNRQIAEIGAS

LIKHW"

promoter complement(12571..12675)

/gene="bla"

/label=AmpR promoter

rep_origin complement(12702..13157)

/direction=LEFT

/label=f1 ori

/note="f1 bacteriophage origin of replication; arrow

indicates direction of (+) strand synthesis"

ORIGIN

1 ctctagagct gaggcttgac atgattggtg cgtatgtttg tatgaagcta caggactgat

61 ttggcgggct atgagggcgg gggaagctct ggaagggccg cgatggggcg cgcggcgtcc

121 agaaggcgcc atacggcccg ctggcggcac ccatccggta taaaagcccg cgaccccgaa

181 cggtgacctc cactttcagc gacaaacgag cacttataca tacgcgacta ttctgccgct

241 atacataacc actcagctag cttaagatcc catcctggca ctttcttgcg ctatgacact

301 tccagcaaaa ggtagggcgg gctgcgagac ggcttcccgg cgctgcatgc aacaccgatg

361 atacttatgc ttcgaccccc cgaagctcct tcggggctgc atgggcgctc cgatgccgct

421 ccagggcgag cgctgtttaa atagccaggc ccccgactgc aaagacaccg gtattatagc

481 gagctaccaa agccatactt caaacaccta gatcactacc acttctacac aggccacacg

541 agcttgtggt gagtcgacga gcaagcccgg cggatcaggc agcgtgcttg cagatttgac

601 ttgcaacgcc cgcattgtgt cgacgaaggc ttttggctcc tctgtcgctg tctcaagcag

661 catctaaccc tgcgtcgccg tttccatttg cagatcgcac tccgctaagg gggcgcctct

721 tcctcttcgt ttcagtcaca acccgcaaca tatgggatcc gagctgtacg cccagagcgt

781 gggcgtgggc gccgccagcc gccccctggc caacttccac ccctgcgtgt ggggcgacaa

841 gttcatcgtg tacaaccccc agagctgcca ggccggcgag cgcgaggagg ccgaggagct

901 gaaggtggag ctgaagcgcg agctgaagga ggccagcgac aactacatgc gccagctgaa

961 gatggtggac gccatccagc gcctgggcat cgactacctg ttcgtggagg acgtggacga

1021 ggccctgaag aacctgttcg agatgttcga cgccttctgc aagaacaacc acgacatgca

1081 cgccaccgcc ctgagcttcc gcctgctgcg ccagcacggc taccgcgtgt cctgcgaggt

1141 gttcgagaag ttcaaggacg gcaaggacgg cttcaaggtg cccaacgagg acggcgcggt

1201 gagtcgacga gcaagcccgg cggatcaggc agcgtgcttg cagatttgac ttgcaacgcc

1261 cgcattgtgt cgacgaaggc ttttggctcc tctgtcgctg tctcaagcag catctaaccc

1321 tgcgtcgccg tttccatttg caggtggcgg tgctggagtt cttcgaggcc acccacctgc

1381 gcgtgcacgg cgaggacgtg ctggacaacg ccttcgactt cacccgcaac tacctggaga

1441 gcgtgtacgc caccctgaac gaccccaccg ccaagcaggt gcacaacgcg ctgaacgagt

1501 tctccttccg ccgcggcctg ccccgcgtgg aggcccgcaa gtacatcagc atctacgagc

1561 agtacgccag ccaccacaag ggcctgctga agctggccaa gctggacttc aacctggtgc

1621 aggcgctgca ccgccgcgag ctgtccgagg acagccgctg gtggaagacc ctgcaggtgc

1681 ccaccaagct gagcttcgtg cgcgaccgcc tggtggagag ctacttctgg gccagcggca

1741 gctacttcga gcccaactac agcgtggccc gcatgatcct ggcgaagggc ctggccgtgc

1801 tgagcctgat ggacgacgtg tacgacgcct acggcacctt cgaggtgagt cgacgagcaa

1861 gcccggcgga tcaggcagcg tgcttgcaga tttgacttgc aacgcccgca ttgtgtcgac

1921 gaaggctttt ggctcctctg tcgctgtctc aagcagcatc taaccctgcg tcgccgtttc

1981 catttgcagg agctgcagat gttcaccgac gcgatcgagc gctgggacgc cagctgcctg

2041 gacaagctgc ccgactacat gaagatcgtg tacaaggccc tgctggacgt gttcgaggag

2101 gtggacgagg agctgatcaa gctgggcgcc ccctaccgcg cctactacgg caaggaggcc

2161 atgaagtacg ccgcccgcgc ctacatggag gaggcccagt ggcgcgagca gaagcacaag

2221 cccaccacca aggagtacat gaagctggcg accaagacct gcggctacat caccctgatc

2281 atcctgtcct gcctgggcgt ggaggagggc atcgtgacca aggaggcgtt cgactgggtg

2341 ttcagccgcc cgccgttcat cgaggcgacc ctgatcatcg cccgcctggt caacgacatc

2401 accggccacg agttcgagaa gaagcgcgag cacgtgcgca ccgccgtgga gtgctacatg

2461 gaggtgagtc gacgagcaag cccggcggat caggcagcgt gcttgcagat ttgacttgca

2521 acgcccgcat tgtgtcgacg aaggcttttg gctcctctgt cgctgtctca agcagcatct

2581 aaccctgcgt cgccgtttcc atttgcagga gcacaaggtg ggcaagcagg aggtggtgtc

2641 cgagttctac aaccagatgg agagcgcctg gaaggacatc aacgagggct tcctgcgccc

2701 cgtggagttc cccatccccc tgctgtacct gatcctgaac agcgtgcgca ccctggaggt

2761 gatctacaag gagggcgaca gctacaccca cgtgggcccg gccatgcaga acatcatcaa

2821 gcagctgtac ctgcaccccg tgccctacgg cagcggcaga tccgagctgt acgcccagag

2881 cgtgggcgtg ggcgccgcca gccgccccct ggccaacttc cacccctgcg tgtggggcga

2941 caagttcatc gtgtacaacc cccagagctg ccaggccggc gagcgcgagg aggccgagga

3001 gctgaaggtg gagctgaagc gcgagctgaa ggaggccagc gacaactaca tgcgccagct

3061 gaagatggtg gacgccatcc agcgcctggg catcgactac ctgttcgtgg aggacgtgga

3121 cgaggccctg aagaacctgt tcgagatgtt cgacgccttc tgcaagaaca accacgacat

3181 gcacgccacc gccctgagct tccgcctgct gcgccagcac ggctaccgcg tgtcctgcga

3241 ggtgttcgag aagttcaagg acggcaagga cggcttcaag gtgcccaacg aggacggcgc

3301 ggtgagtcga cgagcaagcc cggcggatca ggcagcgtgc ttgcagattt gacttgcaac

3361 gcccgcattg tgtcgacgaa ggcttttggc tcctctgtcg ctgtctcaag cagcatctaa

3421 ccctgcgtcg ccgtttccat ttgcaggtgg cggtgctgga gttcttcgag gccacccacc

3481 tgcgcgtgca cggcgaggac gtgctggaca acgccttcga cttcacccgc aactacctgg

3541 agagcgtgta cgccaccctg aacgacccca ccgccaagca ggtgcacaac gcgctgaacg

3601 agttctcctt ccgccgcggc ctgccccgcg tggaggcccg caagtacatc agcatctacg

3661 agcagtacgc cagccaccac aagggcctgc tgaagctggc caagctggac ttcaacctgg

3721 tgcaggcgct gcaccgccgc gagctgtccg aggacagccg ctggtggaag accctgcagg

3781 tgcccaccaa gctgagcttc gtgcgcgacc gcctggtgga gagctacttc tgggccagcg

3841 gcagctactt cgagcccaac tacagcgtgg cccgcatgat cctggcgaag ggcctggccg

3901 tgctgagcct gatggacgac gtgtacgacg cctacggcac cttcgaggtg agtcgacgag

3961 caagcccggc ggatcaggca gcgtgcttgc agatttgact tgcaacgccc gcattgtgtc

4021 gacgaaggct tttggctcct ctgtcgctgt ctcaagcagc atctaaccct gcgtcgccgt

4081 ttccatttgc aggagctgca gatgttcacc gacgcgatcg agcgctggga cgccagctgc

4141 ctggacaagc tgcccgacta catgaagatc gtgtacaagg ccctgctgga cgtgttcgag

4201 gaggtggacg aggagctgat caagctgggc gccccctacc gcgcctacta cggcaaggag

4261 gccatgaagt acgccgcccg cgcctacatg gaggaggccc agtggcgcga gcagaagcac

4321 aagcccacca ccaaggagta catgaagctg gcgaccaaga cctgcggcta catcaccctg

4381 atcatcctgt cctgcctggg cgtggaggag ggcatcgtga ccaaggaggc gttcgactgg

4441 gtgttcagcc gcccgccgtt catcgaggcg accctgatca tcgcccgcct ggtcaacgac

4501 atcaccggcc acgagttcga gaagaagcgc gagcacgtgc gcaccgccgt ggagtgctac

4561 atggaggtga gtcgacgagc aagcccggcg gatcaggcag cgtgcttgca gatttgactt

4621 gcaacgcccg cattgtgtcg acgaaggctt ttggctcctc tgtcgctgtc tcaagcagca

4681 tctaaccctg cgtcgccgtt tccatttgca ggagcacaag gtgggcaagc aggaggtggt

4741 gtccgagttc tacaaccaga tggagagcgc ctggaaggac atcaacgagg gcttcctgcg

4801 ccccgtggag ttccccatcc ccctgctgta cctgatcctg aacagcgtgc gcaccctgga

4861 ggtgatctac aaggagggcg acagctacac ccacgtgggc ccggccatgc agaacatcat

4921 caagcagctg tacctgcacc ccgtgcccta cggcagcggc agatccgagc tgtacgccca

4981 gagcgtgggc gtgggcgccg ccagccgccc cctggccaac ttccacccct gcgtgtgggg

5041 cgacaagttc atcgtgtaca acccccagag ctgccaggcc ggcgagcgcg aggaggccga

5101 ggagctgaag gtggagctga agcgcgagct gaaggaggcc agcgacaact acatgcgcca

5161 gctgaagatg gtggacgcca tccagcgcct gggcatcgac tacctgttcg tggaggacgt

5221 ggacgaggcc ctgaagaacc tgttcgagat gttcgacgcc ttctgcaaga acaaccacga

5281 catgcacgcc accgccctga gcttccgcct gctgcgccag cacggctacc gcgtgtcctg

5341 cgaggtgttc gagaagttca aggacggcaa ggacggcttc aaggtgccca acgaggacgg

5401 cgcggtgagt cgacgagcaa gcccggcgga tcaggcagcg tgcttgcaga tttgacttgc

5461 aacgcccgca ttgtgtcgac gaaggctttt ggctcctctg tcgctgtctc aagcagcatc

5521 taaccctgcg tcgccgtttc catttgcagg tggcggtgct ggagttcttc gaggccaccc

5581 acctgcgcgt gcacggcgag gacgtgctgg acaacgcctt cgacttcacc cgcaactacc

5641 tggagagcgt gtacgccacc ctgaacgacc ccaccgccaa gcaggtgcac aacgcgctga

5701 acgagttctc cttccgccgc ggcctgcccc gcgtggaggc ccgcaagtac atcagcatct

5761 acgagcagta cgccagccac cacaagggcc tgctgaagct ggccaagctg gacttcaacc

5821 tggtgcaggc gctgcaccgc cgcgagctgt ccgaggacag ccgctggtgg aagaccctgc

5881 aggtgcccac caagctgagc ttcgtgcgcg accgcctggt ggagagctac ttctgggcca

5941 gcggcagcta cttcgagccc aactacagcg tggcccgcat gatcctggcg aagggcctgg

6001 ccgtgctgag cctgatggac gacgtgtacg acgcctacgg caccttcgag gtgagtcgac

6061 gagcaagccc ggcggatcag gcagcgtgct tgcagatttg acttgcaacg cccgcattgt

6121 gtcgacgaag gcttttggct cctctgtcgc tgtctcaagc agcatctaac cctgcgtcgc

6181 cgtttccatt tgcaggagct gcagatgttc accgacgcga tcgagcgctg ggacgccagc

6241 tgcctggaca agctgcccga ctacatgaag atcgtgtaca aggccctgct ggacgtgttc

6301 gaggaggtgg acgaggagct gatcaagctg ggcgccccct accgcgccta ctacggcaag

6361 gaggccatga agtacgccgc ccgcgcctac atggaggagg cccagtggcg cgagcagaag

6421 cacaagccca ccaccaagga gtacatgaag ctggcgacca agacctgcgg ctacatcacc

6481 ctgatcatcc tgtcctgcct gggcgtggag gagggcatcg tgaccaagga ggcgttcgac

6541 tgggtgttca gccgcccgcc gttcatcgag gcgaccctga tcatcgcccg cctggtcaac

6601 gacatcaccg gccacgagtt cgagaagaag cgcgagcacg tgcgcaccgc cgtggagtgc

6661 tacatggagg tgagtcgacg agcaagcccg gcggatcagg cagcgtgctt gcagatttga

6721 cttgcaacgc ccgcattgtg tcgacgaagg cttttggctc ctctgtcgct gtctcaagca

6781 gcatctaacc ctgcgtcgcc gtttccattt gcaggagcac aaggtgggca agcaggaggt

6841 ggtgtccgag ttctacaacc agatggagag cgcctggaag gacatcaacg agggcttcct

6901 gcgccccgtg gagttcccca tccccctgct gtacctgatc ctgaacagcg tgcgcaccct

6961 ggaggtgatc tacaaggagg gcgacagcta cacccacgtg ggcccggcca tgcagaacat

7021 catcaagcag ctgtacctgc accccgtgcc ctacggcagc ggcagatctg acgtcggcag

7081 cggcagcggc agcgtgagca agggcgagga gctgttcacc ggcgtggtgc ccatcctggt

7141 ggagctggac ggcgacgtga acggccacaa gttcagcgtg agcggcgagg gcgagggcga

7201 cgccacctac ggcaagctga ccctgaagct gatctgcacc accggcaagc tgcccgtgcc

7261 ctggcccacc ctggtgacca ccctgggcta cggtgagtcg acgagcaagc ccggcggatc

7321 aggcagcgtg cttgcagatt tgacttgcaa cgcccgcatt gtgtcgacga aggcttttgg

7381 ctcctctgtc gctgtctcaa gcagcatcta accctgcgtc gccgtttcca tttgcaggcc

7441 tgcagtgctt cgcccgctac cccgaccaca tgaagcagca cgacttcttc aagagcgcca

7501 tgcccgaggg ctacgtgcag gagcgcacca tcttcttcaa ggacgacggt aactacaaga

7561 cccgcgccga ggtgaagttc gagggcgaca ccctggtgaa ccgcatcgag ctgaagggca

7621 tcgacttcaa ggaggacggc aacatcctgg gccacaagct ggagtacaac tacaacagcc

7681 acaacgtgta catcaccgcc gacaagcaga agaacggcat caaggccaac ttcaagatcc

7741 gccacaacat cgaggtgagt cgacgagcaa gcccggcgga tcaggcagcg tgcttgcaga

7801 tttgacttgc aacgcccgca ttgtgtcgac gaaggctttt ggctcctctg tcgctgtctc

7861 aagcagcatc taaccctgcg tcgccgtttc catttgcagg acggcggcgt gcagctggcc

7921 gaccactacc agcagaacac ccccatcggc gacggccccg tgctgctgcc cgacaaccac

7981 tacctgagct accagagcaa gctgagcaag gaccccaacg agaagcgcga ccacatggtg

8041 ctgctggagt tcgtgaccgc cgccggcatc accctgggca tggacgagct gtacaagggc

8101 agcggcagcg gcagcgatat cgaattcggc agcggcagcg gctcaggtga gcttgcgggg

8161 ttgcgagcaa cactccagca acgaacagtg cccaagtcag gaatctgcag tcagcctggg

8221 ctttcggcgg ctttttcttg ggcaaacagc ttgcactcat gccagcgcgg cttgtccagc

8281 ctcacttgag ctttccagct gctaccagcc gggctatacg acagcgacag agccatagcg

8341 tggaatcact tatttgggtt gccgaagtag cggtcggagc gtgagttctt ggtcaagccg

8401 ccccttatcc ggttcctgtc cgtgtctttg tccctcgttc acccttcgcg gcacccttca

8461 tccccttgct tgcaggttgg agccacccgc agttcgagaa gtaaccgctc cgtgtaaatg

8521 gaggcgctcg ttgatctgag ccttgccccc tgacgaacgg cggtggatgg aagatactgc

8581 tctcaagtgc tgaagcggta gcttagctcc ccgtttcgtg ctgatcagtc tttttcaaca

8641 cgtaaaaagc ggaggagttt tgcaattttg ttggttgtaa cgatcctccg ttgattttgg

8701 cctctttctc catgggcggg ctgggcgtat ttgaagcgac tagtacgcgt gctgaggctt

8761 gacatgattg gtgcgtatgt ttgtatgaag ctacaggact gatttggcgg gctatgaggg

8821 cgggggaagc tctggaaggg ccgcgatggg gcgcgcggcg tccagaaggc gccatacggc

8881 ccgctggcgg cacccatccg gtataaaagc ccgcgacccc gaacggtgac ctccactttc

8941 agcgacaaac gagcacttat acatacgcga ctattctgcc gctatacata accactcagc

9001 tagcttaaga tcccatccct agggcatgcc gggcgcgcca gaaggagcgc agccaaacca

9061 ggatgatgtt tgatggggta tttgagcact tgcaaccctt atccggaagc cccctggccc

9121 acaaaggcta ggcgccaatg caagcagttc gcatgcagcc cctggagcgg tgccctcctg

9181 ataaaccggc cagggggcct atgttcttta cttttttaca agagaagtca ctcaacatct

9241 taaaatggcc aggtgagtcg acgagcaagc ccggcggatc aggcagcgtg cttgcagatt

9301 tgacttgcaa cgcccgcatt gtgtcgacga aggcttttgg ctcctctgtc gctgtctcaa

9361 gcagcatcta accctgcgtc gccgtttcca tttgcaggaa gcttactccg ccctccccgg

9421 tgctgaagaa tttcgaagca tggacgatgc gttgcgtgca ctgcggggtc ggtatcccgg

9481 ttgtgagtgg gttgttgtgg aggatggggc ctcgggggct ggtgtttatc ggcttcgggg

9541 tggtgggcgg gagttgtttg tcaaggtggc agctctgggg gccggggtgg gcttgttggg

9601 tgaggctgag cggctggtgt ggttggcgga ggtggggatt cccgtacctc gtgttgtgga

9661 gggtggtggg gacgagaggg tcgcctggtt ggtcaccgaa gcggttccgg ggcgtccggc

9721 cagtgcgcgg tggccgcggg agcagcggct ggacgtggcg gtggcgctcg cggggctcgc

9781 tcgttcgctg cacgcgctgg actgggagcg gtgtccgttc gatcgcagtc tcgcggtgac

9841 ggtgccgcag gcggcccgtg ctgtcgctga agggagcgtc gacttggagg atctggacga

9901 ggagcggaag gggtggtcgg gggagcggct tctcgccgag ctggagcgga ctcggcctgc

9961 ggacgaggat ctggcggttt gccacggtga cctgtgcccg gacaacgtgc tgctcgaccc

10021 tcgtacctgc gaggtgaccg ggctgatcga cgtggggcgg gtcggccgtg cggaccggca

10081 ctccgatctc gcgctggtgc tgcgcgagct ggcccacgag gaggacccgt ggttcgggcc

10141 ggagtgttcc gcggcgttcc tgcgggagta cgggcgcggg tgggatgggg cggtatcgga

10201 ggaaaagctg gcgttttacc ggctgttgga cgagttcttc tgactcgagt gaccgctccg

10261 tgtaaatgga ggcgctcgtt gatctgagcc ttgccccctg acgaacggcg gtggatggaa

10321 gatactgctc tcaagtgctg aagcggtagc ttagctcccc gtttcgtgct gatcagtctt

10381 tttcaacacg taaaaagcgg aggagttttg caattttgtt ggttgtaacg atcctccgtt

10441 gattttggcc tctttctcca tgggcgggct gggcgtattt gaagcggacc cggtacccag

10501 cttttgttcc ctttagtgag ggttaattgc gcgcttggcg taatcatggt catagctgtt

10561 tcctgtgtga aattgttatc cgctcacaat tccacacaac atacgagccg gaagcataaa

10621 gtgtaaagcc tggggtgcct aatgagtgag ctaactcaca ttaattgcgt tgcgctcact

10681 gcccgctttc cagtcgggaa acctgtcgtg ccagctgcat taatgaatcg gccaacgcgc

10741 ggggagaggc ggtttgcgta ttgggcgctc ttccgcttcc tcgctcactg actcgctgcg

10801 ctcggtcgtt cggctgcggc gagcggtatc agctcactca aaggcggtaa tacggttatc

10861 cacagaatca ggggataacg caggaaagaa catgtgagca aaaggccagc aaaaggccag

10921 gaaccgtaaa aaggccgcgt tgctggcgtt tttccatagg ctccgccccc ctgacgagca

10981 tcacaaaaat cgacgctcaa gtcagaggtg gcgaaacccg acaggactat aaagatacca

11041 ggcgtttccc cctggaagct ccctcgtgcg ctctcctgtt ccgaccctgc cgcttaccgg

11101 atacctgtcc gcctttctcc cttcgggaag cgtggcgctt tctcatagct cacgctgtag

11161 gtatctcagt tcggtgtagg tcgttcgctc caagctgggc tgtgtgcacg aaccccccgt

11221 tcagcccgac cgctgcgcct tatccggtaa ctatcgtctt gagtccaacc cggtaagaca

11281 cgacttatcg ccactggcag cagccactgg taacaggatt agcagagcga ggtatgtagg

11341 cggtgctaca gagttcttga agtggtggcc taactacggc tacactagaa ggacagtatt

11401 tggtatctgc gctctgctga agccagttac cttcggaaaa agagttggta gctcttgatc

11461 cggcaaacaa accaccgctg gtagcggtgg tttttttgtt tgcaagcagc agattacgcg

11521 cagaaaaaaa ggatctcaag aagatccttt gatcttttct acggggtctg acgctcagtg

11581 gaacgaaaac tcacgttaag ggattttggt catgagatta tcaaaaagga tcttcaccta

11641 gatcctttta aattaaaaat gaagttttaa atcaatctaa agtatatatg agtaaacttg

11701 gtctgacagt taccaatgct taatcagtga ggcacctatc tcagcgatct gtctatttcg

11761 ttcatccata gttgcctgac tccccgtcgt gtagataact acgatacggg agggcttacc

11821 atctggcccc agtgctgcaa tgataccgcg agacccacgc tcaccggctc cagatttatc

11881 agcaataaac cagccagccg gaagggccga gcgcagaagt ggtcctgcaa ctttatccgc

11941 ctccatccag tctattaatt gttgccggga agctagagta agtagttcgc cagttaatag

12001 tttgcgcaac gttgttgcca ttgctacagg catcgtggtg tcacgctcgt cgtttggtat

12061 ggcttcattc agctccggtt cccaacgatc aaggcgagtt acatgatccc ccatgttgtg

12121 caaaaaagcg gttagctcct tcggtcctcc gatcgttgtc agaagtaagt tggccgcagt

12181 gttatcactc atggttatgg cagcactgca taattctctt actgtcatgc catccgtaag

12241 atgcttttct gtgactggtg agtactcaac caagtcattc tgagaatagt gtatgcggcg

12301 accgagttgc tcttgcccgg cgtcaatacg ggataatacc gcgccacata gcagaacttt

12361 aaaagtgctc atcattggaa aacgttcttc ggggcgaaaa ctctcaagga tcttaccgct

12421 gttgagatcc agttcgatgt aacccactcg tgcacccaac tgatcttcag catcttttac

12481 tttcaccagc gtttctgggt gagcaaaaac aggaaggcaa aatgccgcaa aaaagggaat

12541 aagggcgaca cggaaatgtt gaatactcat actcttcctt tttcaatatt attgaagcat

12601 ttatcagggt tattgtctca tgagcggata catatttgaa tgtatttaga aaaataaaca

12661 aataggggtt ccgcgcacat ttccccgaaa agtgccacac taaattgtaa gcgttaatat

12721 tttgttaaaa ttcgcgttaa atttttgtta aatcagctca ttttttaacc aataggccga

12781 aatcggcaaa atcccttata aatcaaaaga atagaccgag atagggttga gtgttgttcc

12841 agtttggaac aagagtccac tattaaagaa cgtggactcc aacgtcaaag ggcgaaaaac

12901 cgtctatcag ggcgatggcc cactacgtga accatcaccc taatcaagtt ttttggggtc

12961 gaggtgccgt aaagcactaa atcggaaccc taaagggagc ccccgattta gagcttgacg

13021 gggaaagccg gcgaacgtgg cgagaaagga agggaagaaa gcgaaaggag cgggcgctag

13081 ggcgctggca agtgtagcgg tcacgctgcg cgtaaccacc acacccgccg cgcttaatgc

13141 gccgctacag ggcgcgtccc attcgccatt caggctgcgc aactgttggg aagggcgatc

13201 ggtgcgggcc tcttcgctat tacgccagct ggcgaaaggg ggatgtgctg caaggcgatt

13261 aagttgggta acgccagggt tttcccagtc acgacgttgt aaaacgacgg ccagtgagcg

13321 cgcgtaatac gactcactat agggcgaatt ggagctccac cgcggtggcg gccg

//

**4XPcPS plasmid**

LOCUS Exported 15477 bp DNA circular SYN 04-SEP-2021

DEFINITION synthetic circular DNA

ACCESSION .

VERSION .

KEYWORDS .

SOURCE synthetic DNA construct

ORGANISM recombinant plasmid

REFERENCE 1 (bases 1 to 15477)

AUTHORS Gordon Wellman

TITLE Direct Submission

JOURNAL Exported Feb 13, 2022 from SnapGene 5.3.3

https://www.snapgene.com

FEATURES Location/Qualifiers

source 1..15477

/organism="recombinant plasmid"

/mol_type="other DNA"

promoter 8..274

/label=HSP70Ap promoter

/label=HSP70Ap

promoter 275..466

/label=P-bTUB2

misc_feature 361..366

/label=inserted ATANTT motif

5'UTR join(473..548,694..748)

/label=5UTR bTUB2

misc_feature 495..500

/label=rebuild ATANTT from ATATT

misc_feature 538

/label=XhoI site deleted T->A

intron 549..693

/label=rbcS2 intron 1

CDS 752..754

/codon_start=1

/label=Start

/translation="M"

CDS join(761..1198,1344..1844,1990..2463,2609..2857)

/codon_start=1

/label=Patchoulol synthase

/translation="ELYAQSVGVGAASRPLANFHPCVWGDKFIVYNPQSCQAGEREEAE

ELKVELKRELKEASDNYMRQLKMVDAIQRLGIDYLFVEDVDEALKNLFEMFDAFCKNNH

DMHATALSFRLLRQHGYRVSCEVFEKFKDGKDGFKVPNEDGAVAVLEFFEATHLRVHGE

DVLDNAFDFTRNYLESVYATLNDPTAKQVHNALNEFSFRRGLPRVEARKYISIYEQYAS

HHKGLLKLAKLDFNLVQALHRRELSEDSRWWKTLQVPTKLSFVRDRLVESYFWASGSYF

EPNYSVARMILAKGLAVLSLMDDVYDAYGTFEELQMFTDAIERWDASCLDKLPDYMKIV

YKALLDVFEEVDEELIKLGAPYRAYYGKEAMKYAARAYMEEAQWREQKHKPTTKEYMKL

ATKTCGYITLIILSCLGVEEGIVTKEAFDWVFSRPPFIEATLIIARLVNDITGHEFEKK

REHVRTAVECYMEEHKVGKQEVVSEFYNQMESAWKDINEGFLRPVEFPIPLLYLILNSV

RTLEVIYKEGDSYTHVGPAMQNIIKQLYLHPVPYGSG"

intron 1199..1343

/label=rbcS2 intron 1

intron 1845..1989

/label=rbcS2 intron 1

intron 2464..2608

/label=rbcS2 intron 1

CDS join(2864..3301,3447..3947,4093..4566,4712..4960)

/codon_start=1

/label=Patchoulol synthase

/translation="ELYAQSVGVGAASRPLANFHPCVWGDKFIVYNPQSCQAGEREEAE

ELKVELKRELKEASDNYMRQLKMVDAIQRLGIDYLFVEDVDEALKNLFEMFDAFCKNNH

DMHATALSFRLLRQHGYRVSCEVFEKFKDGKDGFKVPNEDGAVAVLEFFEATHLRVHGE

DVLDNAFDFTRNYLESVYATLNDPTAKQVHNALNEFSFRRGLPRVEARKYISIYEQYAS

HHKGLLKLAKLDFNLVQALHRRELSEDSRWWKTLQVPTKLSFVRDRLVESYFWASGSYF

EPNYSVARMILAKGLAVLSLMDDVYDAYGTFEELQMFTDAIERWDASCLDKLPDYMKIV

YKALLDVFEEVDEELIKLGAPYRAYYGKEAMKYAARAYMEEAQWREQKHKPTTKEYMKL

ATKTCGYITLIILSCLGVEEGIVTKEAFDWVFSRPPFIEATLIIARLVNDITGHEFEKK

REHVRTAVECYMEEHKVGKQEVVSEFYNQMESAWKDINEGFLRPVEFPIPLLYLILNSV

RTLEVIYKEGDSYTHVGPAMQNIIKQLYLHPVPYGSG"

intron 3302..3446

/label=rbcS2 intron 1

intron 3948..4092

/label=rbcS2 intron 1

intron 4567..4711

/label=rbcS2 intron 1

CDS join(4967..5404,5550..6050,6196..6669,6815..7063)

/codon_start=1

/label=Patchoulol synthase

/translation="ELYAQSVGVGAASRPLANFHPCVWGDKFIVYNPQSCQAGEREEAE

ELKVELKRELKEASDNYMRQLKMVDAIQRLGIDYLFVEDVDEALKNLFEMFDAFCKNNH

DMHATALSFRLLRQHGYRVSCEVFEKFKDGKDGFKVPNEDGAVAVLEFFEATHLRVHGE

DVLDNAFDFTRNYLESVYATLNDPTAKQVHNALNEFSFRRGLPRVEARKYISIYEQYAS

HHKGLLKLAKLDFNLVQALHRRELSEDSRWWKTLQVPTKLSFVRDRLVESYFWASGSYF

EPNYSVARMILAKGLAVLSLMDDVYDAYGTFEELQMFTDAIERWDASCLDKLPDYMKIV

YKALLDVFEEVDEELIKLGAPYRAYYGKEAMKYAARAYMEEAQWREQKHKPTTKEYMKL

ATKTCGYITLIILSCLGVEEGIVTKEAFDWVFSRPPFIEATLIIARLVNDITGHEFEKK

REHVRTAVECYMEEHKVGKQEVVSEFYNQMESAWKDINEGFLRPVEFPIPLLYLILNSV

RTLEVIYKEGDSYTHVGPAMQNIIKQLYLHPVPYGSG"

intron 5405..5549

/label=rbcS2 intron 1

intron 6051..6195

/label=rbcS2 intron 1

intron 6670..6814

/label=rbcS2 intron 1

CDS join(7070..7507,7653..8153,8299..8772,8918..9166)

/codon_start=1

/label=Patchoulol synthase

/translation="ELYAQSVGVGAASRPLANFHPCVWGDKFIVYNPQSCQAGEREEAE

ELKVELKRELKEASDNYMRQLKMVDAIQRLGIDYLFVEDVDEALKNLFEMFDAFCKNNH

DMHATALSFRLLRQHGYRVSCEVFEKFKDGKDGFKVPNEDGAVAVLEFFEATHLRVHGE

DVLDNAFDFTRNYLESVYATLNDPTAKQVHNALNEFSFRRGLPRVEARKYISIYEQYAS

HHKGLLKLAKLDFNLVQALHRRELSEDSRWWKTLQVPTKLSFVRDRLVESYFWASGSYF

EPNYSVARMILAKGLAVLSLMDDVYDAYGTFEELQMFTDAIERWDASCLDKLPDYMKIV

YKALLDVFEEVDEELIKLGAPYRAYYGKEAMKYAARAYMEEAQWREQKHKPTTKEYMKL

ATKTCGYITLIILSCLGVEEGIVTKEAFDWVFSRPPFIEATLIIARLVNDITGHEFEKK

REHVRTAVECYMEEHKVGKQEVVSEFYNQMESAWKDINEGFLRPVEFPIPLLYLILNSV

RTLEVIYKEGDSYTHVGPAMQNIIKQLYLHPVPYGSG"

intron 7508..7652

/label=rbcS2 intron 1

intron 8154..8298

/label=rbcS2 intron 1

intron 8773..8917

/label=rbcS2 intron 1

CDS 9179..9196

/codon_start=1

/label=GSGS-Linker

/translation="GSGSGS"

CDS join(9197..9395,9541..9857,10003..10200)

/codon_start=1

/label=mVenus

/translation="VSKGEELFTGVVPILVELDGDVNGHKFSVSGEGEGDATYGKLTLK

LICTTGKLPVPWPTLVTTLGYGLQCFARYPDHMKQHDFFKSAMPEGYVQERTIFFKDDG

NYKTRAEVKFEGDTLVNRIELKGIDFKEDGNILGHKLEYNYNSHNVYITADKQKNGIKA

NFKIRHNIEDGGVQLADHYQQNTPIGDGPVLLPDNHYLSYQSKLSKDPNEKRDHMVLLE

FVTAAGITLGMDELYK"

intron 9396..9540

/label=rbcS2 intron 1

intron 9858..10002

/label=rbcS2 intron 1

CDS 10201..10218

/codon_start=1

/label=GSGS-Linker

/translation="GSGSGS"

CDS join(10231..10249,10579..10580)

/codon_start=1

/label=GSGS-Linker

/translation="GSGSGSG"

intron 10250..10578

/label=rbcS2 intron 2

CDS 10581..10604

/codon_start=1

/product="peptide that binds Strep-Tactin(R), an engineered

form of streptavidin"

/label=Strep-Tag II

/translation="WSHPQFEK"

misc_feature 10608..10841

/label=3'UTR

misc_feature 10842..10853

/label=MCS

promoter 10854..11120

/label=HSP70Ap

/label=HSP70Ap(1)

promoter join(11127..11355,11501..11502)

/label=RBCS2p promoter

/label=RBCS2p

intron 11356..11500

/label=rbcS2 intron 1

CDS 11543..12346

/codon_start=1

/label=APHVIII

/translation="MDDALRALRGRYPGCEWVVVEDGASGAGVYRLRGGGRELFVKVAA

LGAGVGLLGEAERLVWLAEVGIPVPRVVEGGGDERVAWLVTEAVPGRPASARWPREQRL

DVAVALAGLARSLHALDWERCPFDRSLAVTVPQAARAVAEGSVDLEDLDEERKGWSGER

LLAELERTRPADEDLAVCHGDLCPDNVLLDPRTCEVTGLIDVGRVGRADRHSDLALVLR

ELAHEEDPWFGPECSAAFLREYGRGWDGAVSEEKLAFYRLLDEFF"

misc_feature 12356..12594

/label=3'UTR

/label=3'UTR(1)

rep_origin complement(13054..13642)

/direction=LEFT

/label=ori

/note="high-copy-number ColE1/pMB1/pBR322/pUC origin of

replication"

CDS complement(13813..14673)

/codon_start=1

/gene="bla"

/product="beta-lactamase"

/label=AmpR

/note="confers resistance to ampicillin, carbenicillin, and

related antibiotics"

/translation="MSIQHFRVALIPFFAAFCLPVFAHPETLVKVKDAEDQLGARVGYI

ELDLNSGKILESFRPEERFPMMSTFKVLLCGAVLSRIDAGQEQLGRRIHYSQNDLVEYS

PVTEKHLTDGMTVRELCSAAITMSDNTAANLLLTTIGGPKELTAFLHNMGDHVTRLDRW

EPELNEAIPNDERDTTMPVAMATTLRKLLTGELLTLASRQQLIDWMEADKVAGPLLRSA

LPAGWFIADKSGAGERGSRGIIAALGPDGKPSRIVVIYTTGSQATMDERNRQIAEIGAS

LIKHW"

promoter complement(14674..14778)

/gene="bla"

/label=AmpR promoter

rep_origin complement(14805..15260)

/direction=LEFT

/label=f1 ori

/note="f1 bacteriophage origin of replication; arrow

indicates direction of (+) strand synthesis"

ORIGIN

1 ctctagagct gaggcttgac atgattggtg cgtatgtttg tatgaagcta caggactgat

61 ttggcgggct atgagggcgg gggaagctct ggaagggccg cgatggggcg cgcggcgtcc

121 agaaggcgcc atacggcccg ctggcggcac ccatccggta taaaagcccg cgaccccgaa

181 cggtgacctc cactttcagc gacaaacgag cacttataca tacgcgacta ttctgccgct

241 atacataacc actcagctag cttaagatcc catcctggca ctttcttgcg ctatgacact

301 tccagcaaaa ggtagggcgg gctgcgagac ggcttcccgg cgctgcatgc aacaccgatg

361 atacttatgc ttcgaccccc cgaagctcct tcggggctgc atgggcgctc cgatgccgct

421 ccagggcgag cgctgtttaa atagccaggc ccccgactgc aaagacaccg gtattatagc

481 gagctaccaa agccatactt caaacaccta gatcactacc acttctacac aggccacacg

541 agcttgtggt gagtcgacga gcaagcccgg cggatcaggc agcgtgcttg cagatttgac

601 ttgcaacgcc cgcattgtgt cgacgaaggc ttttggctcc tctgtcgctg tctcaagcag

661 catctaaccc tgcgtcgccg tttccatttg cagatcgcac tccgctaagg gggcgcctct

721 tcctcttcgt ttcagtcaca acccgcaaca tatgggatcc gagctgtacg cccagagcgt

781 gggcgtgggc gccgccagcc gccccctggc caacttccac ccctgcgtgt ggggcgacaa

841 gttcatcgtg tacaaccccc agagctgcca ggccggcgag cgcgaggagg ccgaggagct

901 gaaggtggag ctgaagcgcg agctgaagga ggccagcgac aactacatgc gccagctgaa

961 gatggtggac gccatccagc gcctgggcat cgactacctg ttcgtggagg acgtggacga

1021 ggccctgaag aacctgttcg agatgttcga cgccttctgc aagaacaacc acgacatgca

1081 cgccaccgcc ctgagcttcc gcctgctgcg ccagcacggc taccgcgtgt cctgcgaggt

1141 gttcgagaag ttcaaggacg gcaaggacgg cttcaaggtg cccaacgagg acggcgcggt

1201 gagtcgacga gcaagcccgg cggatcaggc agcgtgcttg cagatttgac ttgcaacgcc

1261 cgcattgtgt cgacgaaggc ttttggctcc tctgtcgctg tctcaagcag catctaaccc

1321 tgcgtcgccg tttccatttg caggtggcgg tgctggagtt cttcgaggcc acccacctgc

1381 gcgtgcacgg cgaggacgtg ctggacaacg ccttcgactt cacccgcaac tacctggaga

1441 gcgtgtacgc caccctgaac gaccccaccg ccaagcaggt gcacaacgcg ctgaacgagt

1501 tctccttccg ccgcggcctg ccccgcgtgg aggcccgcaa gtacatcagc atctacgagc

1561 agtacgccag ccaccacaag ggcctgctga agctggccaa gctggacttc aacctggtgc

1621 aggcgctgca ccgccgcgag ctgtccgagg acagccgctg gtggaagacc ctgcaggtgc

1681 ccaccaagct gagcttcgtg cgcgaccgcc tggtggagag ctacttctgg gccagcggca

1741 gctacttcga gcccaactac agcgtggccc gcatgatcct ggcgaagggc ctggccgtgc

1801 tgagcctgat ggacgacgtg tacgacgcct acggcacctt cgaggtgagt cgacgagcaa

1861 gcccggcgga tcaggcagcg tgcttgcaga tttgacttgc aacgcccgca ttgtgtcgac

1921 gaaggctttt ggctcctctg tcgctgtctc aagcagcatc taaccctgcg tcgccgtttc

1981 catttgcagg agctgcagat gttcaccgac gcgatcgagc gctgggacgc cagctgcctg

2041 gacaagctgc ccgactacat gaagatcgtg tacaaggccc tgctggacgt gttcgaggag

2101 gtggacgagg agctgatcaa gctgggcgcc ccctaccgcg cctactacgg caaggaggcc

2161 atgaagtacg ccgcccgcgc ctacatggag gaggcccagt ggcgcgagca gaagcacaag

2221 cccaccacca aggagtacat gaagctggcg accaagacct gcggctacat caccctgatc

2281 atcctgtcct gcctgggcgt ggaggagggc atcgtgacca aggaggcgtt cgactgggtg

2341 ttcagccgcc cgccgttcat cgaggcgacc ctgatcatcg cccgcctggt caacgacatc

2401 accggccacg agttcgagaa gaagcgcgag cacgtgcgca ccgccgtgga gtgctacatg

2461 gaggtgagtc gacgagcaag cccggcggat caggcagcgt gcttgcagat ttgacttgca

2521 acgcccgcat tgtgtcgacg aaggcttttg gctcctctgt cgctgtctca agcagcatct

2581 aaccctgcgt cgccgtttcc atttgcagga gcacaaggtg ggcaagcagg aggtggtgtc

2641 cgagttctac aaccagatgg agagcgcctg gaaggacatc aacgagggct tcctgcgccc

2701 cgtggagttc cccatccccc tgctgtacct gatcctgaac agcgtgcgca ccctggaggt

2761 gatctacaag gagggcgaca gctacaccca cgtgggcccg gccatgcaga acatcatcaa

2821 gcagctgtac ctgcaccccg tgccctacgg cagcggcaga tccgagctgt acgcccagag

2881 cgtgggcgtg ggcgccgcca gccgccccct ggccaacttc cacccctgcg tgtggggcga

2941 caagttcatc gtgtacaacc cccagagctg ccaggccggc gagcgcgagg aggccgagga

3001 gctgaaggtg gagctgaagc gcgagctgaa ggaggccagc gacaactaca tgcgccagct

3061 gaagatggtg gacgccatcc agcgcctggg catcgactac ctgttcgtgg aggacgtgga

3121 cgaggccctg aagaacctgt tcgagatgtt cgacgccttc tgcaagaaca accacgacat

3181 gcacgccacc gccctgagct tccgcctgct gcgccagcac ggctaccgcg tgtcctgcga

3241 ggtgttcgag aagttcaagg acggcaagga cggcttcaag gtgcccaacg aggacggcgc

3301 ggtgagtcga cgagcaagcc cggcggatca ggcagcgtgc ttgcagattt gacttgcaac

3361 gcccgcattg tgtcgacgaa ggcttttggc tcctctgtcg ctgtctcaag cagcatctaa

3421 ccctgcgtcg ccgtttccat ttgcaggtgg cggtgctgga gttcttcgag gccacccacc

3481 tgcgcgtgca cggcgaggac gtgctggaca acgccttcga cttcacccgc aactacctgg

3541 agagcgtgta cgccaccctg aacgacccca ccgccaagca ggtgcacaac gcgctgaacg

3601 agttctcctt ccgccgcggc ctgccccgcg tggaggcccg caagtacatc agcatctacg

3661 agcagtacgc cagccaccac aagggcctgc tgaagctggc caagctggac ttcaacctgg

3721 tgcaggcgct gcaccgccgc gagctgtccg aggacagccg ctggtggaag accctgcagg

3781 tgcccaccaa gctgagcttc gtgcgcgacc gcctggtgga gagctacttc tgggccagcg

3841 gcagctactt cgagcccaac tacagcgtgg cccgcatgat cctggcgaag ggcctggccg

3901 tgctgagcct gatggacgac gtgtacgacg cctacggcac cttcgaggtg agtcgacgag

3961 caagcccggc ggatcaggca gcgtgcttgc agatttgact tgcaacgccc gcattgtgtc

4021 gacgaaggct tttggctcct ctgtcgctgt ctcaagcagc atctaaccct gcgtcgccgt

4081 ttccatttgc aggagctgca gatgttcacc gacgcgatcg agcgctggga cgccagctgc

4141 ctggacaagc tgcccgacta catgaagatc gtgtacaagg ccctgctgga cgtgttcgag

4201 gaggtggacg aggagctgat caagctgggc gccccctacc gcgcctacta cggcaaggag

4261 gccatgaagt acgccgcccg cgcctacatg gaggaggccc agtggcgcga gcagaagcac

4321 aagcccacca ccaaggagta catgaagctg gcgaccaaga cctgcggcta catcaccctg

4381 atcatcctgt cctgcctggg cgtggaggag ggcatcgtga ccaaggaggc gttcgactgg

4441 gtgttcagcc gcccgccgtt catcgaggcg accctgatca tcgcccgcct ggtcaacgac

4501 atcaccggcc acgagttcga gaagaagcgc gagcacgtgc gcaccgccgt ggagtgctac

4561 atggaggtga gtcgacgagc aagcccggcg gatcaggcag cgtgcttgca gatttgactt

4621 gcaacgcccg cattgtgtcg acgaaggctt ttggctcctc tgtcgctgtc tcaagcagca

4681 tctaaccctg cgtcgccgtt tccatttgca ggagcacaag gtgggcaagc aggaggtggt

4741 gtccgagttc tacaaccaga tggagagcgc ctggaaggac atcaacgagg gcttcctgcg

4801 ccccgtggag ttccccatcc ccctgctgta cctgatcctg aacagcgtgc gcaccctgga

4861 ggtgatctac aaggagggcg acagctacac ccacgtgggc ccggccatgc agaacatcat

4921 caagcagctg tacctgcacc ccgtgcccta cggcagcggc agatccgagc tgtacgccca

4981 gagcgtgggc gtgggcgccg ccagccgccc cctggccaac ttccacccct gcgtgtgggg

5041 cgacaagttc atcgtgtaca acccccagag ctgccaggcc ggcgagcgcg aggaggccga

5101 ggagctgaag gtggagctga agcgcgagct gaaggaggcc agcgacaact acatgcgcca

5161 gctgaagatg gtggacgcca tccagcgcct gggcatcgac tacctgttcg tggaggacgt

5221 ggacgaggcc ctgaagaacc tgttcgagat gttcgacgcc ttctgcaaga acaaccacga

5281 catgcacgcc accgccctga gcttccgcct gctgcgccag cacggctacc gcgtgtcctg

5341 cgaggtgttc gagaagttca aggacggcaa ggacggcttc aaggtgccca acgaggacgg

5401 cgcggtgagt cgacgagcaa gcccggcgga tcaggcagcg tgcttgcaga tttgacttgc

5461 aacgcccgca ttgtgtcgac gaaggctttt ggctcctctg tcgctgtctc aagcagcatc

5521 taaccctgcg tcgccgtttc catttgcagg tggcggtgct ggagttcttc gaggccaccc

5581 acctgcgcgt gcacggcgag gacgtgctgg acaacgcctt cgacttcacc cgcaactacc

5641 tggagagcgt gtacgccacc ctgaacgacc ccaccgccaa gcaggtgcac aacgcgctga

5701 acgagttctc cttccgccgc ggcctgcccc gcgtggaggc ccgcaagtac atcagcatct

5761 acgagcagta cgccagccac cacaagggcc tgctgaagct ggccaagctg gacttcaacc

5821 tggtgcaggc gctgcaccgc cgcgagctgt ccgaggacag ccgctggtgg aagaccctgc

5881 aggtgcccac caagctgagc ttcgtgcgcg accgcctggt ggagagctac ttctgggcca

5941 gcggcagcta cttcgagccc aactacagcg tggcccgcat gatcctggcg aagggcctgg

6001 ccgtgctgag cctgatggac gacgtgtacg acgcctacgg caccttcgag gtgagtcgac

6061 gagcaagccc ggcggatcag gcagcgtgct tgcagatttg acttgcaacg cccgcattgt

6121 gtcgacgaag gcttttggct cctctgtcgc tgtctcaagc agcatctaac cctgcgtcgc

6181 cgtttccatt tgcaggagct gcagatgttc accgacgcga tcgagcgctg ggacgccagc

6241 tgcctggaca agctgcccga ctacatgaag atcgtgtaca aggccctgct ggacgtgttc

6301 gaggaggtgg acgaggagct gatcaagctg ggcgccccct accgcgccta ctacggcaag

6361 gaggccatga agtacgccgc ccgcgcctac atggaggagg cccagtggcg cgagcagaag

6421 cacaagccca ccaccaagga gtacatgaag ctggcgacca agacctgcgg ctacatcacc

6481 ctgatcatcc tgtcctgcct gggcgtggag gagggcatcg tgaccaagga ggcgttcgac

6541 tgggtgttca gccgcccgcc gttcatcgag gcgaccctga tcatcgcccg cctggtcaac

6601 gacatcaccg gccacgagtt cgagaagaag cgcgagcacg tgcgcaccgc cgtggagtgc

6661 tacatggagg tgagtcgacg agcaagcccg gcggatcagg cagcgtgctt gcagatttga

6721 cttgcaacgc ccgcattgtg tcgacgaagg cttttggctc ctctgtcgct gtctcaagca

6781 gcatctaacc ctgcgtcgcc gtttccattt gcaggagcac aaggtgggca agcaggaggt

6841 ggtgtccgag ttctacaacc agatggagag cgcctggaag gacatcaacg agggcttcct

6901 gcgccccgtg gagttcccca tccccctgct gtacctgatc ctgaacagcg tgcgcaccct

6961 ggaggtgatc tacaaggagg gcgacagcta cacccacgtg ggcccggcca tgcagaacat

7021 catcaagcag ctgtacctgc accccgtgcc ctacggcagc ggcagatccg agctgtacgc

7081 ccagagcgtg ggcgtgggcg ccgccagccg ccccctggcc aacttccacc cctgcgtgtg

7141 gggcgacaag ttcatcgtgt acaaccccca gagctgccag gccggcgagc gcgaggaggc

7201 cgaggagctg aaggtggagc tgaagcgcga gctgaaggag gccagcgaca actacatgcg

7261 ccagctgaag atggtggacg ccatccagcg cctgggcatc gactacctgt tcgtggagga

7321 cgtggacgag gccctgaaga acctgttcga gatgttcgac gccttctgca agaacaacca

7381 cgacatgcac gccaccgccc tgagcttccg cctgctgcgc cagcacggct accgcgtgtc

7441 ctgcgaggtg ttcgagaagt tcaaggacgg caaggacggc ttcaaggtgc ccaacgagga

7501 cggcgcggtg agtcgacgag caagcccggc ggatcaggca gcgtgcttgc agatttgact

7561 tgcaacgccc gcattgtgtc gacgaaggct tttggctcct ctgtcgctgt ctcaagcagc

7621 atctaaccct gcgtcgccgt ttccatttgc aggtggcggt gctggagttc ttcgaggcca

7681 cccacctgcg cgtgcacggc gaggacgtgc tggacaacgc cttcgacttc acccgcaact

7741 acctggagag cgtgtacgcc accctgaacg accccaccgc caagcaggtg cacaacgcgc

7801 tgaacgagtt ctccttccgc cgcggcctgc cccgcgtgga ggcccgcaag tacatcagca

7861 tctacgagca gtacgccagc caccacaagg gcctgctgaa gctggccaag ctggacttca

7921 acctggtgca ggcgctgcac cgccgcgagc tgtccgagga cagccgctgg tggaagaccc

7981 tgcaggtgcc caccaagctg agcttcgtgc gcgaccgcct ggtggagagc tacttctggg

8041 ccagcggcag ctacttcgag cccaactaca gcgtggcccg catgatcctg gcgaagggcc

8101 tggccgtgct gagcctgatg gacgacgtgt acgacgccta cggcaccttc gaggtgagtc

8161 gacgagcaag cccggcggat caggcagcgt gcttgcagat ttgacttgca acgcccgcat

8221 tgtgtcgacg aaggcttttg gctcctctgt cgctgtctca agcagcatct aaccctgcgt

8281 cgccgtttcc atttgcagga gctgcagatg ttcaccgacg cgatcgagcg ctgggacgcc

8341 agctgcctgg acaagctgcc cgactacatg aagatcgtgt acaaggccct gctggacgtg

8401 ttcgaggagg tggacgagga gctgatcaag ctgggcgccc cctaccgcgc ctactacggc

8461 aaggaggcca tgaagtacgc cgcccgcgcc tacatggagg aggcccagtg gcgcgagcag

8521 aagcacaagc ccaccaccaa ggagtacatg aagctggcga ccaagacctg cggctacatc

8581 accctgatca tcctgtcctg cctgggcgtg gaggagggca tcgtgaccaa ggaggcgttc

8641 gactgggtgt tcagccgccc gccgttcatc gaggcgaccc tgatcatcgc ccgcctggtc

8701 aacgacatca ccggccacga gttcgagaag aagcgcgagc acgtgcgcac cgccgtggag

8761 tgctacatgg aggtgagtcg acgagcaagc ccggcggatc aggcagcgtg cttgcagatt

8821 tgacttgcaa cgcccgcatt gtgtcgacga aggcttttgg ctcctctgtc gctgtctcaa

8881 gcagcatcta accctgcgtc gccgtttcca tttgcaggag cacaaggtgg gcaagcagga

8941 ggtggtgtcc gagttctaca accagatgga gagcgcctgg aaggacatca acgagggctt

9001 cctgcgcccc gtggagttcc ccatccccct gctgtacctg atcctgaaca gcgtgcgcac

9061 cctggaggtg atctacaagg agggcgacag ctacacccac gtgggcccgg ccatgcagaa

9121 catcatcaag cagctgtacc tgcaccccgt gccctacggc agcggcagat ctgacgtcgg

9181 cagcggcagc ggcagcgtga gcaagggcga ggagctgttc accggcgtgg tgcccatcct

9241 ggtggagctg gacggcgacg tgaacggcca caagttcagc gtgagcggcg agggcgaggg

9301 cgacgccacc tacggcaagc tgaccctgaa gctgatctgc accaccggca agctgcccgt

9361 gccctggccc accctggtga ccaccctggg ctacggtgag tcgacgagca agcccggcgg

9421 atcaggcagc gtgcttgcag atttgacttg caacgcccgc attgtgtcga cgaaggcttt

9481 tggctcctct gtcgctgtct caagcagcat ctaaccctgc gtcgccgttt ccatttgcag

9541 gcctgcagtg cttcgcccgc taccccgacc acatgaagca gcacgacttc ttcaagagcg

9601 ccatgcccga gggctacgtg caggagcgca ccatcttctt caaggacgac ggtaactaca

9661 agacccgcgc cgaggtgaag ttcgagggcg acaccctggt gaaccgcatc gagctgaagg

9721 gcatcgactt caaggaggac ggcaacatcc tgggccacaa gctggagtac aactacaaca

9781 gccacaacgt gtacatcacc gccgacaagc agaagaacgg catcaaggcc aacttcaaga

9841 tccgccacaa catcgaggtg agtcgacgag caagcccggc ggatcaggca gcgtgcttgc

9901 agatttgact tgcaacgccc gcattgtgtc gacgaaggct tttggctcct ctgtcgctgt

9961 ctcaagcagc atctaaccct gcgtcgccgt ttccatttgc aggacggcgg cgtgcagctg

10021 gccgaccact accagcagaa cacccccatc ggcgacggcc ccgtgctgct gcccgacaac

10081 cactacctga gctaccagag caagctgagc aaggacccca acgagaagcg cgaccacatg

10141 gtgctgctgg agttcgtgac cgccgccggc atcaccctgg gcatggacga gctgtacaag

10201 ggcagcggca gcggcagcga tatcgaattc ggcagcggca gcggctcagg tgagcttgcg

10261 gggttgcgag caacactcca gcaacgaaca gtgcccaagt caggaatctg cagtcagcct

10321 gggctttcgg cggctttttc ttgggcaaac agcttgcact catgccagcg cggcttgtcc

10381 agcctcactt gagctttcca gctgctacca gccgggctat acgacagcga cagagccata

10441 gcgtggaatc acttatttgg gttgccgaag tagcggtcgg agcgtgagtt cttggtcaag

10501 ccgcccctta tccggttcct gtccgtgtct ttgtccctcg ttcacccttc gcggcaccct

10561 tcatcccctt gcttgcaggt tggagccacc cgcagttcga gaagtaaccg ctccgtgtaa

10621 atggaggcgc tcgttgatct gagccttgcc ccctgacgaa cggcggtgga tggaagatac

10681 tgctctcaag tgctgaagcg gtagcttagc tccccgtttc gtgctgatca gtctttttca

10741 acacgtaaaa agcggaggag ttttgcaatt ttgttggttg taacgatcct ccgttgattt

10801 tggcctcttt ctccatgggc gggctgggcg tatttgaagc gactagtacg cgtgctgagg

10861 cttgacatga ttggtgcgta tgtttgtatg aagctacagg actgatttgg cgggctatga

10921 gggcggggga agctctggaa gggccgcgat ggggcgcgcg gcgtccagaa ggcgccatac

10981 ggcccgctgg cggcacccat ccggtataaa agcccgcgac cccgaacggt gacctccact

11041 ttcagcgaca aacgagcact tatacatacg cgactattct gccgctatac ataaccactc

11101 agctagctta agatcccatc cctagggcat gccgggcgcg ccagaaggag cgcagccaaa

11161 ccaggatgat gtttgatggg gtatttgagc acttgcaacc cttatccgga agccccctgg

11221 cccacaaagg ctaggcgcca atgcaagcag ttcgcatgca gcccctggag cggtgccctc

11281 ctgataaacc ggccaggggg cctatgttct ttactttttt acaagagaag tcactcaaca

11341 tcttaaaatg gccaggtgag tcgacgagca agcccggcgg atcaggcagc gtgcttgcag

11401 atttgacttg caacgcccgc attgtgtcga cgaaggcttt tggctcctct gtcgctgtct

11461 caagcagcat ctaaccctgc gtcgccgttt ccatttgcag gaagcttact ccgccctccc

11521 cggtgctgaa gaatttcgaa gcatggacga tgcgttgcgt gcactgcggg gtcggtatcc

11581 cggttgtgag tgggttgttg tggaggatgg ggcctcgggg gctggtgttt atcggcttcg

11641 gggtggtggg cgggagttgt ttgtcaaggt ggcagctctg ggggccgggg tgggcttgtt

11701 gggtgaggct gagcggctgg tgtggttggc ggaggtgggg attcccgtac ctcgtgttgt

11761 ggagggtggt ggggacgaga gggtcgcctg gttggtcacc gaagcggttc cggggcgtcc

11821 ggccagtgcg cggtggccgc gggagcagcg gctggacgtg gcggtggcgc tcgcggggct

11881 cgctcgttcg ctgcacgcgc tggactggga gcggtgtccg ttcgatcgca gtctcgcggt

11941 gacggtgccg caggcggccc gtgctgtcgc tgaagggagc gtcgacttgg aggatctgga

12001 cgaggagcgg aaggggtggt cgggggagcg gcttctcgcc gagctggagc ggactcggcc

12061 tgcggacgag gatctggcgg tttgccacgg tgacctgtgc ccggacaacg tgctgctcga

12121 ccctcgtacc tgcgaggtga ccgggctgat cgacgtgggg cgggtcggcc gtgcggaccg

12181 gcactccgat ctcgcgctgg tgctgcgcga gctggcccac gaggaggacc cgtggttcgg

12241 gccggagtgt tccgcggcgt tcctgcggga gtacgggcgc gggtgggatg gggcggtatc

12301 ggaggaaaag ctggcgtttt accggctgtt ggacgagttc ttctgactcg agtgaccgct

12361 ccgtgtaaat ggaggcgctc gttgatctga gccttgcccc ctgacgaacg gcggtggatg

12421 gaagatactg ctctcaagtg ctgaagcggt agcttagctc cccgtttcgt gctgatcagt

12481 ctttttcaac acgtaaaaag cggaggagtt ttgcaatttt gttggttgta acgatcctcc

12541 gttgattttg gcctctttct ccatgggcgg gctgggcgta tttgaagcgg acccggtacc

12601 cagcttttgt tccctttagt gagggttaat tgcgcgcttg gcgtaatcat ggtcatagct

12661 gtttcctgtg tgaaattgtt atccgctcac aattccacac aacatacgag ccggaagcat

12721 aaagtgtaaa gcctggggtg cctaatgagt gagctaactc acattaattg cgttgcgctc

12781 actgcccgct ttccagtcgg gaaacctgtc gtgccagctg cattaatgaa tcggccaacg

12841 cgcggggaga ggcggtttgc gtattgggcg ctcttccgct tcctcgctca ctgactcgct

12901 gcgctcggtc gttcggctgc ggcgagcggt atcagctcac tcaaaggcgg taatacggtt

12961 atccacagaa tcaggggata acgcaggaaa gaacatgtga gcaaaaggcc agcaaaaggc

13021 caggaaccgt aaaaaggccg cgttgctggc gtttttccat aggctccgcc cccctgacga

13081 gcatcacaaa aatcgacgct caagtcagag gtggcgaaac ccgacaggac tataaagata

13141 ccaggcgttt ccccctggaa gctccctcgt gcgctctcct gttccgaccc tgccgcttac

13201 cggatacctg tccgcctttc tcccttcggg aagcgtggcg ctttctcata gctcacgctg

13261 taggtatctc agttcggtgt aggtcgttcg ctccaagctg ggctgtgtgc acgaaccccc

13321 cgttcagccc gaccgctgcg ccttatccgg taactatcgt cttgagtcca acccggtaag

13381 acacgactta tcgccactgg cagcagccac tggtaacagg attagcagag cgaggtatgt

13441 aggcggtgct acagagttct tgaagtggtg gcctaactac ggctacacta gaaggacagt

13501 atttggtatc tgcgctctgc tgaagccagt taccttcgga aaaagagttg gtagctcttg

13561 atccggcaaa caaaccaccg ctggtagcgg tggttttttt gtttgcaagc agcagattac

13621 gcgcagaaaa aaaggatctc aagaagatcc tttgatcttt tctacggggt ctgacgctca

13681 gtggaacgaa aactcacgtt aagggatttt ggtcatgaga ttatcaaaaa ggatcttcac

13741 ctagatcctt ttaaattaaa aatgaagttt taaatcaatc taaagtatat atgagtaaac

13801 ttggtctgac agttaccaat gcttaatcag tgaggcacct atctcagcga tctgtctatt

13861 tcgttcatcc atagttgcct gactccccgt cgtgtagata actacgatac gggagggctt

13921 accatctggc cccagtgctg caatgatacc gcgagaccca cgctcaccgg ctccagattt

13981 atcagcaata aaccagccag ccggaagggc cgagcgcaga agtggtcctg caactttatc

14041 cgcctccatc cagtctatta attgttgccg ggaagctaga gtaagtagtt cgccagttaa

14101 tagtttgcgc aacgttgttg ccattgctac aggcatcgtg gtgtcacgct cgtcgtttgg

14161 tatggcttca ttcagctccg gttcccaacg atcaaggcga gttacatgat cccccatgtt

14221 gtgcaaaaaa gcggttagct ccttcggtcc tccgatcgtt gtcagaagta agttggccgc

14281 agtgttatca ctcatggtta tggcagcact gcataattct cttactgtca tgccatccgt

14341 aagatgcttt tctgtgactg gtgagtactc aaccaagtca ttctgagaat agtgtatgcg

14401 gcgaccgagt tgctcttgcc cggcgtcaat acgggataat accgcgccac atagcagaac

14461 tttaaaagtg ctcatcattg gaaaacgttc ttcggggcga aaactctcaa ggatcttacc

14521 gctgttgaga tccagttcga tgtaacccac tcgtgcaccc aactgatctt cagcatcttt

14581 tactttcacc agcgtttctg ggtgagcaaa aacaggaagg caaaatgccg caaaaaaggg

14641 aataagggcg acacggaaat gttgaatact catactcttc ctttttcaat attattgaag

14701 catttatcag ggttattgtc tcatgagcgg atacatattt gaatgtattt agaaaaataa

14761 acaaataggg gttccgcgca catttccccg aaaagtgcca cactaaattg taagcgttaa

14821 tattttgtta aaattcgcgt taaatttttg ttaaatcagc tcatttttta accaataggc

14881 cgaaatcggc aaaatccctt ataaatcaaa agaatagacc gagatagggt tgagtgttgt

14941 tccagtttgg aacaagagtc cactattaaa gaacgtggac tccaacgtca aagggcgaaa

15001 aaccgtctat cagggcgatg gcccactacg tgaaccatca ccctaatcaa gttttttggg

15061 gtcgaggtgc cgtaaagcac taaatcggaa ccctaaaggg agcccccgat ttagagcttg

15121 acggggaaag ccggcgaacg tggcgagaaa ggaagggaag aaagcgaaag gagcgggcgc

15181 tagggcgctg gcaagtgtag cggtcacgct gcgcgtaacc accacacccg ccgcgcttaa

15241 tgcgccgcta cagggcgcgt cccattcgcc attcaggctg cgcaactgtt gggaagggcg

15301 atcggtgcgg gcctcttcgc tattacgcca gctggcgaaa gggggatgtg ctgcaaggcg

15361 attaagttgg gtaacgccag ggttttccca gtcacgacgt tgtaaaacga cggccagtga

15421 gcgcgcgtaa tacgactcac tatagggcga attggagctc caccgcggtg gcggccg

//
