# Supplementary material for: Combinatorial Engineering Enables Photoautotrophic Growth in High Cell Density Phosphite-Buffered Media to Support Engineered Chlamydomonas reinhardtii Bio-Production Concepts
Source: Front Microbiol. 2022 May 13;13:885840. doi: 10.3389/fmicb.2022.885840 (PMC9141048; doi:10.3389/fmicb.2022.885840)
Supplement: Supplementary file 11 [file Data_Sheet_1.PDF]

TAP medium

	Mw (g/mol)	M	1	2	3	4	5	L
TRIS	121.14	0.02	2.42	4.84	7.26	9.68	12.1	g
TAP-Salts*	-	-	25	50	75	100	125	mL
P-Solution**	-	-	0.375	0.75	1.125	1.5	1.875	mL
Hutner's trace elements	-	-	1	2	3	4	5	mL
Glacial acetic acid	-	-	1	2	3	4	5	mL

Adjust final pH to 7.0 with 1M HCL. Generally, 2.2 mL per L
* For Tris-minimal medium, omit the acetic acid and titrate the final solution to pH 7.0 with HCl
** For Phi containing medium, omit P-Solution and add filter sterilised Phi-Solution after autoclaving

P-Solution (buffering phosph**ate** solution) for TAP							Notes:
	Mw (g/mol)	M	0.1	L (100 mL)	P M	Final medium P M	
K_2HPO_4	174.176	1.653	28.80	g	2.741	1.03 mM	Use 0.375 mL per 1L TAP. Can be added to TAP and autoclaved.
KH_2PO_4	136.086	1.088	14.80	g			

Phi-Solution (buffering phosph**ite**-solution) for TAPhi							Notes:
	Mw (g/mol)	M	0.1	L (100 mL)	P M	Final medium P M	
50% m/m K_2HPO_3:H_2O	158.18	1.653	35.80	mL	2.741	1.03 mM	Use 0.375 mL per 1L TAP. Add filter sterilised after autoclaved. Keep <60°C. See notes on K_2HPO_3 supplier.
KH_2PO_3	120.085	1.088	13.06	g			

Phosph**ite** solution - unbuffering for TAPhi							Notes:
	Mw (g/mol)	M	0.1	L (100 mL)	P M	Final medium P M	
KH_2PO_3	120.085	2.741	32.92	g	2.741	1.03 mM	Adapted from Sandoval-Vargas et al. 2018. Must be filter sterilised added after autoclaving to cooled medium. Keep phosphite solution and medium below 60°C

6P medium is made with the recipe found below, but replacing 6xP with 6xPhi and filter sterilizing after media cooling

Following: www.chlamycollection.org/content/uploads/2021/01/optimized-chlamy-medium-by-RAF-II.docx - Freudenberg et al. 2021

500x Stock 6xP buffered phosph**ate**-solution (6P-Buffer)							Notes:
	Mw (g/mol)	M	0.1	L (100 mL)	P M	Final medium P M	
K_2HPO_4	174.176	2.2	38.32	g	3.100	6.2 mM	Use 2 mL per 1L 6xP. Can be added to 6xP and autoclaved.
KH_2PO_4	136.086	0.9	12.25	g			

500x Stock 6xPhi buffered phosph**ite**-solution (6Phi-Buffer)							Notes:
	Mw (g/mol)	M	0.1	L (100 mL)	P M	Final medium P M	
50% m/m K_2HPO_3:H_2O	158.18	2.2	47.64	mL	3.100	6.2 mM	Use 2 mL per 1L 6xPhi. Add filter sterilised after autoclaving to cooled medium. Keep phosphite below 60°C. See notes on K_2HPO_3 supplier.
KH_2PO_3	120.085	0.9	10.81	g			

Notes

**K_2HPO_4**	Potassium monohygrogen phosph**ate** - Potassium phosph**ate** monobasic - Monopotassium phosph**ate**	
	CAS:	16788-57-1

**KH_2PO_4**	Potassium dihygrogen phosph**ate** - Potassium phosph**ate** dibasic - Dipotassium phosph**ate**	
	CAS:	7778-77-0

**K_2HPO_3**	Dipotassium hydrogenphosph**ite**	
	CAS:	13492-26-7
	Hygroscopic, so anydrous forms may be difficult to source. We have successfully used a 50% mass percentage solution (Density 1.461 g/mL) from BOC Sciences: https://www.bocsci.com/dipotassium-hydrogenphosphite-cas-13492-26-7-item-48789.html	

**KH_2PO_3**	Monopotassium phosph**ite** - Potassium dihydrogen phosph**ite**	
	CAS:	13997-65-6
	Anhydrous, sourced from BOC Sciences - https://www.bocsci.com/potassium-dihydrogen-phosphite-cas-13977-65-6-item-9855.html	
